# Controlled Acidity Gradients Enable CO_2_ Reduction to Formic Acid (Not Formate) by Molecular Electrocatalysts

**DOI:** 10.1021/acscentsci.6c00571

**Published:** 2026-07-17

**Authors:** Sergio Fernández, Pablo Fernandez, Amanda I. Arnoff, Elizabeth A. Scholer, Charlotte L. Montgomery, Jillian L. Dempsey, Thomas E. Mallouk, René Lopez, Alexander J. M. Miller

**Affiliations:** † Department of Chemistry, 2331 University of North Carolina at Chapel Hill, Chapel Hill, North Carolina 27599-3290, United States; ‡ Department of Physics and Astronomy, 2331 University of North Carolina at Chapel Hill, Chapel Hill, North Carolina 27599, United States; § Department of Chemistry, University of Pennsylvania, 231 S 34th St, Philadelphia, Pennsylvania 19104, United States

## Abstract

Neutral or basic conditions are commonly required for the selective electrochemical reduction of CO_2_, leading to the accumulation of carbonate salts and the generation of formate rather than formic acid. A generalizable strategy for obtaining formic acid (not formate) in the electroreduction of CO_2_ with molecular catalysts is introduced, based on controlling acidity gradients using a dual-electrolyte cell with a proton-exchange membrane. This approach uses anodic water oxidation as the source of protons and electrons for CO_2_ reduction to formic acid, while mitigating H_2_ evolution near the cathode and avoiding carbonate formation. Mechanistic studies, including systems modeling, provide insight into the origin of the formic acid selectivity and guide the broader implementation of this strategy in molecular electrocatalysis for CO_2_ utilization.

## Graphical abstract

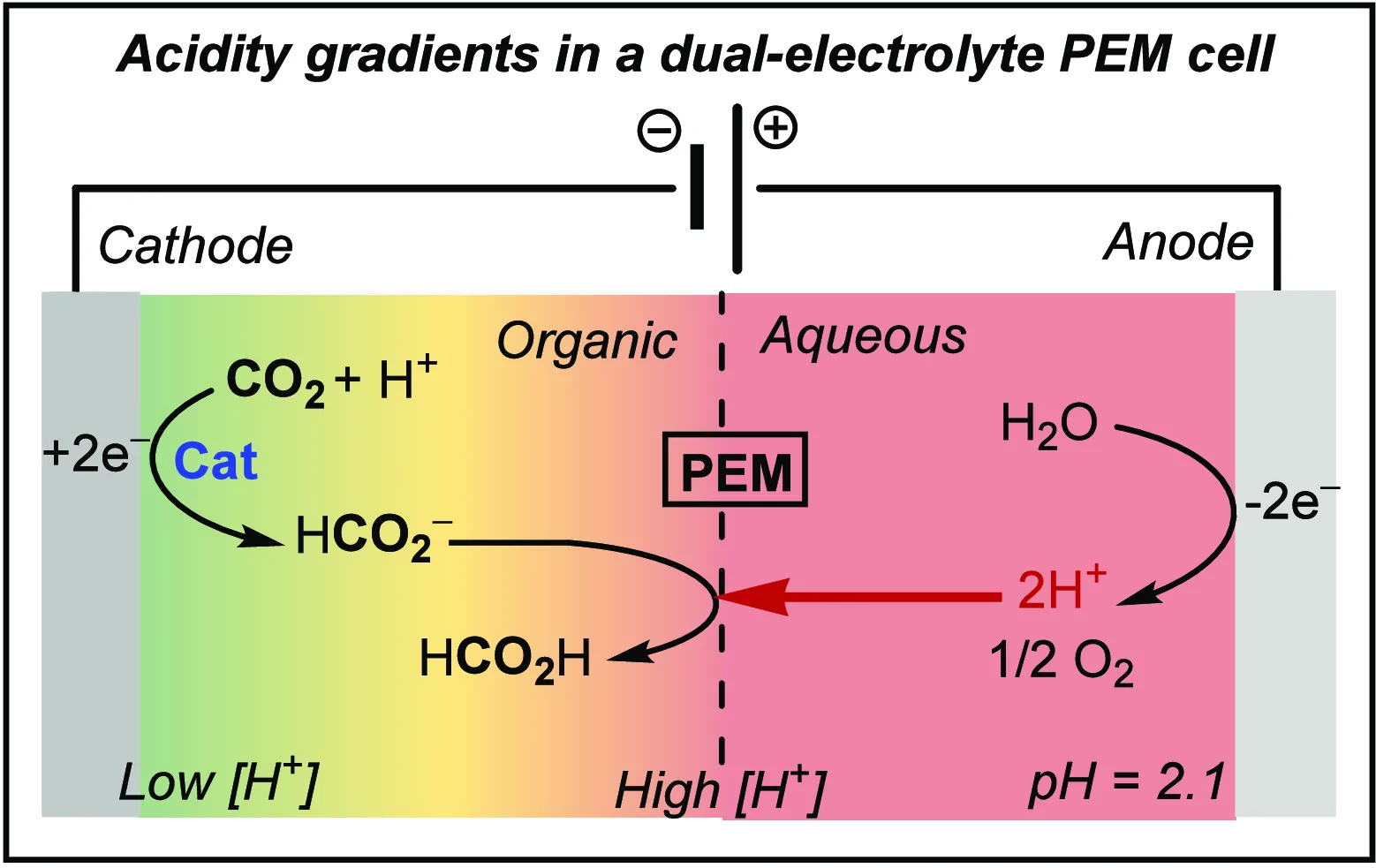

Electrochemical CO_2_ reduction (CO_2_R) is a promising approach to sustainably synthesizing chemicals and fuels.^1,2^ Achieving selective and efficient CO_2_R to liquid fuels remains a central challenge in electrocatalysis.^3^ CO is the most common product from the two-electron reduction of CO_2_.^4,5^ Formic acid (HCO_2_H) is a highly attractive two-electron reduction product, with applications as a commodity chemical, a liquid organic hydrogen carrier (LOHC),^6^ a mild reductant in organic synthesis,^7^ and an easy-to-handle CO surrogate.^8^ Moreover, HCO_2_H is a key intermediate in multicatalyst CO_2_R cascades to downstream liquid fuels such as MeOH.^9–16^

The selective formation of HCO_2_H poses a major challenge, although it might not seem that way because many papers describe the product of CO_2_ reduction as “formic acid”. In fact, most of these papers use HCO_2_H and HCO_2_^–^ interchangeably, without experimentally determining the protonation state. Inspection of the reaction conditions, however, indicates that the product must be the conjugate base, HCO_2_^–^. Almost all studies utilize neutral or even strongly basic conditions to mitigate H_2_ evolution and favor CO_2_ reduction.^5^ Unfortunately, it is HCO_2_H that is most valuable in the applications described above. In addition to the efficiency losses associated with a separate chemical step to protonate HCO_2_^–^, the basic conditions also generate large amounts of carbonate salts.^17–19^ Carbonates are detrimental in terms of carbon efficiency (wasted CO_2_) as well as energy efficiency (acidification to recycle CO_2_).^20^

There are a few *bona fide* examples of the reduction of CO_2_ to HCO_2_H. One strategy utilizes H_2_ at high pressure for the thermal hydrogenation of CO_2_ under acidic conditions (although most catalysts still operate best under basic conditions).^21,22^ Electrocatalytic CO_2_ reduction to HCO_2_H is very rare.^16,23–25^ Promising examples come from Sn and Pb electrode materials, which can achieve good Faradaic efficiency for HCO_2_H (FE_HCO2H_) under acidic conditions.^26,27^ The selectivity could originate from the catalytic sites being entirely within the electrochemical double layer, where a rapid drop in local acidity can occur (especially at high current density).^28^ Consistent with this picture, double-layer engineering using ionic liquids or alkali metal salts was a key strategy in one of the only examples of HCO_2_H formation by a molecular catalyst.^25^ The ability of homogeneous catalysts to diffuse from the electrode to the bulk and generate unwanted H_2_ may explain why molecular catalysts, which often have the highest selectivity for formate, are generally not reported to produce formic acid itself.

Biology has solved selectivity issues by compartmentalizing acid–base and redox reactivity. For example, CO_2_ fixation and H_2_O splitting reactions occur at opposite sides of the thylakoid membrane in natural photosynthesis (Figure 1A).^29^ While electron transport takes place within the membrane, a proton gradient (ΔpH ≈ 2) is established between the more basic stroma and the more acidic thylakoid space.^30^ The spatial separation prevents detrimental cross-reactivity and generates the proton motive force needed for energy storage in the form of ATP. The local acidity in protein microenvironments can also control selectivity in enzyme-catalyzed reactions. For instance, formate dehydrogenase (Fdh), which catalyzes the reversible interconversion of CO_2_ and HCO_2_^–^, features a positively charged cavity surrounding the active site that facilitates interactions with HCO_2_^–^ and generates a drop in local acidity which allows electrocatalytic CO_2_R even in an acidic medium (Figure 1B).^31,32^

**Figure 1:**
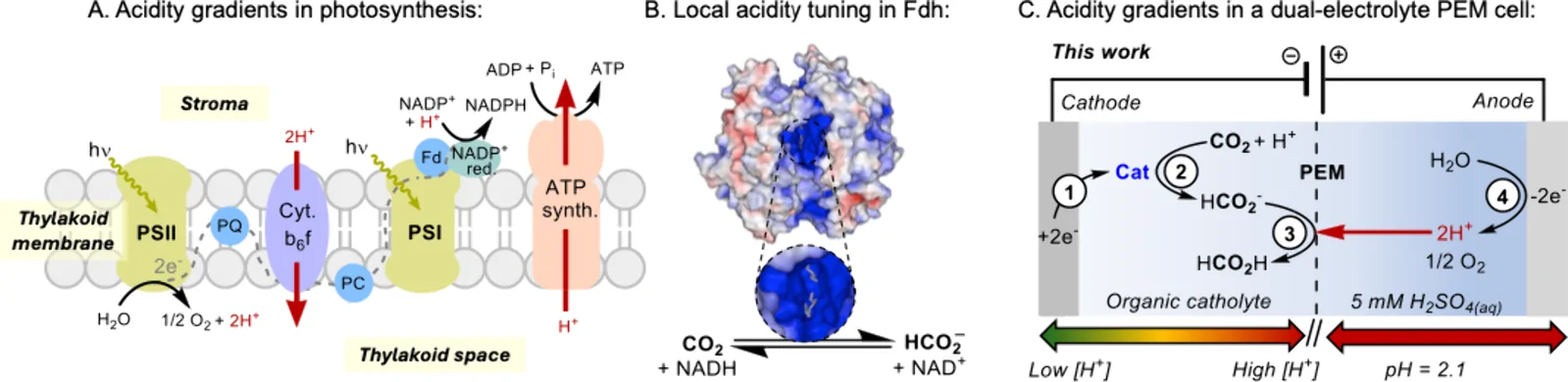
A) Schematic representation of the light-dependent machinery of natural photosynthesis. B) Electrostatic potential surface (calculated with PyMOL’s APBS plugin) and active site pocket of the W-Fdh from *Desulfovibrio vulgaris* (DvFdhAB, PDB 6SDR). Electropositive, electronegative and neutral regions are colored in blue, red and white, respectively. C) Representation of the acidity gradient in a dual-electrolyte proton-exchange membrane (PEM) cell.

Inspired by the biological approach that couples proton transfer across membranes to manage acidity gradients, we report a generalizable strategy for generating formic acid (rather than formate) during CO_2_ electroreduction that leverages pH gradients across a proton exchange membrane (PEM) in a dual-electrolyte electrochemical cell. Unlike conventional PEM electrolyzers or heterogeneous local-pH engineering strategies,^33^ this system uses a freely diffusing molecular electrocatalyst in a neutral organic catholyte, such that the acidity gradient is established by proton transport from the PEM rather than by rapid proton depletion at the cathode. Multiscale modeling shows that water oxidation at the anode generates a proton gradient far from the electrode surface that enables electrochemical CO_2_R to HCO_2_H while mitigating H_2_ evolution (Figure 1C). A further benefit of the approach is that water is the source of protons for CO_2_ reduction.

Our studies began with an iridium catalyst that can reduce CO_2_ to HCO_2_^–^ with good selectivity in a range of solvents.^34,35^ We recently demonstrated that [Cp*Ir­(bpy)­Cl]^+^ (**[Ir–Cl]** ^**+**^ as a chloride salt, Cp* = pentamethylcyclopentadienyl, bpy = 2,2'-bipyridine) catalyzes CO_2_ reduction to HCO_2_^–^ (ca. 50% FE_HCO2–_) on a reticulated vitreous carbon (RVC) electrode at –2.1 V vs Fc^+/0^ in 2-propanol (IPA) 0.1 M [nBu_4_N]­[OTf] electrolyte, using a classical H-cell with a porous glass frit separator.^36^ In initial studies, we used the same catalyst **[Ir–Cl]** ^**+**^ and the same IPA/[nBu_4_N]­[OTf] catholyte, while changing the anolyte to 5 mM H_2_SO_4(aq)_ and replacing the glass frit with a Nafion 117 PEM separator. With vigorous stirring during the electrolysis, **[Ir–Cl]** ^**+**^ produced 1.8 mM HCO_2_H/HCO_2_^–^ (mole fraction of formic acid, χ_*HCO2H*_ = 0.35) in only 10% FE (Figures S14 and S15). The same experiment was then attempted without stirring during electrolysis, to preserve any acidity gradients. After the electrolysis, the catholyte was stirred to prepare a sample for NMR spectroscopy that showed that presence of pure formic acid (χ_*HCO2H*_ = 1.0, 7.3 mM HCO_2_H), produced with a Faradaic efficiency of 47% that reflects the inherent selectivity of the catalyst (Figure 2A). In addition, small amounts (6% FE) of isopropyl formate (HCO_2_^*i*^Pr) were detected in the unstirred electrolysis, whereas in the other conditions this product was not observed. The protonation state of the product in the reaction mixture was assigned by a ^1^H NMR assay monitoring the chemical shift of the formyl signals of HCO_2_H/HCO_2_^–^ (δ_obs_). Figure 2B shows a chemical shift calibration curve obtained by titrating HCO_2_H in isopropanol (IPA) with 2-*tert*-butyl-1,1,3,3-tetramethylguanidine (^t^BuTMG). The signal for HCO_2_H (δ_HCO_2_H_ = 7.99 ppm) broadened and shifted downfield, ultimately reaching the chemical shift of HCO_2_^–^ (δ_HCO_2_^–^_ = 8.52 ppm).^27^

**Figure 2:**
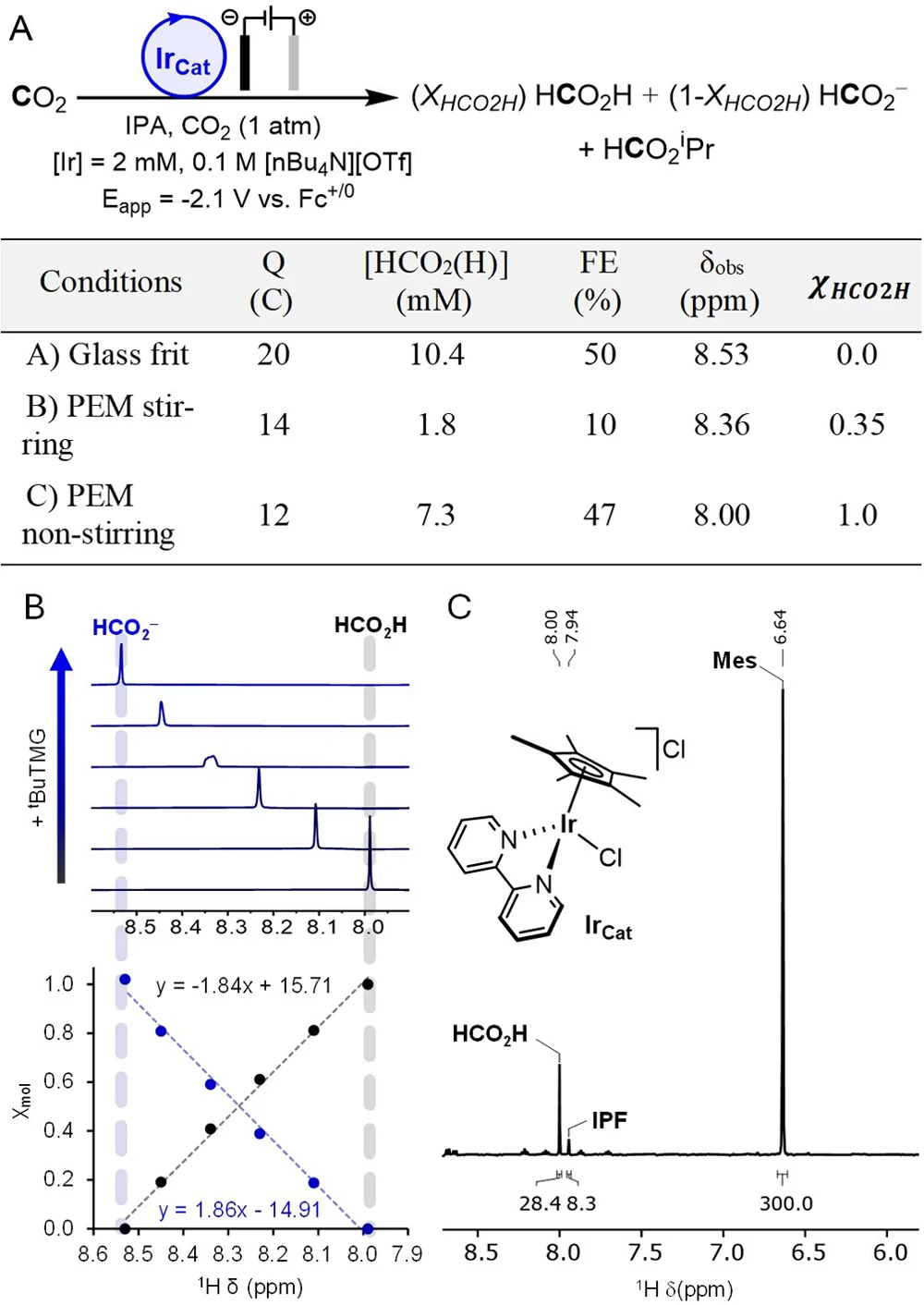
A) Scheme showing electrolysis conditions for **[Ir–Cl]** ^**+**^ along with table of CPE results in different conditions. See SI Section S2 for details. B) ^1^H NMR titration of a HCO_2_H (12 mM) solution in IPA with ^t^BuTMG with calibration curves for χ_*HCO*2*H*_ (black) and χ_*HCO*2–_ (blue). C) ^1^H NMR spectrum of the electrolyzed solution in a PEM cell without stirring. Molecular structure of **[Ir–Cl]** ^**+**^ (**Ir**_**Cat**_). See SI Section 3 for full details.

The formation of HCO_2_H indicates that protons generated by the oxygen evolution reaction (OER) at the Ti anode successfully passed across the PEM to drive CO_2_ reduction. The presence of HCO_2_^*i*^Pr is also consistent with buildup of strong acid in the catholyte because Fischer esterification of HCO_2_H in IPA at room temperature requires an acid such as trifluoromethanesulfonic acid (TfOH) or protonated Nafion, which also contains –CF_2_SO_3_H functional groups.^36,37^ The aqueous anolyte maintained a constant pH during electrolysis (pH = 2.16 before CPE and 2.14 after CPE), consistent with charge balance maintained by proton transport through the PEM. Analysis of the catholyte after 2 h of unstirred electrolysis revealed the presence of HCO_2_H in samples collected both near the cathode and near the membrane (Table S4). Less than 5% of HCO_2_H/HCO_2_^–^ was detected in the anolyte after 16 h of CPE, with model studies suggesting that crossover of neutral HCO_2_H is minimal under our electrolysis conditions (see SI Section 4 for details).

Isotopic labeling studies confirmed the origin of the product and quantified carbonates formed during the reaction. Electrolysis in the dual-electrolyte PEM cell, but under ^13^CO_2_ atmosphere, produced H^13^CO_2_H (^1^H δ = 8.00, doublet, ^2^ *J*_C–H_ = 224 Hz, ^13^C δ = 163.7), with no evidence for carbonate salts. In contrast, electrolysis using a glass frit (Table 1A conditions) produced H^13^CO_2_^–^ (δ = 8.54, doublet, ^2^ *J*_C–H_ = 185 Hz) as well as isopropyl carbonate (^*i*^PrOCO_2_^–^, ^13^C δ = 159). The dual-electrolyte approach therefore holds promise for both sourcing protons in CO_2_ reduction from water and improving carbon efficiency by preventing buildup of carbonate salts during CO_2_R.

The successful generation of formic acid with good selectivity in the IPA/H_2_O dual-electrolyte PEM cell prompted us to examine acetonitrile-based catholytes as a way of testing the generality of the formic acid synthesis under conditions where well-defined thermodynamic parameters are available (See SI Section 5 for details). Furthermore, in acetonitrile it would not be possible to form alkyl formates as a minor byproduct. In acetonitrile containing 0.1 M [nBu_4_N]­[PF_6_] and 1% v/v H_2_O, **[Ir–Cl]** ^**+**^ mainly produced HCO_2_^–^ from CO_2_R with 42 ± 2% FE after CPE at −2.0 V in a glass-frit-separated H-cell (uncertainty is the standard deviation of two replicates). Yet, CPE in the dual-electrolyte PEM cell produced HCO_2_H as the major CO_2_R product with 53 ± 8% FE and χ_*HCO*2*H*_ = 0.97 as determined from the formyl signal chemical shift at 8.07–8.08 ppm. Stirred PEM cell conditions decreased the CO_2_ reduction efficiency (FE_HCO2H/HCO2–_ = 6%, Table S5), further suggesting that controlling proton gradients is critical.

The developing proton gradients can be visually observed during electrolysis. While the catholyte solution is pale yellow before CPE, an increasingly large volume around the cathode surface turned dark purple and the solution adjacent to the membrane turned golden yellow under nonstirred conditions in MeCN with 1% H_2_O under N_2_ (Figure 3A). This is in line a concentration gradient developing across the cell, with accumulation of purple, doubly reduced Cp*Ir­(bpy) (**[Ir]** ^**0**^) near the cathode and golden **[Ir–H]** ^**+**^ near the membrane. Thin-layer UV–vis spectroelectrochemistry in MeCN (1% H_2_O) corroborates the formation of purple **[Ir]** ^**0**^ near the cathode under N_2_ (Figure 3B).

**Figure 3:**
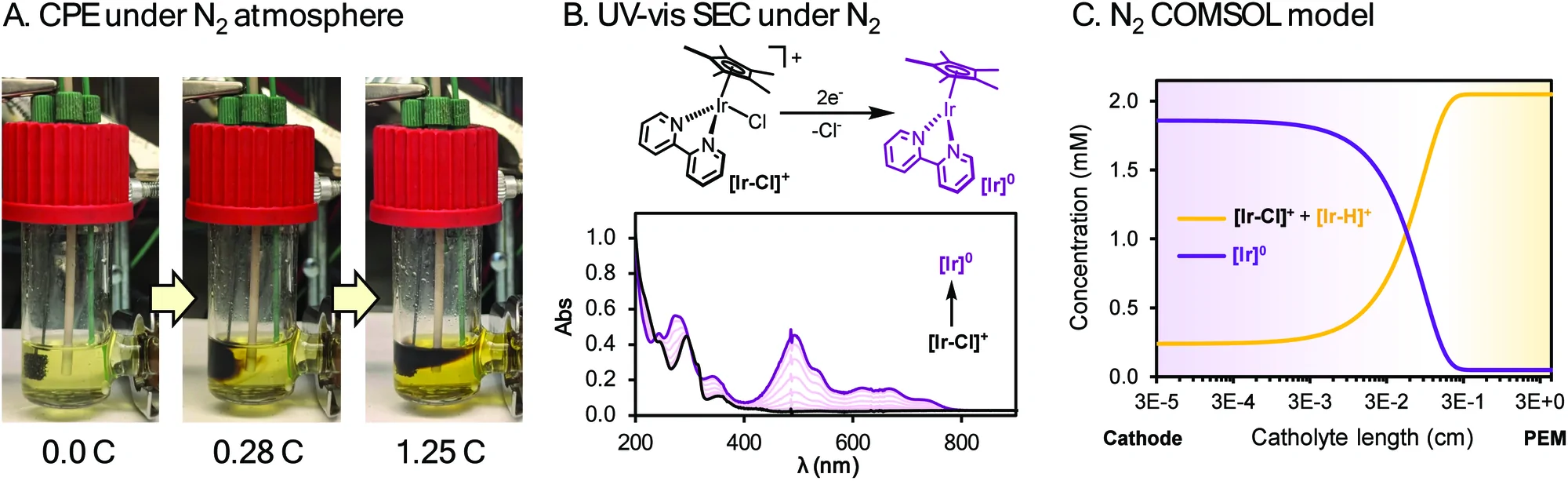
A) Nonstirred PEM setup during CPE at −2.0 V vs Fc^+/0^ under N_2_ atmosphere from the beginning of the experiment (0.0 C) to longer times where more charge had passed (0.28 and 1.25 C). Conditions: **[Ir–Cl]** ^**+**^ (2 mM) in MeCN 1% H_2_O 0.1 M [nBu_4_N]­[PF_6_], with RVC cathode separated from the anodic compartment (5 mM H_2_SO_4_ in H_2_O over Ti foil) by a Nafion 117 PEM in the absence of CO_2_. B) UV–vis Spectroelectrochemistry (SEC) under N_2_ atmosphere during a linear sweep voltammogram from –0.5 V to –1.3 V vs Ag wire pseudoreference. Conditions: **[Ir–Cl]** ^**+**^ (2 mM) in MeCN 1% H_2_O 0.1 M [nBu_4_N]­[PF_6_], with Au microgrid cathode, Pt counter electrode, and Ag wire pseudoreference electrode. C) COMSOL simulated concentration profile of **[Ir]** ^**0**^ (purple) and **[Ir–H]** ^**+**^ (yellow) along the cathode-PEM axis at 120 min.

Multiscale kinetic modeling is a powerful tool for understanding and visualizing the concentration gradients that develop under electrocatalysis conditions.^38,39^ COMSOL is routinely applied to heterogeneous electrocatalysis.^40,41^ A key distinction is that in homogeneous electrolysis reactants and products diffuse freely in solution, and catalyst speciation evolves dynamically in both time and space. To capture these effects, we developed a time-dependent one-dimensional model that simulates the electrochemical environment across a representative H-cell (Figure S28). The model considers the plane connecting the cathode and anode through the PEM, enabling simulation of species transport (in the absence of stirring) and reaction profiles throughout the cathode-membrane axis (see SI Section 7 for details). Charge balance is achieved by H^+^ transfer through the PEM. To capture the reactivity under N_2_, the model includes the 2e^–^ reduction of **[Ir–Cl]** ^**+**^ to **[Ir]** ^**0**^, its protonation to give **[Ir–H]** ^**+**^ and a 2e^–^/1H^+^ reduction process evolving H_2_ while regenerating **[Ir]** ^**0**^ (eq S30–S32). Proton transport in the catholyte is modeled as occurring through HCl, due to Cl^–^ from the catalyst being the strongest base in the system under N_2_ (p*K*_a_(HCl) = 10.3 in MeCN).^42^ The total chloride concentration is 4.0 mM, arising from 2.0 mM **[Ir–Cl]**[Cl], which contributes one chloride ligand and one chloride counterion per Ir. Figure 3c shows the **[Ir]** ^**0**^, [**Ir–H**]^+^, and **[Ir–Cl]** ^**+**^ concentration profiles along the cathode-PEM axis (3.5 cm distance). At early times, **[Ir]** ^**0**^ accumulates near the cathode, extending around 3 mm away from the surface. Protonation does not occur due to the low local concentration of HCl. Protons leave the PEM convert to HCl and diffuse away from the membrane and encounter **[Ir]** ^**0**^ diffusing away from the cathode, generating **[Ir–H]** ^**+**^ and **[Ir–Cl]** ^**+**^. H_2_ formation from **[Ir–H]** ^**+**^ can only occur if a local acid of p*K*_a_ ≤ 18 (in MeCN) is present to protonate the hydride intermediate (see thermochemical analysis in Figure S27). The spectroscopy and modeling together indicate that the region near the electrode maintains an acidity similar to the initial solution, while the region near the membrane becomes more acidic. The experimental observation of hydride formation confirms that proton transport through the cell is effective; if proton flow were limited, the entire catholyte would turn purple due to accumulation of **[Ir]** ^**0**^.

Analogous studies of the proton gradient were also carried out under CO_2_. Under electrocatalytic conditions in the PEM cell (both in MeCN with 1% H_2_O and in IPA), **[Ir]** ^**0**^ does not accumulate visibly near the cathode. Spectroelectrochemistry in CO_2_-saturated MeCN with 1% H_2_O shows rapid conversion of **[Ir–Cl]** ^**+**^ to **[Ir–H]** ^**+**^ near the cathode surface, identifying the key iridium hydride intermediate as the resting state under catalytic conditions (Figure 4A). The change in the Ir species moving from N_2_ to CO_2_ occurs because the bulk solution is rendered more acidic by CO_2_ reacting with H_2_O to generate hydrogen carbonate. The apparent p*K*_a_ (CO_2_ + H_2_O) = 23.4 in MeCN (c.f. H_2_O in MeCN, p*K*_a_ > 30 under N_2_),^43^ matches well the **[Ir–H]** ^**+**^ p*K*_a_ (23.3 in MeCN).^44^ CPE in the presence of the indicating dye bromocresol green further support the formation of an acidity gradient under nonstirred CO_2_R conditions. After initial basification, the blue-colored catholyte turned yellow near the Nafion membrane as the electrolysis proceeded (Figure S13). This observation is consistent with acidification beginning at the membrane–catholyte interface and then extending into the catholyte.

A more general COMSOL model was developed for the reactions under CO_2_, to focus on the acidity gradients across the cell. A species HX was introduced to represent the acidity of the CO_2_/H_2_O/MeCN mixture, p*K*_a_(HX) = 23.4. The other proton carrier present in the model is HCO_2_^–^ (p*K*_a_(HCO_2_H) = 20.9 in MeCN), generated after the first catalytic turnover.^45^ The CO_2_ atmosphere model captures the general features of the experimental observations: under CO_2_, high concentrations of HCO_2_^–^ are initially generated near the cathode and high concentrations of H^+^ are generated near the PEM (Figure 4B). This is distinct from heterogeneous electrocatalysis where the acidity gradient at the electrode surface is most commonly discussed;^17,33^ with freely diffusing molecular catalysts operating at relatively low current density in a buffered medium, the acidity near the electrode is not changing substantially compared to the change near the membrane. The present model shows that HCO_2_^–^ and H^+^ neutralize each other in the middle part of the cell as the electrolysis continues, preventing acidification and loss of selectivity near the cathode (Figure 5A). Surface acid sites on the Nafion can contribute additional protons to the catholyte (ca. 5 mM, see Figure S12), however, so if stirring is introduced the acid would reach the cathode and selectivity would erode, as observed experimentally. In an unstirred electrolysis, a formate-rich region and an acidic region initially build up at opposite ends of the compartment, and neutralize in the middle of the cell. A sensitivity analysis shows that varying the cell length, diffusion coefficients, and time can significantly impact the precise region where HCO_2_H forms during electrolysis (Figure S30).

**Figure 4:**
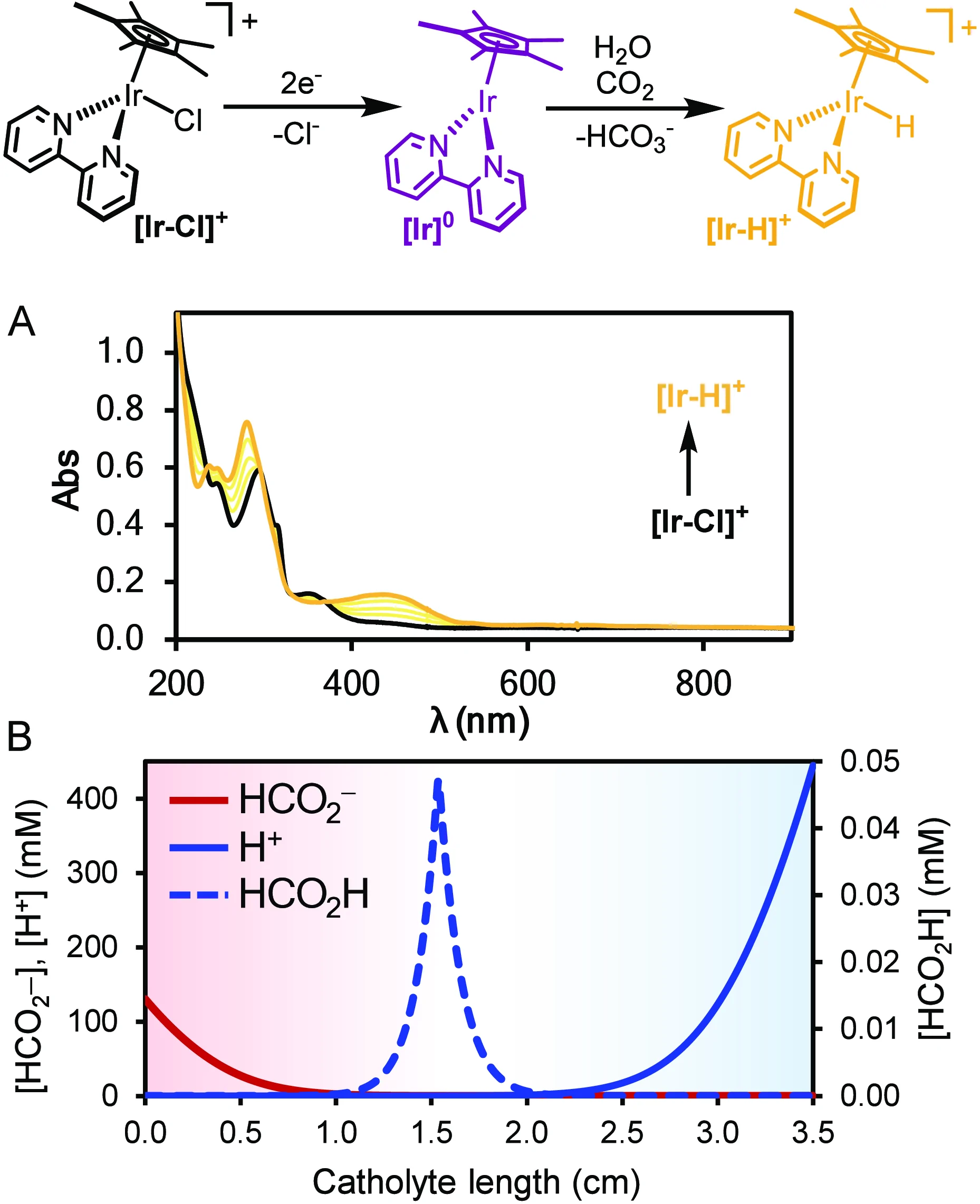
Reactivity under CO_2_. A) UV–vis SEC under CO_2_ with starting spectra in in black and yellow, respectively. B) COMSOL simulated concentration profiles of HCO_2_^–^ (solid red), H^+^ (solid blue), HCO_2_H (dashed blue) along the Cathode-PEM axis at 120 min. Integration of the HCO_2_^–^ and HCO_2_H concentration profiles and normalization to the total catholyte volume (4 mL) yields a total HCO_2_^–^ + HCO_2_H concentration of 3.9 mM.

**Figure 5:**
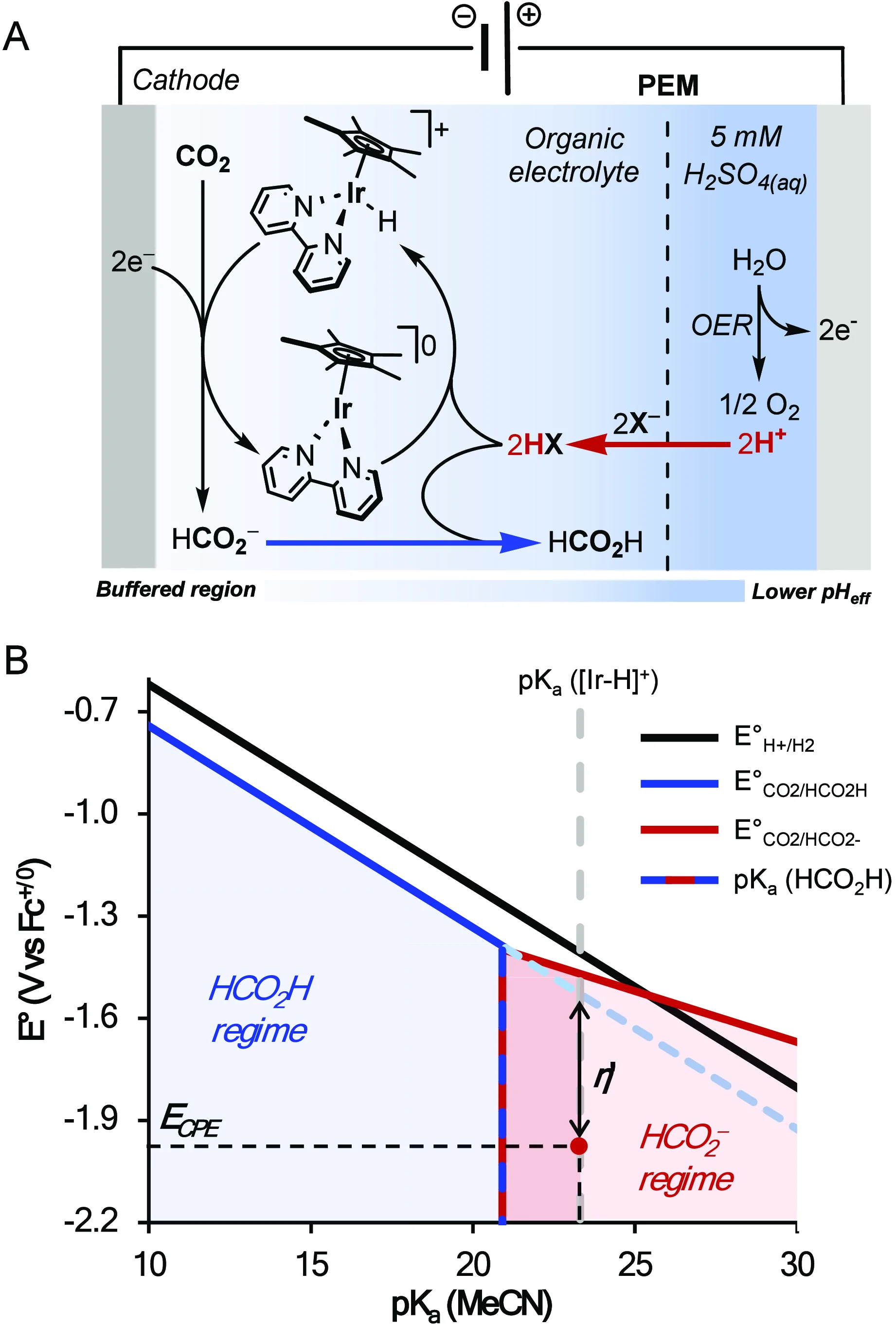
A) Simplified mechanistic proposal. B) Thermodynamic potential-p*K*_a_ diagram for the electrochemical HER (black) and CO_2_R to HCO_2_^–^ (red) and HCO_2_H (blue) in MeCN.

Pourbaix diagrams,^46^ which portray the thermodynamically favored species as a function of reduction potential and acidity, can help explain the selectivity in CO_2_ reduction. Experiments and COMSOL models suggest that the proton carriers near the cathode surface have a p*K*_a_ of 23.4 (*vide supra*), sufficient to protonate **[Ir]** ^**0**^ and drive catalysis — but insufficient to protonate HCO_2_^–^, which therefore builds up near the cathode. Figure 5B teaches us that, under these initial bulk solution acidity conditions, the electrochemical overpotential for HCO_2_^–^ generation (η) would be 0.51 V.^47^ The observed product of electrolysis is not HCO_2_^–^, however, but HCO_2_H. The overpotential to produce HCO_2_H in a medium with p*K*_a_ = 23.4 is different, η’ = 0.44 V (Figure 5B). The cell design therefore results in an apparent 70 mV reduction in overpotential. As in biology, proton translocation across the membrane contributes to the overall thermodynamics of the process, creating the proton-motive force that is used to power enzyme catalysis.^48,49^

With a deeper understanding of the acidity gradients, we assessed the generality of our approach with two other electrocatalysts (Chart 1) at the same applied potential used for [Ir–Cl], selected based on comparison of their cyclic voltammograms (Figure S24). First, we tested a cobalt catalyst with pendent amines, [CpCo­(P_2_N_2_)­(NCCH_3_)]­[PF_6_]_2_ (**Co**_**Cat**_) that was reported to reduce CO_2_ to HCO_2_^–^ in N,N'-dimethylformamide.^50^ In a glass frit H-cell, **Co**_**Cat**_ was electrolyzed at −2.0 V under our standard conditions of CO_2_-saturated MeCN containing 0.1 M [nBu_4_N]­[PF_6_] and 1% H_2_O, resulting in 30% FE for HCO_2_^–^. The presence of H_2_ was also confirmed by gas chromatography (GC) analysis of the headspace. As in the case of **[Ir–Cl]** ^**+**^, the dual-electrolyte PEM setup led to the electrochemical CO_2_R to HCO_2_H with a similar 29% FE (χ_*HCO*2*H*_ = 0.96) for **Co**_**Cat**_. This shows dual-electrolyte proton gradient approach can be applied to a first-row transition metal catalyst system containing proton relays with the same effect of maintaining the carbon-product selectivity while the product distribution can be shifted toward formate/formic acid.

**Chart 1:**
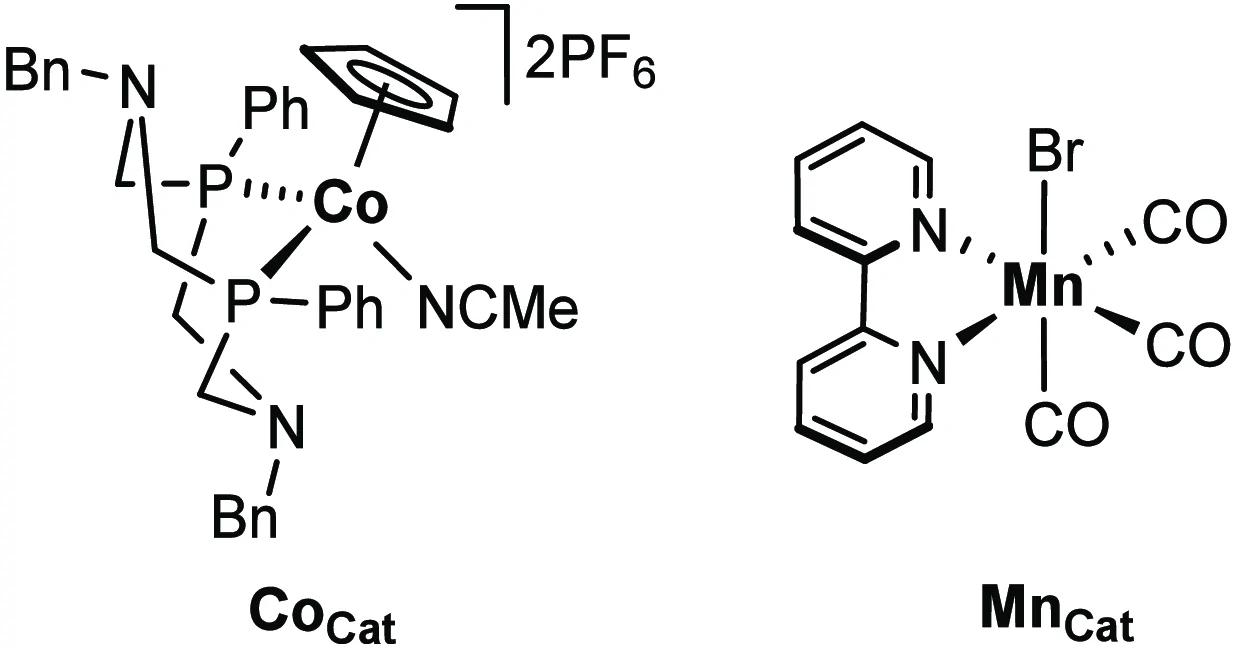
Selected CO_2_R Electrocatalysts

Then, the Mn catalyst Mn­(bpy)­(CO)_3_Br (**Mn**_**Cat**_) was chosen because it is a candidate for selectivity switching from CO to formic acid. **Mn**_**cat**_ was first reported to selectively reduce CO_2_ to CO in MeCN,^51^ but in the presence of amines and alcohols the product distribution can be shifted toward formate.^52–54^ When **Mn**_**Cat**_ was used as catalyst in MeCN electrolyte with added 2,2,2-trifluoroethanol (1.5% v/v), CO was the major CO_2_R product (93% FE_CO_ and 4% FE_HCO2–_). Using the dual-electrolyte PEM cell, the carbon-containing product selectivity shifted dramatically, as the total HCO_2_H/HCO_2_^–^ Faradaic efficiency *increased* 6-fold from 4% to 24%. As expected, the protonation state also shifted from pure HCO_2_^–^ with a frit separator to HCO_2_H (χ_*HCO*2*H*_ = 0.96) in the PEM cell. For the Mn catalyst, the controlled transfer of H^+^ from the anodic water oxidation through the PEM alters the selectivity from CO to HCO_2_H. The shift in carbon product selectivity could be attributed to the higher p*K*_a_ of the [Mn–H] intermediate compared to the Ir and Co systems (Table S7) and suggests that acidic species reach close enough to the cathode to influence the catalytic selectivity.

In summary, a strategy was introduced for selective CO_2_R to HCO_2_H using molecular electrocatalysts in a dual-electrolyte PEM cell. Protons from anodic water oxidation migrate through the PEM into an organic catholyte, spatially separating the high-acid region from the electrode surface, establishing an acidity gradient along the cathode-PEM axis. Under unstirred conditions, this pH gradient affords HCO_2_H in ca. 50% FE with **[Ir–Cl]** ^+^, with no unwanted carbonate salts detected. A time-dependent COMSOL model, supported by product crossover, spectroscopic, and thermodynamic analyses, captures the spatiotemporal dynamics of acidity gradients that succeed in suppressing HER at the electrode while producing HCO_2_H upon mixing. This behavior extends to other CO_2_R catalysts in protic MeCN, which deliver significant HCO_2_H in mixed organic/aqueous PEM setups even when CO or formate were previously dominant. This study highlights the power of macroscopic pH gradients at mitigating parasitic HER and carbonate formation, guiding the design of CO_2_ electrolyzers, including flow-cell configurations in which controlled flow rates could enable more quantitative regulation of the acidity gradient.

## Supplementary Material



## Notes

Full experimental details, characterization data, and modeling parameters (PDF)
