# Supplementary material for: Controlled Acidity Gradients Enable CO2 Reduction to Formic Acid (Not Formate) by Molecular Electrocatalysts
Source: ACS Cent Sci. 2026 Jul 17;12(8):1158–64. doi: 10.1021/acscentsci.6c00571 (PMC13523672; doi:10.1021/acscentsci.6c00571)
Supplement: Supplementary file 1 [file oc6c00571_si_001.pdf]

Supporting Information

for

Controlled Acidity Gradients Enable CO_2 Reduction to Formic Acid (Not Formate) by Molecular Electrocatalysts

Sergio Fernández,[1] Pablo Fernandez,[1] Amanda I. Arnoff,[2] Elizabeth A. Scholer,[1] Charlotte L. Montgomery,[1] Jillian L. Dempsey,[1] Thomas E. Mallouk,*,[3] René Lopez,*,[2] Alexander J. M. Miller*,[1]

[1]Department of Chemistry, University of North Carolina at Chapel Hill, Chapel Hill, North Carolina 27599-3290, United States
[2]Department of Physics and Astronomy, University of North Carolina at Chapel Hill, Chapel Hill, North Carolina 27599, United States
[3]University of Pennsylvania, Department of Chemistry, 231 S 34th St, Philadelphia, Pennsylvania 19104, United States

*Corresponding Author E-mail Addresses:
A.J.M.M.: ajmm@email.unc.edu
R.L.: rln@physics.unc.edu
T.E.M.: mallouk@sas.upenn.edu

Table of Contents

1. General Methods and Materials S2
2. Electrochemical Studies S3
3. Product quantification S5
4. Product crossover evaluation S8
5. Controlled potential electrolysis S16
6. Experimental study of the acidity gradients S24
7. COMSOL Multi-scale Modeling S26
7.1. Methodology S26
7.2. Parameters for COMSOL model S31
7.3. COMSOL simulation results S33
8. References S35

1. General Methods and Materials

All operations were carried out using standard Schlenk techniques under an atmosphere of nitrogen or in a nitrogen-filled glovebox, unless otherwise stated. 2-Propanol (^{i}PrOH or IPA) obtained from VWR (HPLC grade) and mesitylene (Mes) obtained from Sigma Aldrich were dried over 3 Å molecular sieves, sparged with N_2 and stored in a nitrogen-filled glovebox. Acetonitrile was dried and deoxygenated using a Pure Process Technology solvent system and stored over 3 Å molecular sieves. Deuterated solvents were purchased from Cambridge Isotopes Laboratories, Inc. Nafion 117 membrane was obtained from IonPower and unless otherwise indicated the sheet was, prior to use, cut into approximately 1.5 by 1.5 cm square sheets, soaked in 1 M HCl overnight, and stored in 5 mM H_2SO_4. High purity carbon dioxide (99.995%) was obtained from Airgas and used as received. Tetra-*n*-butylammonium trifluoromethanesulfonate (triflate) [TBA][OTf] or hexafluorophosphate [TBA][PF_6], were used as a supporting electrolyte for electrochemical reactions in IPA and acetonitrile, respectively. Both were obtained from Sigma Aldrich and then recrystallized in a saturated solution of dichloromethane and diethyl ether at 0 °C or through a hot extraction in IPA and a crystallization at –18 °C. The crystalline material was washed with diethyl ether and dried in vacuo for >18 hours. Ferrocene (Fc) was obtained from Sigma Aldrich and purified by recrystallization from a saturated solution of pentane at –18 °C overnight. The Fc crystals were isolated via filtration, washed with cold pentane, and dried in vacuo for >18 hours. Purified electrolyte salts and Fc were stored in a nitrogen-filled glovebox. [Cp*Ir(bpy)Cl][Cl] (**Ir$_{Cat}$**),[1] [Mn(bpy)$(CO)_3$Br] (**Mn$_{Cat}$**),[2] [Co($P^{Ph}{}_2N^{Bn}{}_2$)(MeCN)][PF_6] (**Co$_{Cat}$**)[3] and tetrabutylammonium formate[4] were synthesized according to literature procedures. For the synthesis of **Ir$_{Cat}$**, CH_2Cl_2 was used instead of MeOH as solvent to avoid accidental contamination or false positives for CO_2 reduction studies. All other chemicals were commercially available and used as received, unless otherwise noted in the synthetic procedure.

2. Electrochemical Studies

Electrochemistry experiments were performed using a WaveDriver bipotentiostat controlled with Aftermath software (Pine Research), a CH Instruments potentiostat workstation (model CHI 760E), or a Metrohm Autolab Potentiostat (PGSTAT204).

Cyclic voltammetry (CV) experiments were performed in a one-compartment cell using a three-electrode system: a glassy carbon (3 mm diameter) working electrode, a Pt wire counter electrode, and a Ag wire pseudo-reference electrode. Ferrocene was added at the beginning of the CV experiment as an internal standard to calibrate the pseudo-reference, and therefore, all the potentials are given *versus* the ferrocenium/ferrocene ($Fc^{+/0}$) couple.

Spectroelectrochemistry experiments were conducted on an optically transparent thin-layer electrode (OTTLE) cell, equipped with Au working and Pt auxiliary minigrid electrodes, an Ag microwire pseudo-reference electrode and CaF_2 windows.[5] Experiments were carried out at 2 mM **Ir$_{Cat}$** concentration in MeCN with 1% H_2O. The same electrolyte solution (without Ir complex) was used for solvent subtractions. UV-vis spectra were measured with an Agilent 8453 diode array spectrophotometer (λ = 190–1100 nm).

Controlled potential electrolysis (CPE) experiments were performed using two different electrochemical cells: (i) a conventional frit-separated H-cell (fine sintered-glass frit; Figure S1) and (ii) a two-compartment H-cell incorporating O-ring joints for PEM assembly (Figure S2). In both cells, CPE experiments used a high surface area reticulated vitreous carbon (60 ppi RVC from Duocel®) working electrode.

In cell (i), a Pt coil counter-electrode, and an Ag wire pseudo-reference electrode were employed. A reference electrode capillary utilizing a CoralPor frit was filled with the same electrolyte solution. The cell was charged with 2 mM catalyst in an electrolyte solution in the working compartment. The counter compartment contained 50 mM of Fc in the same electrolyte solution. In both compartments, 20 mM Mes was present as an internal standard for both NMR and GC analysis. The cell was then sealed and a constant potential of –2.07 V vs $Fc^{+/0}$ was applied while stirring at 1000 rpm.

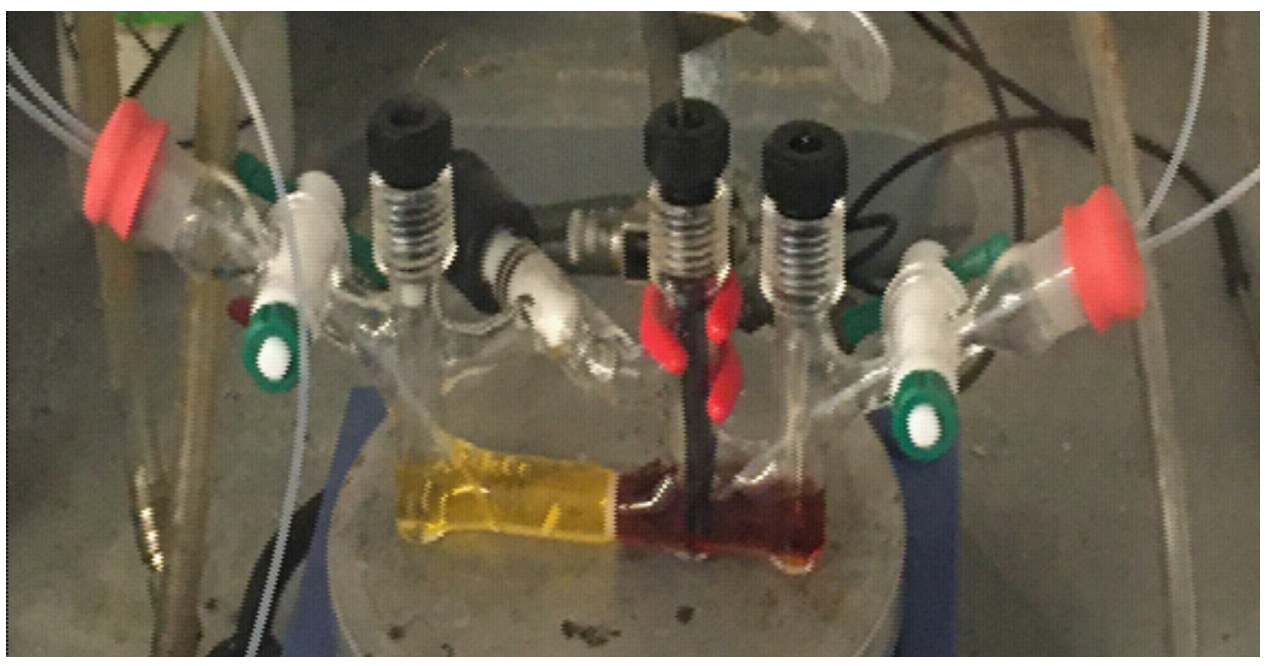

Figure S1. Photograph of the frit-divided H-cell (cell i) in which CPE experiments were performed. The working electrode and reference electrode are placed on the right-hand side, and the counter electrode is placed on the left-hand side of the cell. During electrolysis, the pressure bridge was kept open to equilibrate the pressure in both compartments over time, and the stopcocks, Kontes valve and Ace-7 glass joints provide a gas-tight seal.

In cell (ii), a Ti foil counter-electrode, and a leakless Ag/AgCl reference electrode (from eDAQ) were employed. The working compartment was charged with a 2 mM solution of the catalyst in the organic electrolyte mixture containing 20 mM Mes as an internal standard. The counter compartment was filled with a solution of 5 mM H_2SO_4 in water.

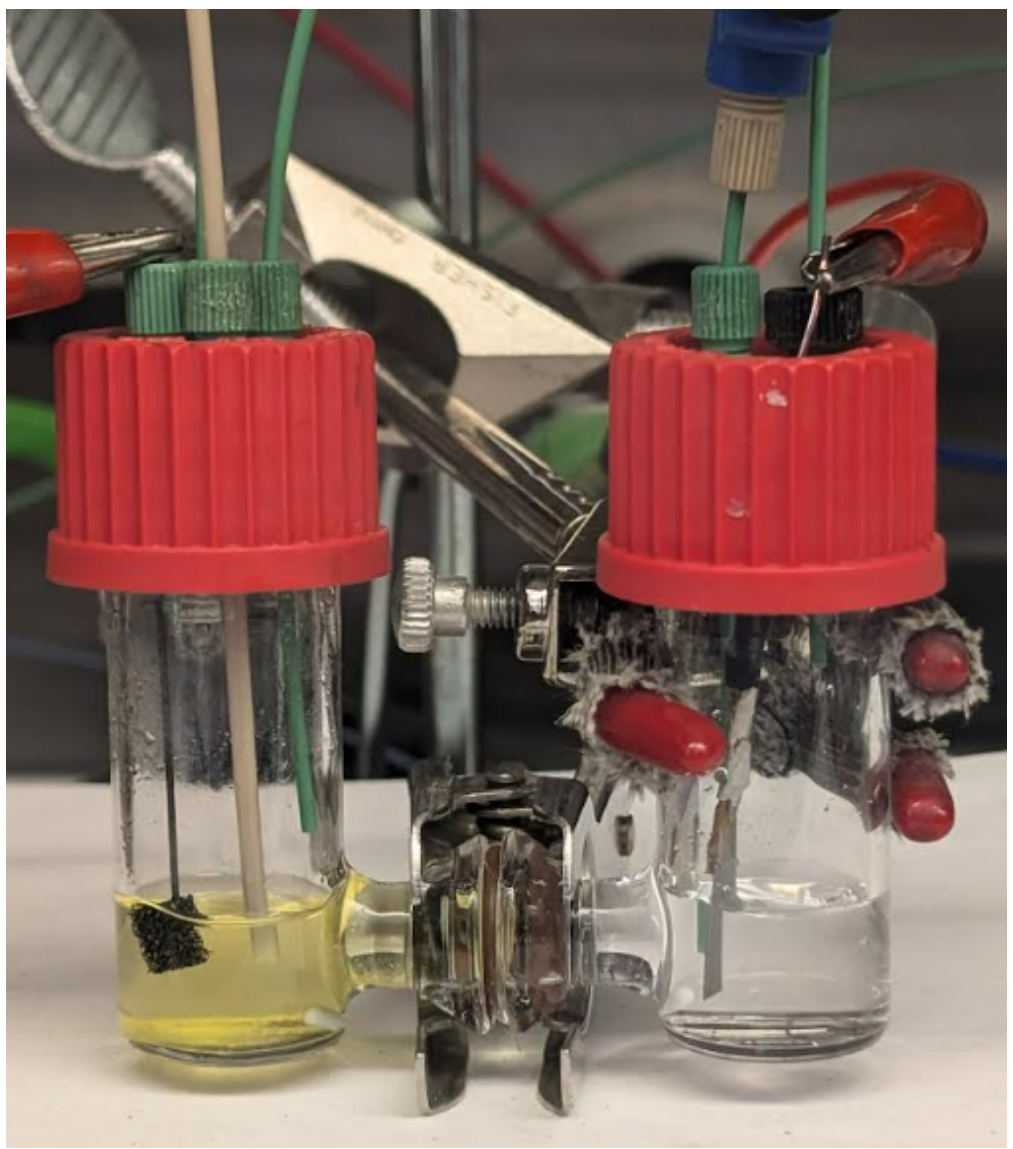

Figure S2. Photograph of the PEM cell (cell ii) in which CPE experiments were performed. The working electrode (RVC) and reference electrode (leakless Ag/AgCl) are placed on the right-hand side, and the counter electrode (Ti foil) is placed on the left-hand side of the cell. During electrolysis, the pressure bridge was either kept open, or the electrolysis was performed under positive CO_2 flow to equilibrate the pressure in both compartments over time.

In both cells, the solutions were sparged thoroughly with CO_2 for at least 20 minutes and the electrolysis potential was referenced to $Fc^{+/0}$ by adding a small amount of Fc at the beginning of the experiment or by external calibration of the (pseudo)reference measuring $Fc^{+/0}$ at a glassy carbon disk before/after CPE.

3. Product quantification

Analysis of liquid products from CPE was performed using ^{1}H NMR spectroscopy. NMR spectra were collected on either a 400 MHz or 600 MHz spectrometer at 298 K, unless otherwise noted. THF-d_8 and MeCN-d_3 obtained from Cambridge Isotope Laboratories Inc. were used as received from the supplier in a 10%$_{v/v}$ with respect to the protio solvent. For ^{1}H NMR quantification purposes, all spectra were acquired in the presence of Mes (20 mM) or as an internal standard with a delay time of 10 seconds. ^{1}H and ^{13}C chemical shifts are reported relative to TMS based using the internal standard chemical shift for reference.

For the NMR sample preparation, the cell was opened and a 450 µL aliquot was obtained. A 10 %$_{v/v}$ of THF-d_8 (50 µL) was added to the electrolysis sample to aid in NMR acquisition and a ^{1}H NMR (delay time D1 = 10 s, 16 scans) spectrum was obtained. Then, the formate and Mes signals at 8.54 ppm (1H) and 6.66 ppm (3H), respectively, were carefully integrated. Since the concentration of Mes is known (20 mM) and it does not change during the CPE experiment, we can use it as our internal standard. In cases where the formyl signal overlapped with other resonances, the absolute integrals of both the formyl and Mes signals were determined by numerical fitting. Then, the peak area of Mes can be set to 3 (since the aromatic signal of Mes corresponds to 3 equivalent H) and the concentration (in mM) of formate generated from CPE can be calculated according to the following equation:

$$[Product] = \frac{[Mes] \cdot Area\ Product/1}{(Area\ Mes/3)} \quad (S1)$$

From the total charge passed (Q) and the mol of electrons (n_{e-}) consumed per mol of product ($n_{product}$) generated from CO_2 reduction, one can calculate the Faradaic efficiency (FE) for that product. For the specific case of HCO_2^-/HCO_2H n_{e-} = 2n (as for H_2 and any other 2e$^-$ reduction product).

$$FE(\%) = \frac{n_{e-} \cdot n_{product} \cdot F}{Q} \cdot 100 = \frac{2 \cdot n_{product} \cdot F}{Q} \cdot 100 \quad (S2)$$

where $n_{product}$ is the number of moles of formate/formic acid produced and F = 96485 C/mol.

To assess the mole fraction of HCO_2H, χ_{HCO2H}, after CPE, an NMR assay was developed in which the ^{1}H NMR chemical shift of the formyl proton was calibrated against known HCO_2H/HCO_2^- molar fractions. For this purpose, NMR titration experiments were performed in two solvent systems: dry isopropanol (IPA, Fig. S3A) and acetonitrile (MeCN) containing 1% H_2O (Fig. S3B).

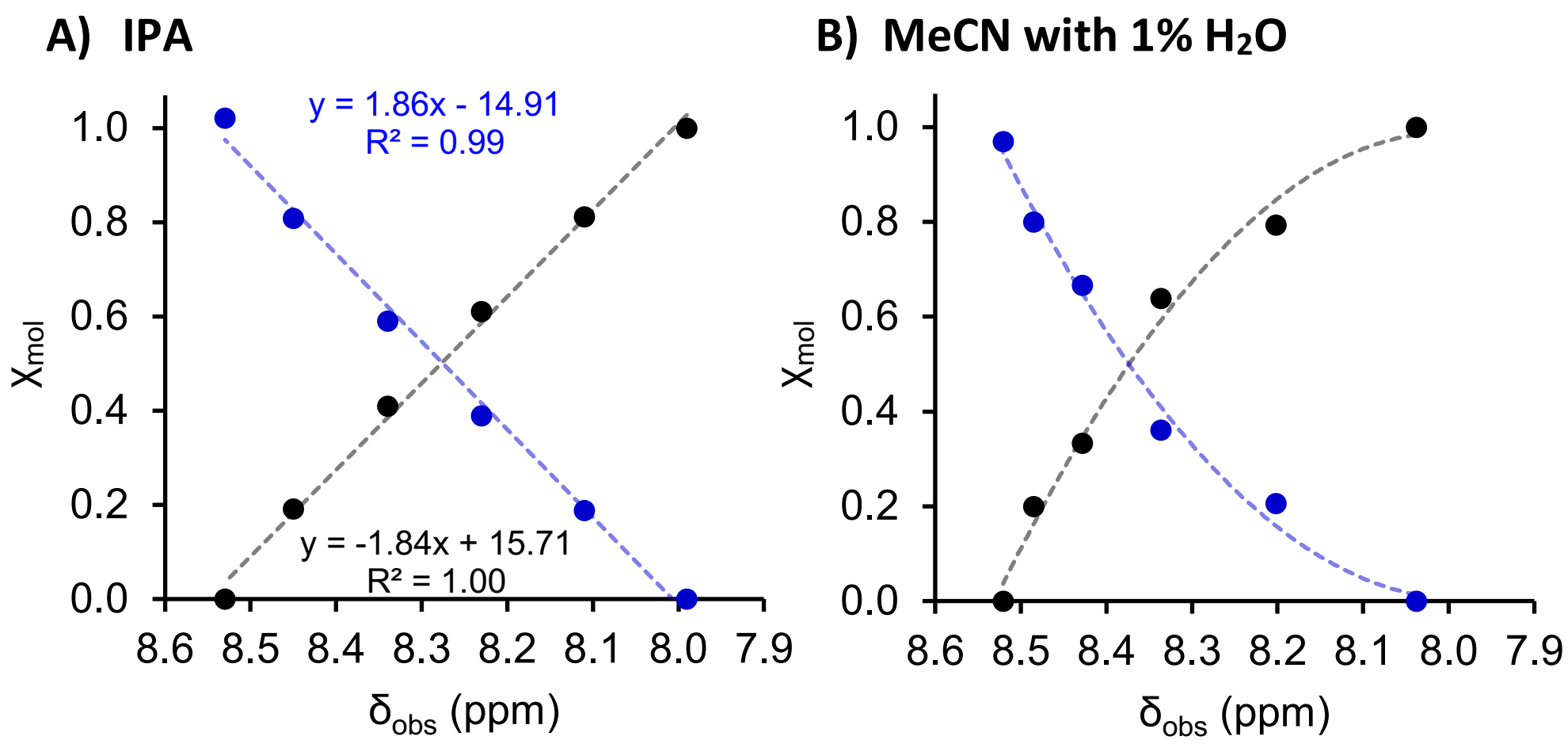

Figure S3. ^{1}H NMR chemical shift of the formyl proton during titration of a 11.6 mM HCO_2H solution with increasing amounts of a strong base (^{t}BuTMG) in IPA (A) and MeCN containing 1% H_2O (B). In IPA, the observed chemical shift varies linearly with the HCO_2H/HCO_2^- molar fraction, whereas in MeCN the dependence deviates from linearity.

In IPA, the observed ^{1}H NMR chemical shift of the formyl proton varied linearly with the molar fraction of formic acid over the concentration range studied. Accordingly, the relationship between the observed chemical shift (δ_{obs}) and the molar fraction of formic acid was linear. Under conditions of fast exchange on the NMR timescale, the observed chemical shift corresponds to the population-weighted average of the limiting chemical shifts of formic acid and formate. The limiting chemical shifts were determined by measuring authentic samples of HCO_2H and $[nBu_4N][HCO_2]$ in IPA, giving $\delta_{\mathrm{HCO_2H}} = 7.99$ ppm and $\delta_{\mathrm{HCO_2^-}} = 8.52$ ppm.

The limiting chemical shifts obtained from measuring authentic samples of formic acid and tetrabutylammonium formate in IPA are $\delta_{\mathrm{HCO_2H}} = 7.99$ ppm and $\delta_{\mathrm{HCO_2^-}} = 8.52$ ppm. Under these conditions, the observed chemical shift can be expressed in terms of the limiting chemical shifts and the molar fraction of formic acid:

$$\delta_{\mathrm{obs}} = x_{\mathrm{HCO_2H}}\delta_{\mathrm{HCO_2H}} + (1 - x_{\mathrm{HCO_2H}})\delta_{\mathrm{HCO_2^-}} \quad \text{(S3)}$$

$$x_{\mathrm{HCO_2H}} = \frac{\delta_{\mathrm{obs}} - \delta_{\mathrm{HCO_2^-}}}{\delta_{\mathrm{HCO_2H}} - \delta_{\mathrm{HCO_2^-}}} \quad \text{(S4)}$$

$$x_{\mathrm{HCO_2^-}} = 1 - x_{\mathrm{HCO_2H}} \quad \text{(S5)}$$

In contrast, in MeCN the dependence of the chemical shift on composition deviated from linearity. This behavior can be better described using an effective activity-weighted model to account for differences in the effective activities of HCO_2H and HCO_2^-. In MeCN with 1% H_2O, the limiting chemical shifts in MeCN are $\delta_{\mathrm{HCO2H}} = 8.03$ ppm and $\delta_{\mathrm{HCOO^-}} = 8.52$ ppm. By expressing the previous equations in terms of activities and activity coefficient, the following equations are obtained:

$$\delta_{obs} = \frac{a_{\mathrm{HCO_2H}}\delta_{\mathrm{HCO_2H}} + a_{\mathrm{HCO_2^-}}\delta_{\mathrm{HCO_2^-}}}{a_{\mathrm{HCO_2H}} + a_{\mathrm{HCO_2^-}}} \quad \text{(S6)}$$

$$\delta_{obs} = \frac{\gamma_{\mathrm{HCO_2H}}\chi_{\mathrm{HCO_2H}}\delta_{\mathrm{HCO_2H}} + \gamma_{\mathrm{HCO_2^-}}(1-\chi_{\mathrm{HCO_2H}})\delta_{\mathrm{HCO_2^-}}}{\gamma_{\mathrm{HCO_2H}}\chi_{\mathrm{HCO_2H}} + \gamma_{\mathrm{HCO_2^-}}(1-\chi_{\mathrm{HCO_2H}})} \quad \text{(S7)}$$

$$x_{\mathrm{HCO2H}} = \frac{R(\delta_{\mathrm{obs}} - \delta_{\mathrm{HCO_2^-}})}{\left(\delta_{\mathrm{HCO_2H}} - \delta_{\mathrm{obs}}\right) + R\left(\delta_{\mathrm{obs}} - \delta_{\mathrm{HCO_2^-}}\right)} \quad \text{(S8)}$$

where R represents the ratio of the effective activity coefficients of HCO_2H and HCO_2^- with nonlinear least-squares fitting of the titration data giving:

$$R = \frac{\gamma_{\mathrm{HCO_2^-}}}{\gamma_{\mathrm{HCO_2H}}} = 2.45 \pm 0.19 \quad \text{(S9)}$$

$$x_{\mathrm{HCO_2H}} = \frac{2.45(\delta_{\mathrm{obs}} - 8.52)}{(8.03 - \delta_{\mathrm{obs}}) + 2.45(\delta_{\mathrm{obs}} - 8.52)} \quad \text{(S10)}$$

Analysis of gaseous products was performed via GC on an Agilent 8890 system with a molesieve–carbon plot dual column layout with a TCD and a methanizer-FID detector set up and using Argon as a carrier gas. CO and H_2 were analyzed by integrating the corresponding TCD signal and using an external calibration curve.

4. Product crossover evaluation

Product crossover from the catholyte to the anolyte was evaluated in *cell ii* (*vide supra*). A known amount of formic acid (11.2 mM) was added to the catholyte, 100 mM [nBu_4N][PF_6] solution in MeCN 1% H_2O. Crossover from the catholyte to the anolyte was evaluated with and without applied current. In the latter, −1 mA current was applied for two hours under inert atmosphere using a Pd foil working electrode (1.40 cm^2), Ti foil counter electrode, and an Ag wire pseudo-reference electrode.

The concentration of each species was quantified using ^1H NMR spectroscopy. A 300 μL aliquot of both the catholyte and anolyte was taken before being added to the H-cell (t = 0 h) and after the two-hour reaction period (t = 2 h). A 300 μL aliquot of 22.8 mM 1,3,5-trimethoxybenzene in CD_3CN and 20.9 mM sodium trimethylsilylpropanesulfonate (DSS) in D_2O were added to the catholyte and anolyte, respectively. ^1H NMR spectra (32 scans) were obtained using D1 ≥ 5 × T_1 (40.67 and 54.95 s in CD_3CN and D_2O, respectively). The DSS -CH_2 peak at δ0.63 ppm (2H) and the aromatic hydrogens in 1,3,5-trimethoxybenzene at δ6.07 ppm (3H), in D_2O and CD_3CN, respectively, were normalized to the appropriate number of hydrogens, and the resulting integral for the formic acid peak (δ8.02 and δ8.24 ppm in CD_3CN and D_2O, respectively) was used to calculate the concentration of formic acid in the sample.

Based on the NMR data, two parameters describing the crossover are quantified: the percent of compound that passed across the membrane in the reaction time (% Crossover) and the flux ratio (χ_f) relating the moles of compound that crossed over to the moles of protons that passed through the membrane during electrolysis.

The % Crossover is calculated by taking the ratio of the initial moles of compound in one compartment and the final moles of compound found in the other compartment (n_{chem}). This calculation can be performed for reactions with or without current passed. Because comparable amounts of formic acid are detected in the anolyte with and without applied current, simple diffusion is the predominant crossover process.

The flux ratio (χ_f) can be calculated only for experiments that pass current. The moles of protons transferred during electrolysis, n_{H+}, can be calculated using equation S11:

$$n_{H^+} = \frac{it}{\mathrm{F}} \qquad \text{(S11)}$$

where i is the current (Amperes), t is the time (seconds), and F is Faraday's constant. Substituting the utilized experimental conditions of –1 mA of current for 2 hours into eq S11 gives 7.43 × 10^{-5} moles of protons passed across the membrane in each experiments (eq S12).

$$n_{H^+} = \frac{(1\times10^{-3}\ \mathrm{A})(7200\ \mathrm{s})}{9.6485\times10^{4}\ \mathrm{C\ mol^{-1}}} = 7.43\times10^{-5}\ \text{moles protons} \qquad \text{(S12)}$$

Equation S13 defines the flux ratio (χ_f), where n_{chem} and n_{H+} are the moles of formic acid and moles of protons, respectively, that migrated through the membrane during the experiment.

$$\chi_f = \frac{n_{chem}}{n_{H^+}} \tag{S13}$$

Table S1. Flux ratios and percent crossover for neutral molecules −1 mA current in two hours.

	χ_f	% Crossover
**Formic Acid**[a]	0.0783	5 ± 1%
**Acetonitrile**[a]	213	0.12 ± 0.008%
**Water**[b]	4.79	0.019 ± 0.005%

[a]Crossover evaluated from cathode to anode half cell
[b]Crossover evaluated from anode to cathode half cell

Table S2. Initial and final formic acid concentrations in the catholyte and anolyte with -1 mA applied current.

	**[Formic Acid] in Catholyte (mM)**		**[Formic Acid] in Anolyte (mM)**		**% Crossover**
	t = 0 hr	*t = 2 hr*	*t = 0 hr*	*t = 2 hr*	
**Trial 1**	11.5	10.4	0	0.445	3.9%
**Trial 2**	11.4	11.6	0	0.771	6.8%
**Trial 3**	11.2	11.5	0	0.531	4.7%
Average	*11.4 ± 0.6*	*11.2 ± 0.6*	*0*	*0.58 ± 0.2*	*5.1% ± 1.5%*

Table S3. Initial and final formic acid concentrations in the catholyte and anolyte without applied current.

	**[Formic Acid] in Catholyte (mM)**		**[Formic Acid] in Anolyte (mM)**		**% Crossover**
	t = 0 hr	*t = 2 hr*	*t = 0 hr*	*t = 2 hr*	
**Trial 4**	12.6	11.5	0	0.453	3.6%
**Trial 5**	10.6	9.99	0	0.428	4.0%
**Trial 6**	11.6	12.2	0	0.497	4.3%
Average	*11.6 ± 0.1*	*11.2 ± 1*	*0*	*0.46 ± 0.03*	*4.0% ± 0.3%*

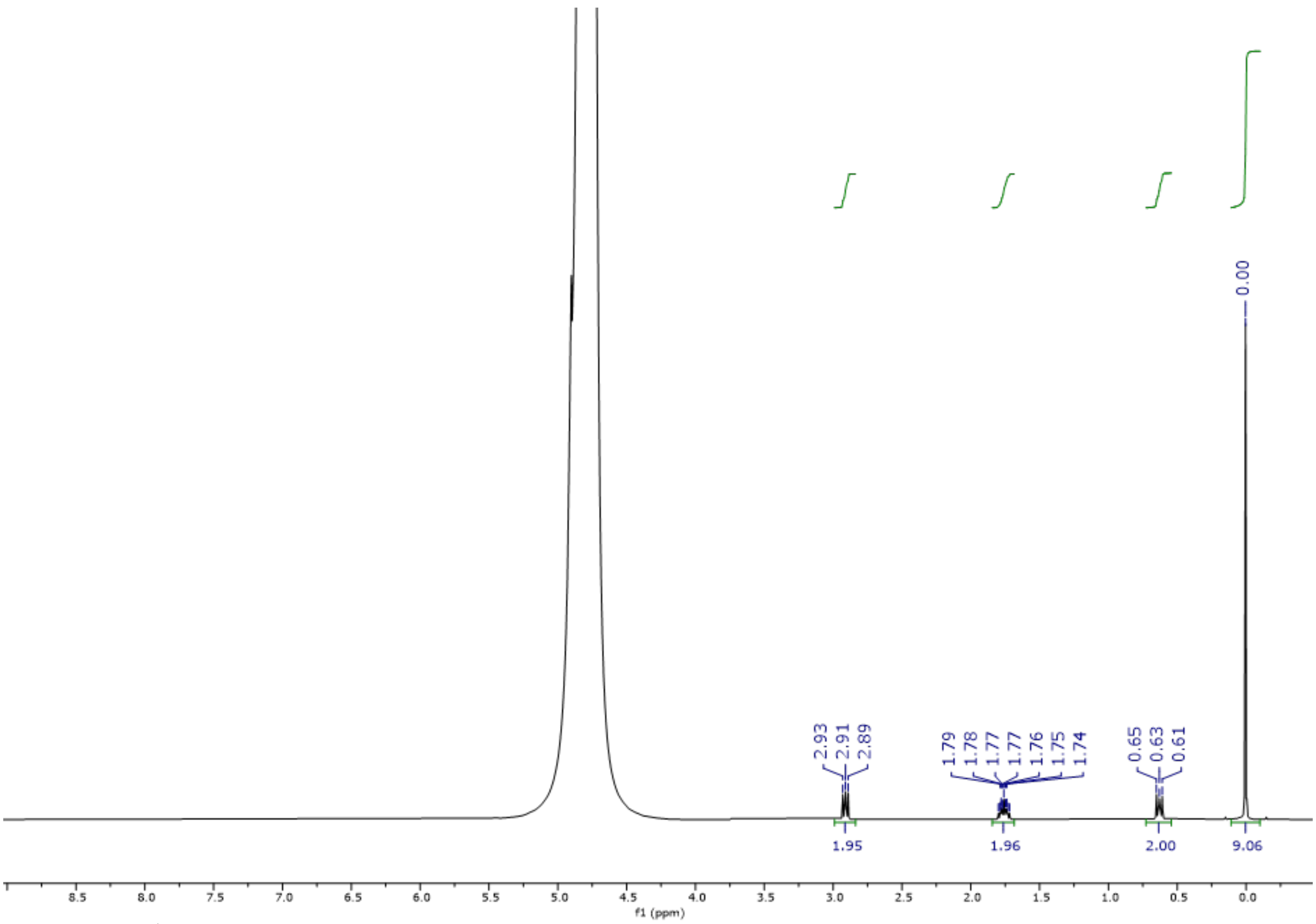

Figure S4. ^{1}H NMR spectrum of anolyte from before crossover study with applied current in D_2O at t = 0 h. DSS is used as an internal standard.

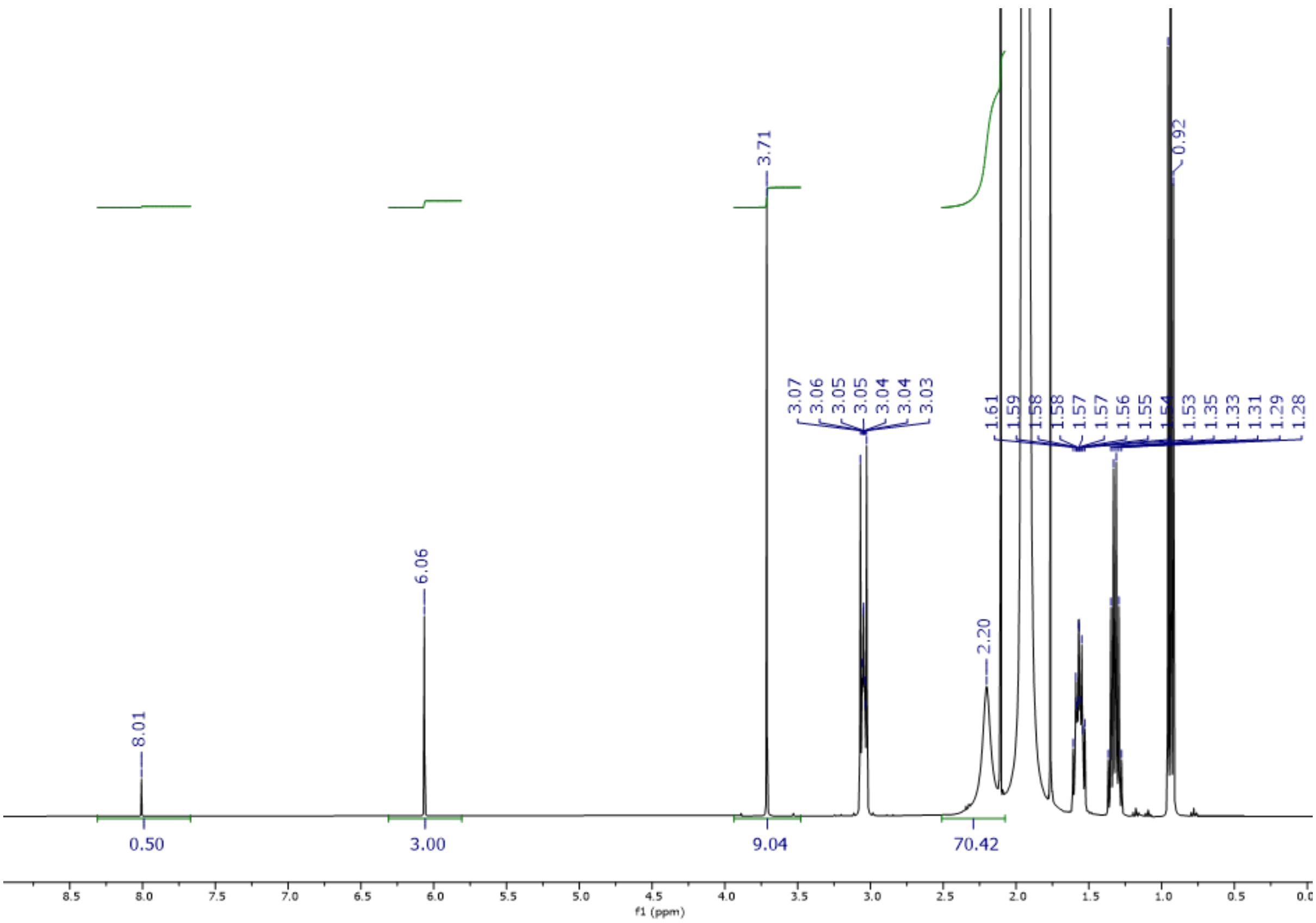

Figure S5. ^{1}H NMR spectrum of catholyte from before crossover study with applied current in CD_3CN at t = 0 h. Trimethoxybenzene is used as an internal standard. Formic acid is observed at δ8.01 ppm.

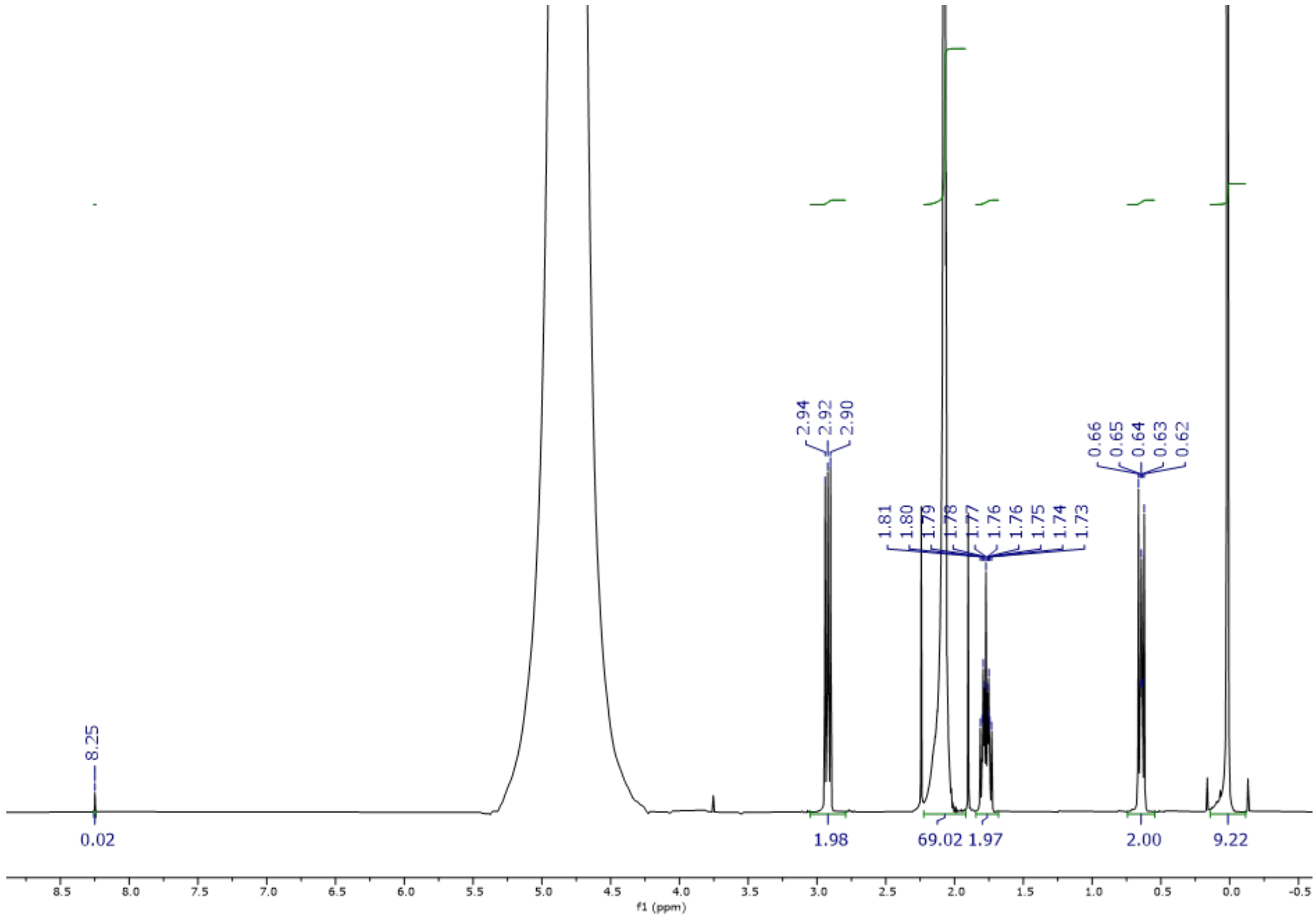

Figure S6. ^{1}H NMR spectrum of anolyte from crossover study with applied current in D_2O at t = 2 h (i = -1 mA, j = 0.71 mA cm^{-2}). Formic acid is observed at δ8.25 ppm. Acetonitrile is observed at ca. δ 2.1 ppm.

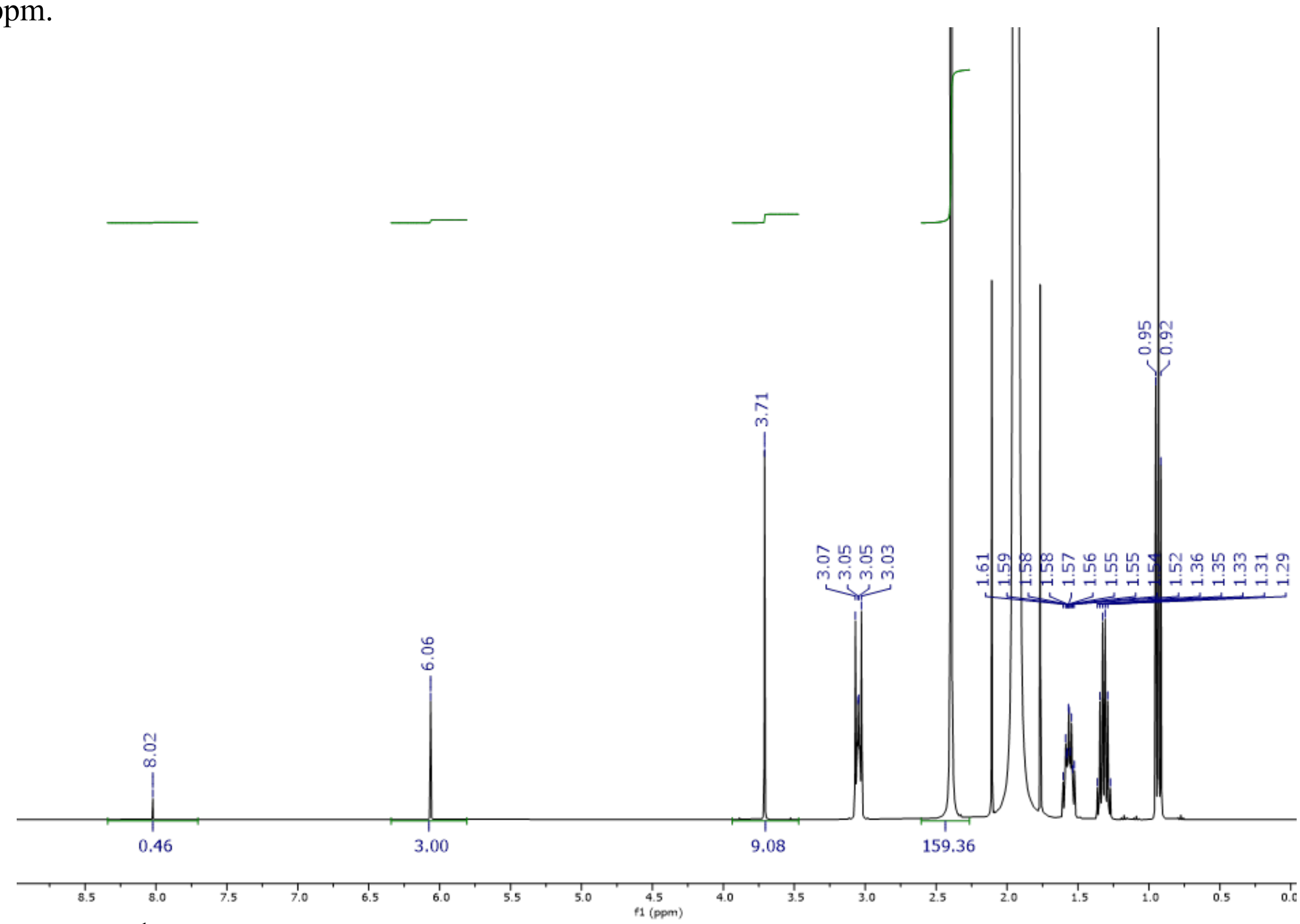

Figure S7. ^{1}H NMR spectrum of catholyte from crossover study with applied current in CD_3CN at t = 2 h (i = -1 mA, j = 0.71 mA cm^{-2}). Formic acid is observed at δ8.02 ppm. Water is observed at ca. 2.4 ppm. Trimethoxybenzene is used as an internal standard.

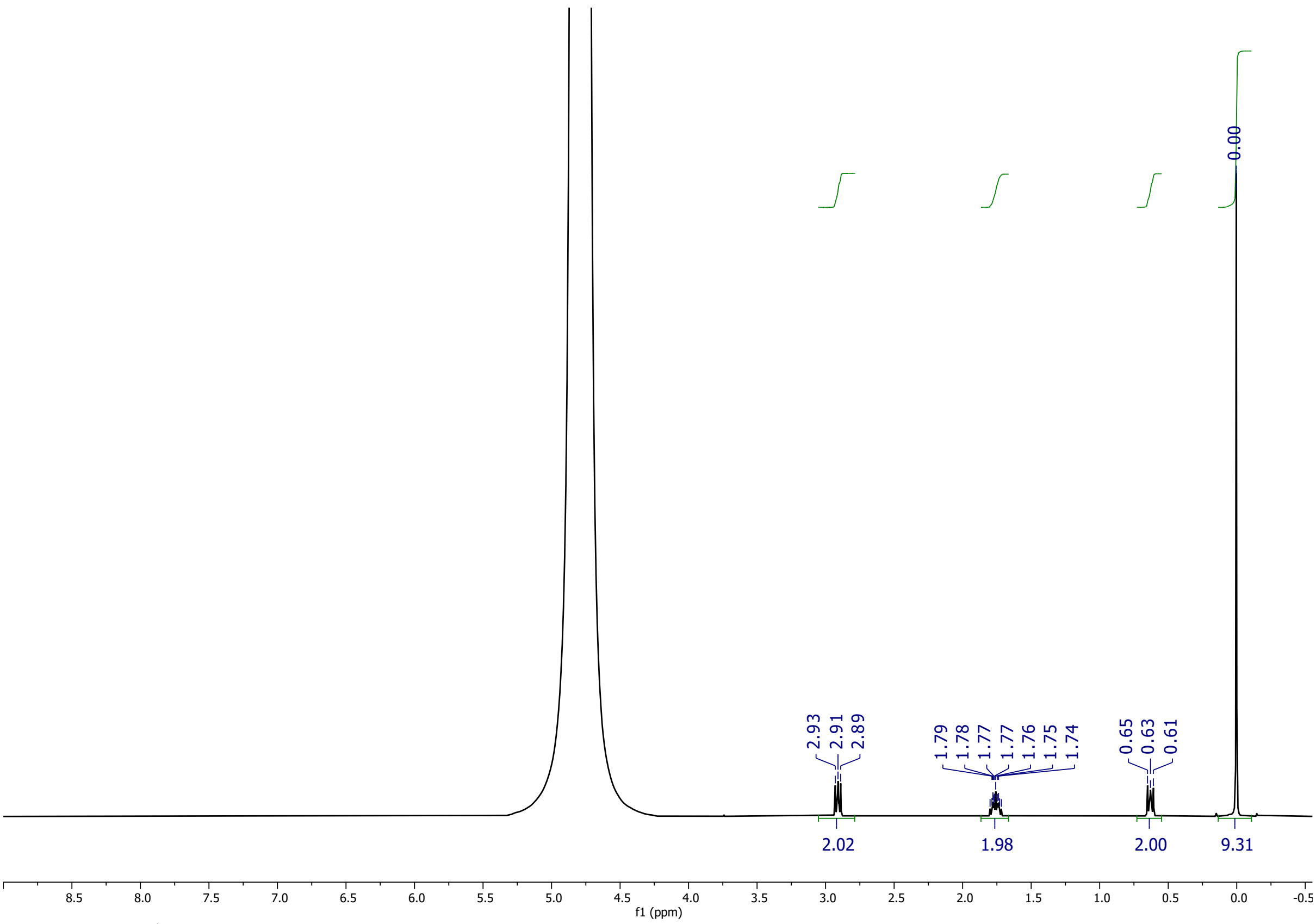

Figure S8. ^{1}H NMR spectrum of anolyte from before cross-over study without applied current in D_2O at t = 0 h. DSS is used as an internal standard.

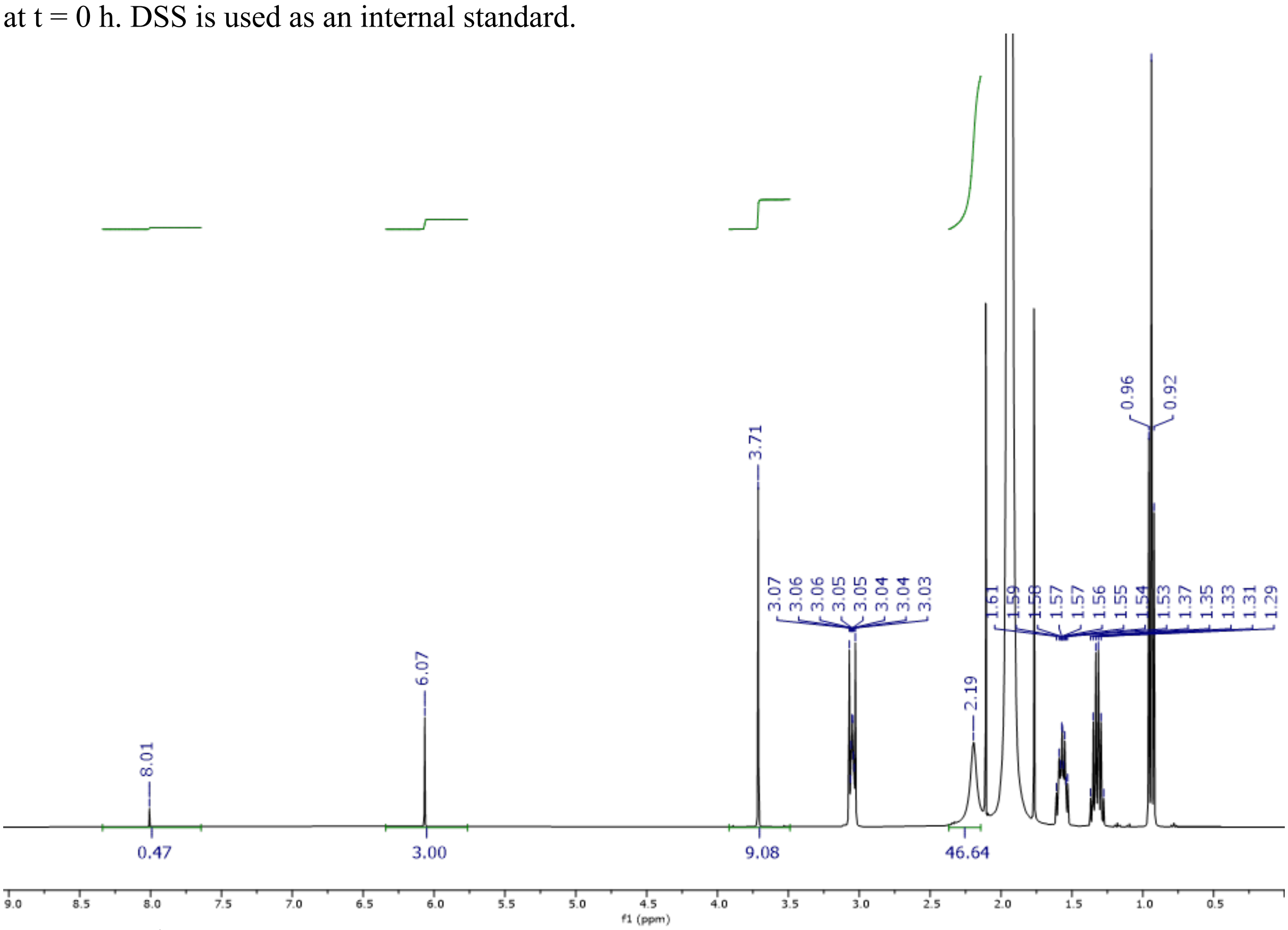

Figure S9. ^{1}H NMR spectrum of catholyte from before cross-over study without applied current in CD_3CN at t = 0 h. Trimethoxybenzene is used as an internal standard. Formic acid is observed at δ8.01 ppm.

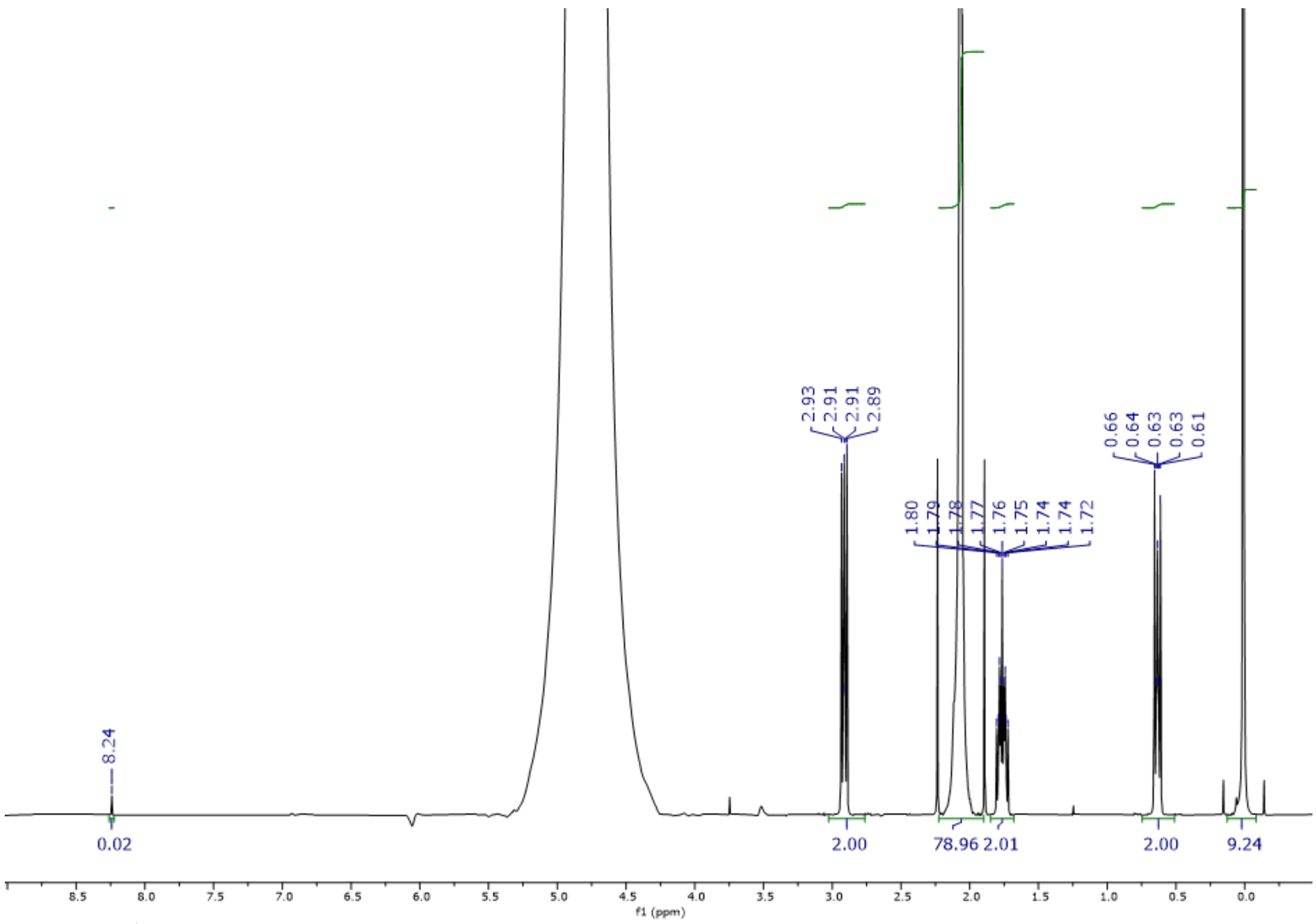

Figure S10. ^{1}H NMR spectrum of anolyte from cross-over study without applied current in D_2O at t = 2 h. Formic acid is observed at δ8.24 ppm. Acetonitrile is observed at ca. δ 2.1 ppm. DSS is used as an internal standard.

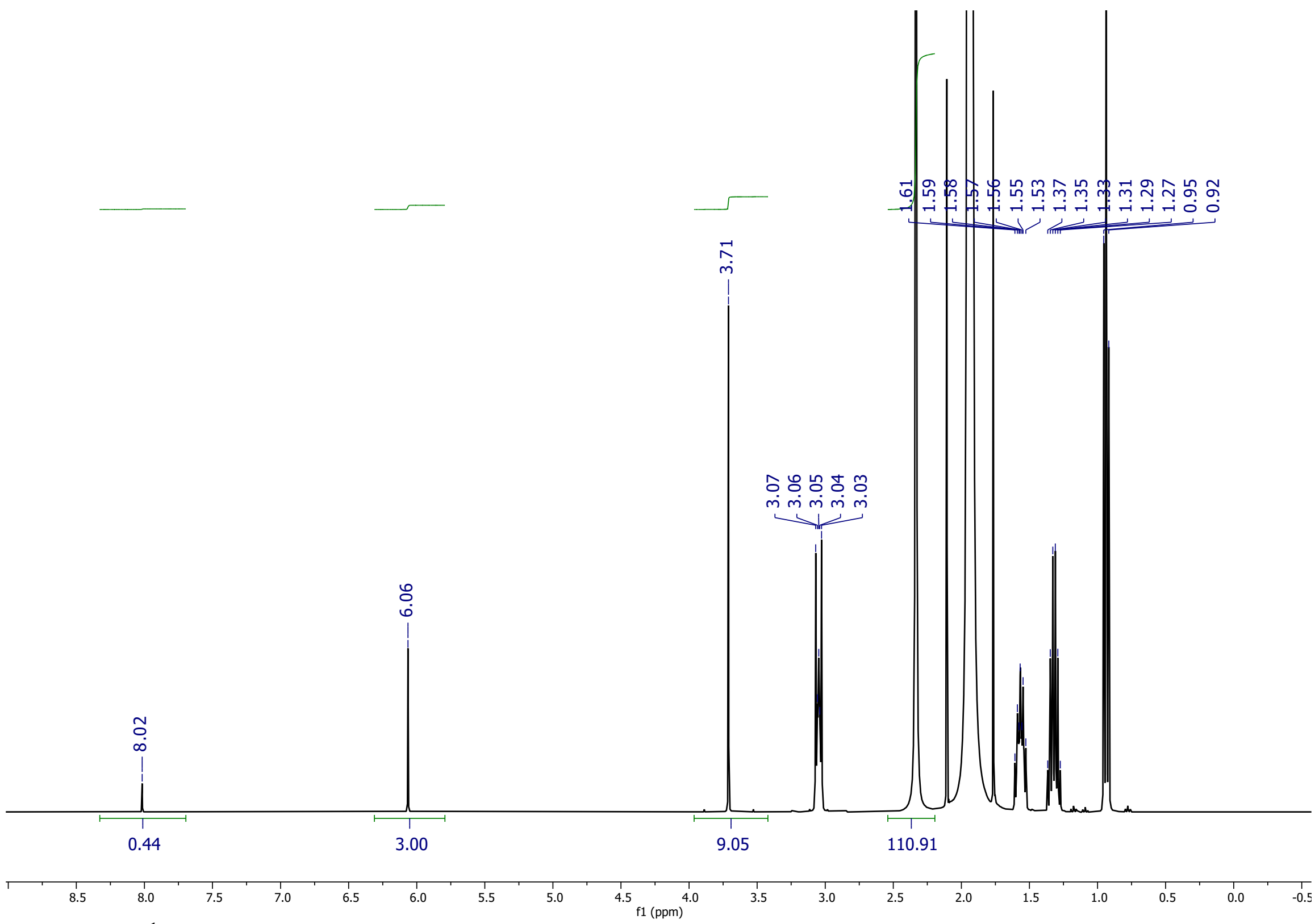

Figure S11. ^{1}H NMR spectrum of catholyte from cross-over study without applied current in CD_3CN at t = 2 h. Formic acid is observed at δ8.02 ppm. Water is observed at ca. 2.4 ppm. Trimethoxybenzene is used as an internal standard.

Proton transport evaluation

A control experiment has been designed to evaluate the extent of passive acid crossover in the dual-electrolyte PEM cell. Under stirring, a 20 mM [nBu$_4$N][HCO$_2$] solution in MeCN 1% H_2O was placed in the cathodic compartment and separated from a 5 mM H_2SO_4 aqueous anolyte by Nafion 117. The HCO_2^- signal was monitored by ^{1}H NMR spectroscopy over 2 h (Figure 12). At time 0, the formate signal appeared at 8.53 ppm and gradually shifted to 8.45 ppm after 2 h. Based on the established relationship between chemical shift and HCO_2^-/HCO_2H speciation, this change corresponds to a decrease in the HCO_2^- fraction from ca. 100% HCO_2^- to 70% HCO_2^-, indicating partial protonation of HCO_2^- upon contact of the catholyte with the PEM.

After 2 hours, the cathode solution was replaced by a fresh HCO_2^- solution and followed by ^{1}H NMR for an additional hour. In this case, the formate resonance remained unchanged at 8.53 ppm, indicating that no additional protonation occurred. These results suggest that the initial acidification primarily arises from exchange of membrane-bound H$^+$ with [nBu$_4$N]$^+$, rather than from sustained passive proton crossover from the anolyte.

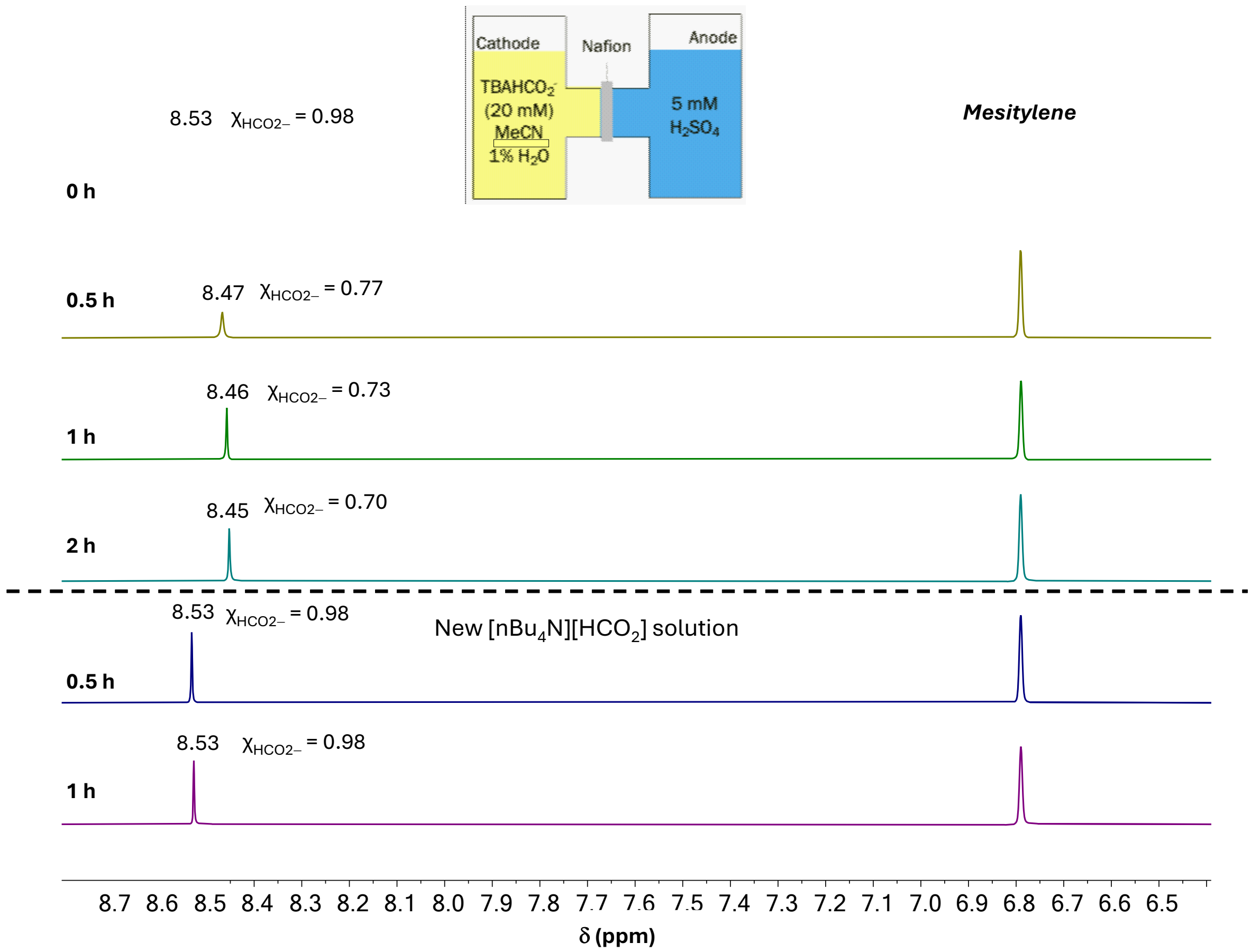

Figure S12. ^{1}H NMR monitoring of 20 mM [nBu$_4$N][HCO$_2$] solution in MeCN 1% H_2O placed in the cathodic compartment and separated from a 5 mM H_2SO_4 aqueous anolyte by Nafion 117 under stirring in the dual-electrolyte H-cell. Spectra above the dashed line correspond to the initial solution of [nBu$_4$N][HCO$_2$] monitored over 2 h. The catholyte was then replaced with a fresh [nBu$_4$N][HCO$_2$] solution, which was monitored for an additional hour, as shown in the spectra below the dashed line. Mesitylene (20 mM) and MeCN-*d$_3$* (10%) were used as an internal standard and deuterated solvent, respectively.

Experiments in the presence of an organic dye

Bromocresol green was selected as a qualitative indicator of the acidity distribution within the catholyte because, its color transition from yellow (acidic form) to green-blue (basic form) occurs within the pH range over which HCO_2H and HCO_2^- coexist in equilibrium. Experiments were performed in the same dual-electrolyte H-cell used for CPE, with the catholyte containing 50 µM bromocresol green in MeCN 1% H_2O with 0.1 M [nBu$_4$N][PF$_6$], separated from a 5 mM aqueous H_2SO_4 anolyte by a Nafion 117 membrane. At the beginning of the experiment, [nBu$_4$N][OH] was added to the catholyte to ensure the dye started in its basic form (green-blue). Then, the temporal and spatial color evolution of the solution was monitored under non-stirred electrochemical CO_2 reduction conditions (Figure 13).

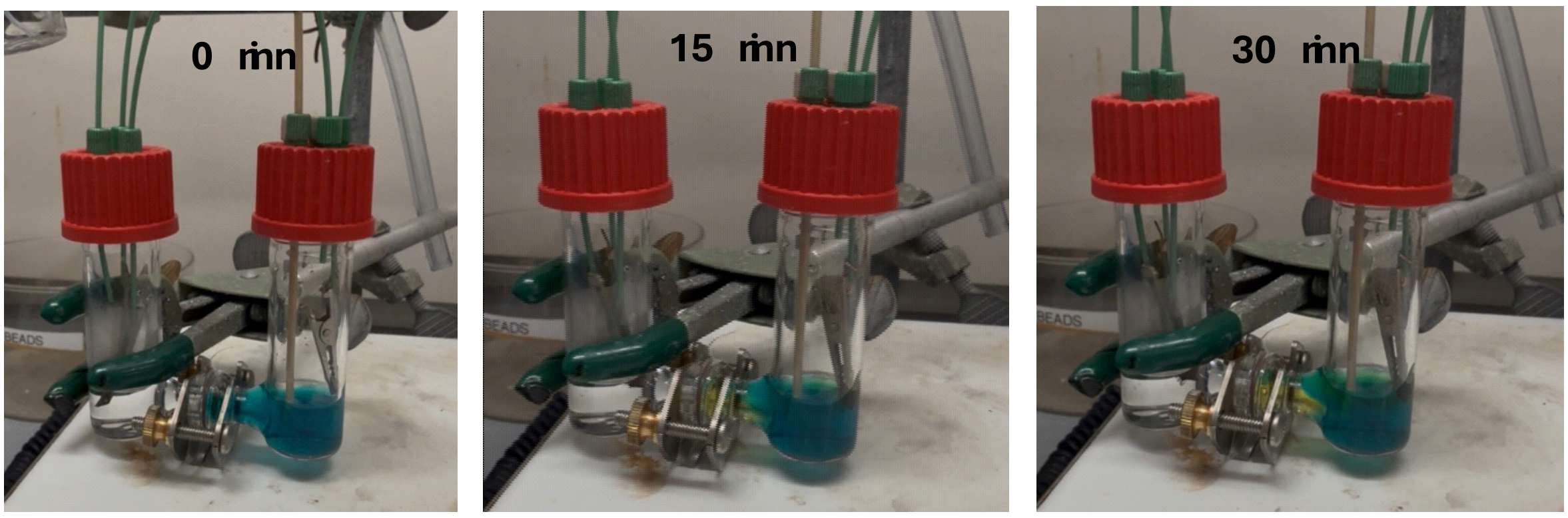

Figure S13. Colorimetric evolution of bromocresol green without stirring under electrocatalytic CO_2 reduction conditions. CPE was run in 0.1 M [nBu$_4$N][PF$_6$] MeCN (1% H_2O) electrolyte, charged with 2 mM [Ir–Cl]Cl, and 50 µM bromocresol green in a dual-electrolyte H-cell. The cell was sparged with CO_2 for 20 minutes and then 150 mM [nBu$_4$N][OH] stock solution was added in 20 µL increments under counterflow of CO_2 until the catholyte turned fully blue for a total of 300 µL. A color gradient was observed, wherein the solution turns yellow near the membrane and remains blue in the bulk and near the cathode.

5. Controlled potential electrolysis

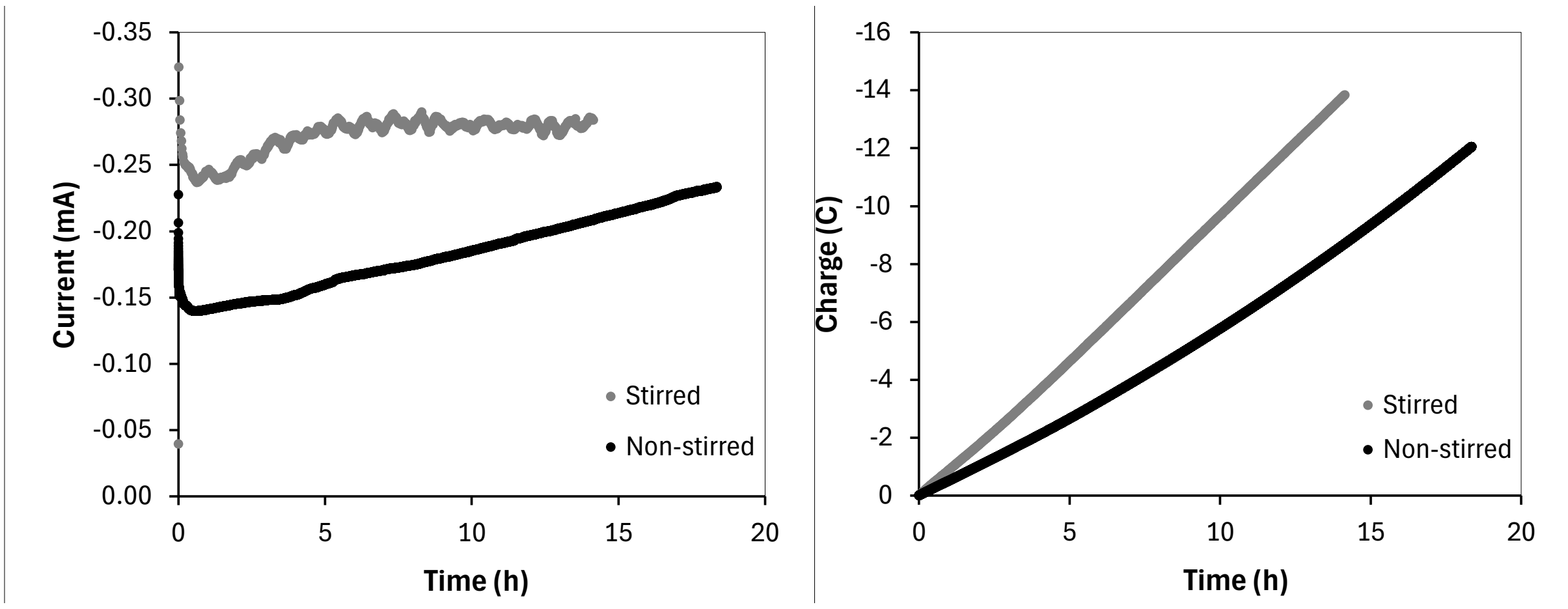

Figure S14. Current (left) and charge (right) over time from controlled potential electrolysis experiments with **Ir**$_{\mathbf{Cat}}$ (2 mM) in IPA, 0.1 M [nBu$_4$N][OTf] at -2.1 V vs. Fc$^{+/0}$ under CO_2 atmosphere in a PEM cell (cell ii) with stirring (gray) and in non-stirred conditions (black).

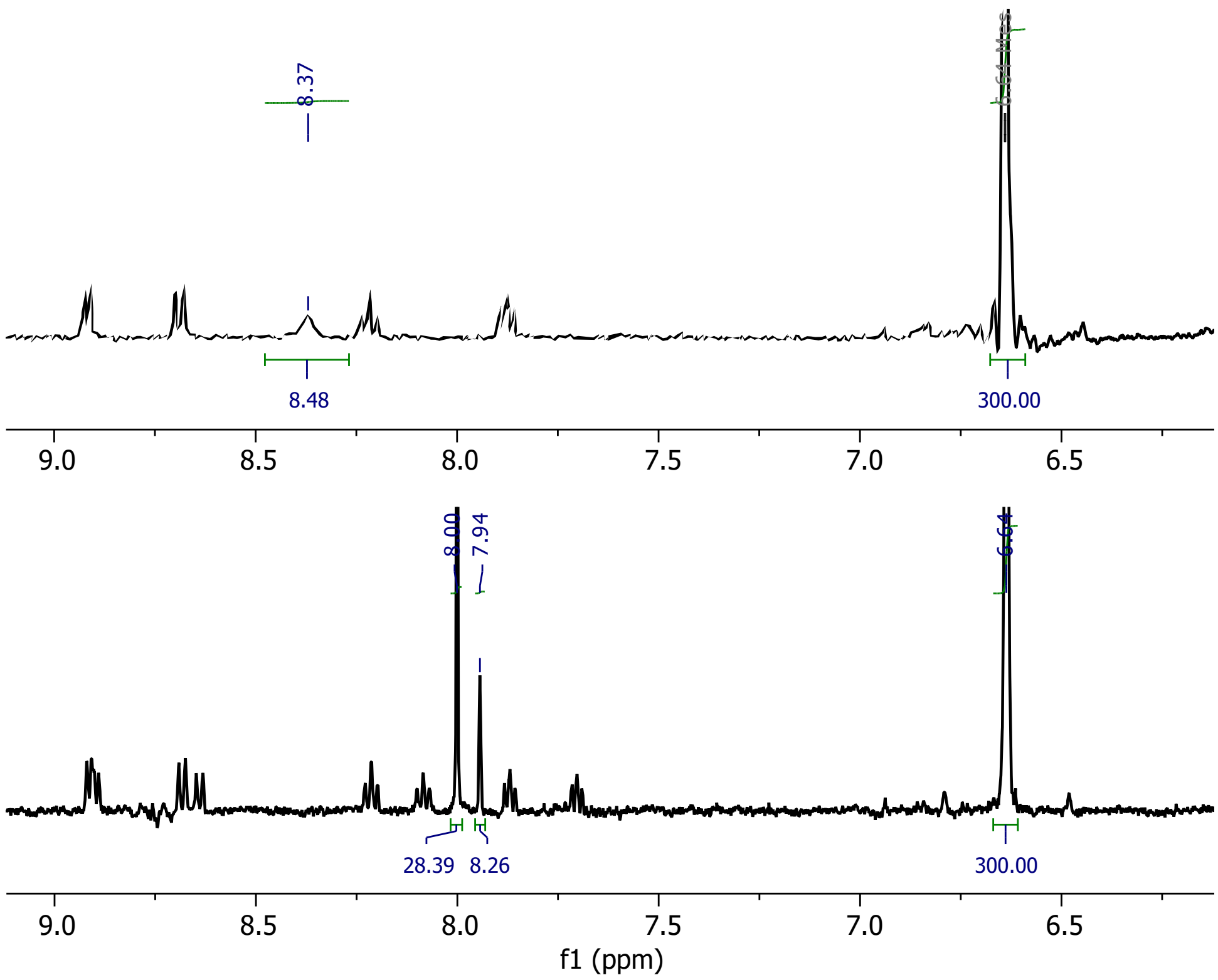

Figure S15. ^{1}H NMR spectra after CPE in a PEM cell (cell ii) under stirring (top) non-stirring (bottom). Mesitylene is used as internal standard (δ_{Mes} = 6.64 ppm, 20 mM). See Figure S14 for the corresponding CPE time traces. Under stirring, the formyl peak appears as a broad signal at 8.37 ppm which corresponds to χ_{HCO2H} = 0.36. Under non-stirring there are two formyl peaks, the one at 8.00 ppm corresponds to formic acid χ_{HCO2H} = 1.0 and the one at 7.94 ppm corresponds to $HCO_2{}^iPr$. Conditions: **Ir**$_{\mathbf{Cat}}$ (2 mM) in IPA, 0.1 M [nBu$_4$N][OTf] at -2.1 V vs. Fc$^{+/0}$ under CO_2 atmosphere.

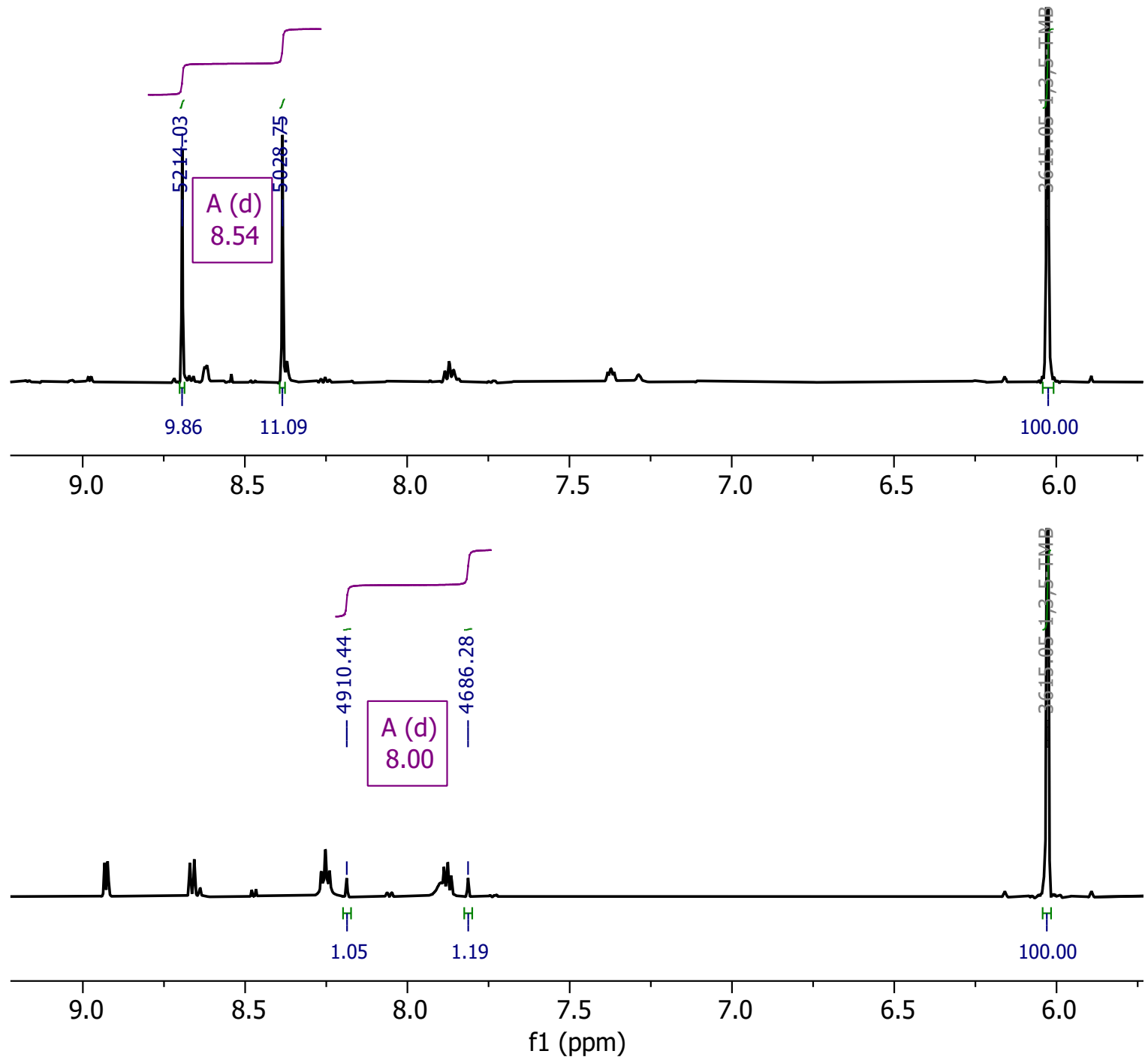

Figure S16. ^{1}H NMR spectra of post-electrolysis samples under $^{13}CO_2$. Bottom spectrum: sample after CPE in a PEM cell without stirring (cell ii). Top spectrum: the same sample after addition of ^{t}BuTMG. The singlet at 6.03 ppm corresponds to 1,3,5-trimethoxybenzene standard (10 mM). Conditions: **Ir$_{Cat}$** (2 mM) in IPA, 0.1 M [nBu$_4$N][OTf] at -2.1 V vs. $Fc^{+/0}$ under $^{13}CO_2$ atmosphere.

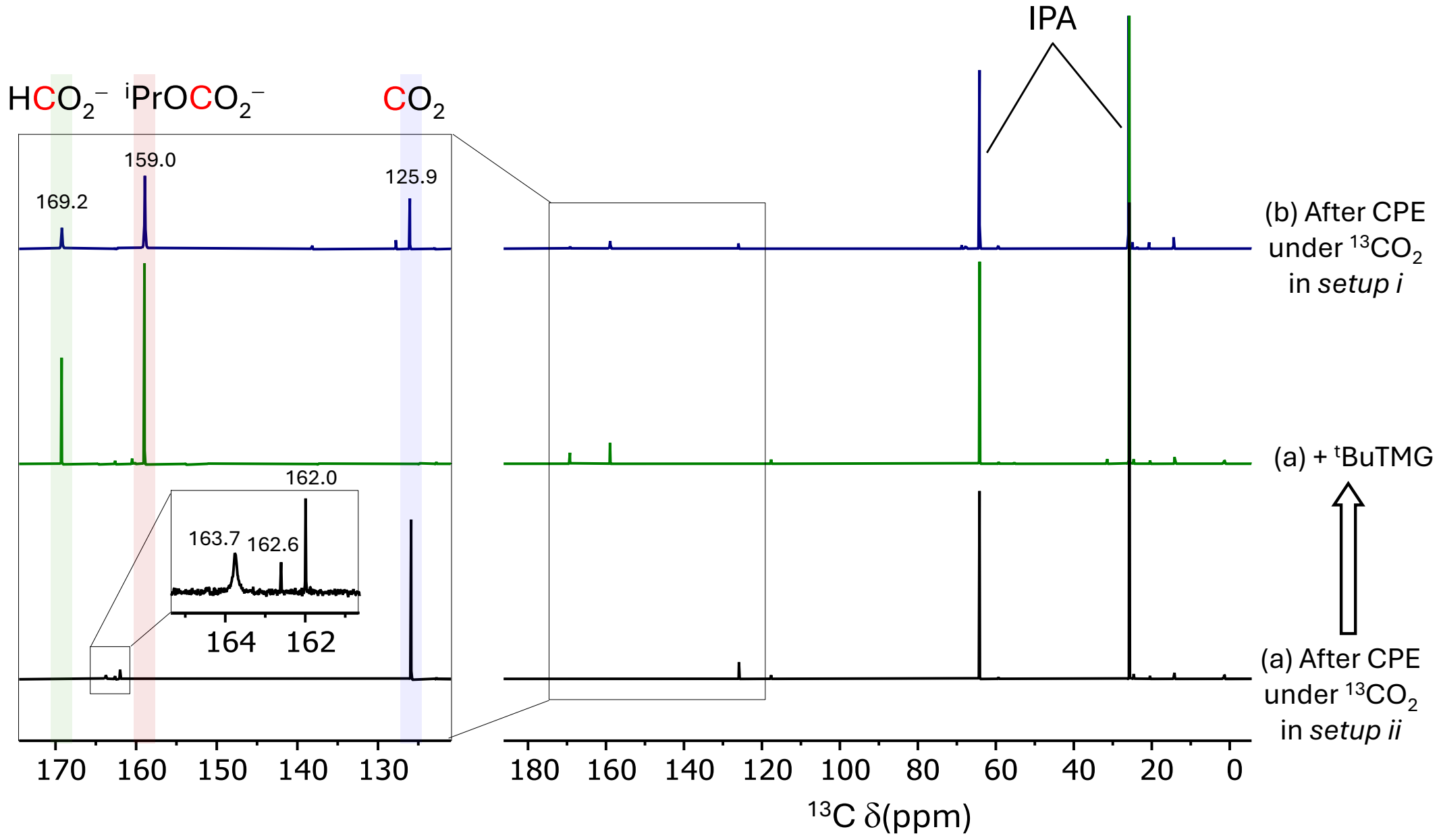

Figure S17. ^{13}C NMR spectra of post-electrolysis samples under $^{13}CO_2$. Bottom spectrum: sample after CPE in a PEM cell without stirring (cell ii). Middle spectrum: the same sample after basification by addition of ^{t}BuTMG. Top spectrum: sample after CPE in a frit-separated H-cell under $^{13}CO_2$ (cell i). A zoom into the carbonyl region is shown, with an inset highlighting the signals corresponding to HCO_2H (broad, $\delta = 163.7$), $HCO_2{}^{i}Pr$ ($\delta = 162.6$) and another signal at 162.0 ppm which is presumably related to carbonic acid.[6] The basic species HCO_2^- ($\delta = 169.2$) and $^{i}PrOCO_2^-$ ($\delta = 159.0$)[7] are not detected under acidity gradient control, but become observable after basification with ^{t}BuTMG or in a regular frit-separated H-cell (cell i). These results support that the control over acidity gradients suppress the formation of CO_2-derived basic species. Conditions: **Ir$_{Cat}$** (2 mM) in IPA, 0.1 M [nBu$_4$N][OTf] at -2.1 V vs. $Fc^{+/0}$ under $^{13}CO_2$ atmosphere.

Table S4. CPE results with **Ir**$_{\mathbf{Cat}}$ (2 mM) in IPA, 0.1 M [nBu$_4$N][OTf] at -2.1 V vs. Fc$^{+/0}$ under CO_2 atmosphere for ca. 16 hours.

Catalyst	Conditions	Q (C)	[HCO$_2$(H)] (mM)	FE (%)	δ_{obs} (ppm)	χ_{HCO2H}
**Ir**$_{\mathbf{Cat}}$	Glass frit	20	10.4	50	8.53	0.0
**Ir**$_{\mathbf{Cat}}$	PEM stirring	14	1.8	10	8.36	0.35
**Ir**$_{\mathbf{Cat}}$	PEM non-stirring[a]	1.9	1.2	48	8.00	1.0
**Ir**$_{\mathbf{Cat}}$	PEM non-stirring[b]	1.9	1.4	56	7.99	1.0
**Ir**$_{\mathbf{Cat}}$	PEM non-stirring	12	7.3	47	8.00	1.0
**Ir**$_{\mathbf{Cat}}$	PEM non-stirring	9	5.1	44	8.03	0.93

[a]Sampling near the cathode after 2 hours CPE. [b]Sampling near the membrane after 2 hours CPE.

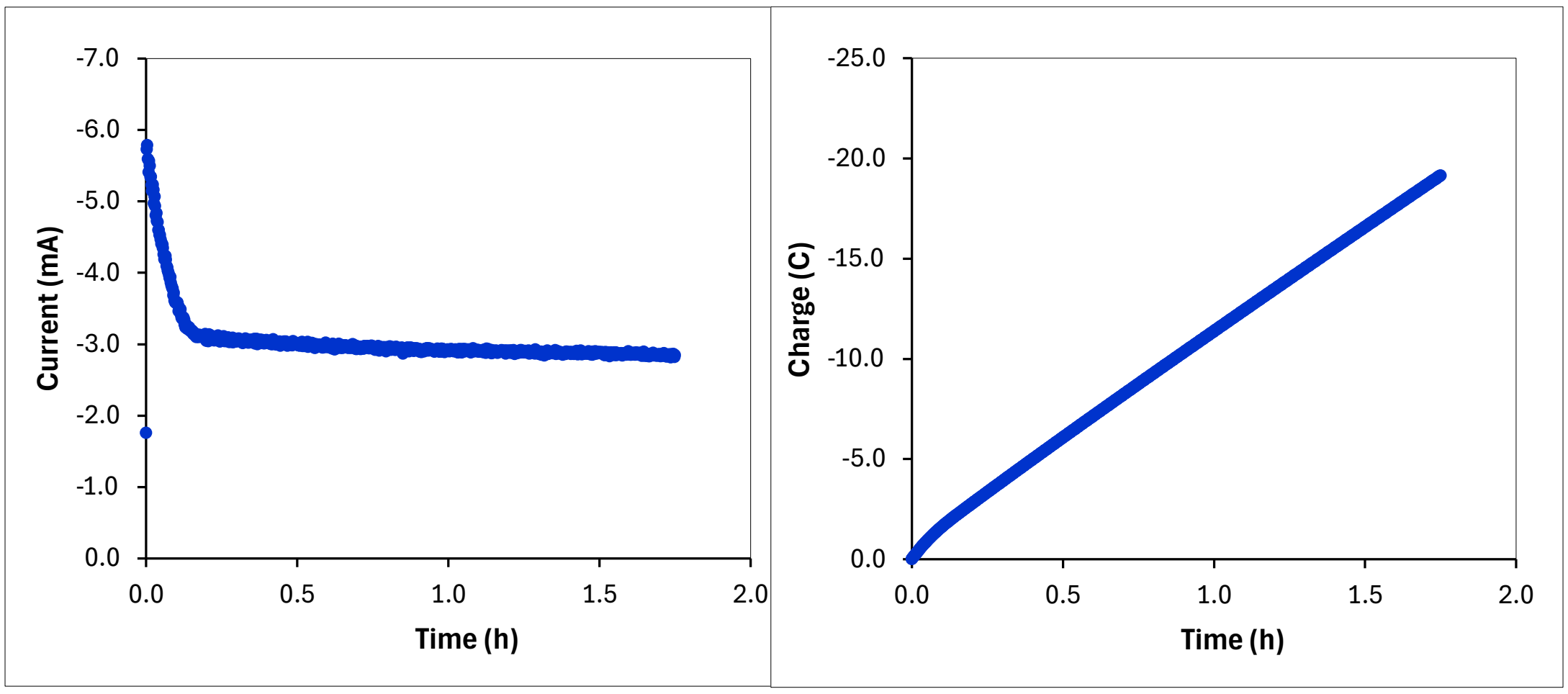

Figure S18. Current (left) and charge (right) over time from controlled potential electrolysis experiments with **Ir**$_{\mathbf{Cat}}$ (2 mM) in MeCN, 0.1 M [nBu$_4$N][PF$_6$] at -2.0 V vs. Fc$^{+/0}$ under CO_2 atmosphere in a frit-separated H-cell (cell i).

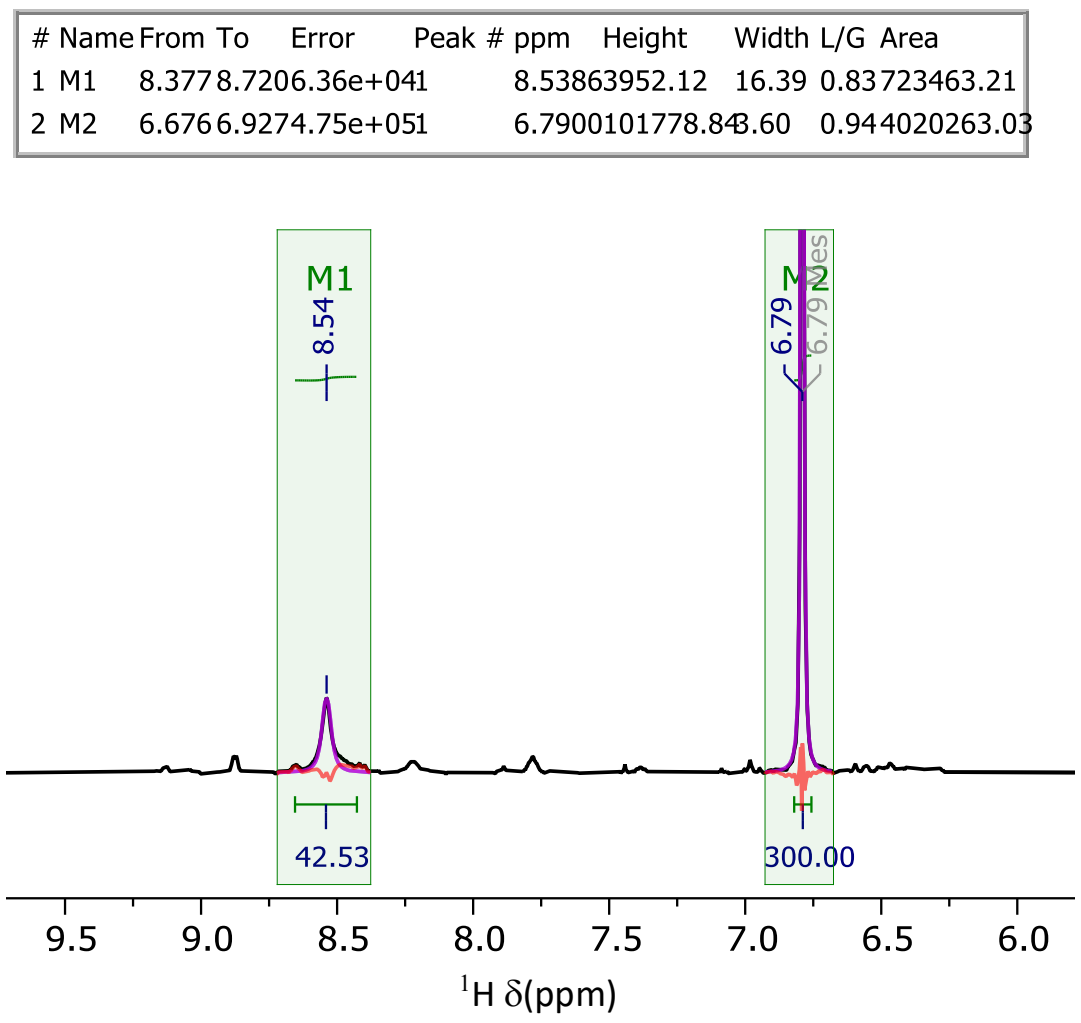

Figure S19. ^{1}H NMR spectra of post-electrolysis samples under CO_2 in a glass frit-separated H-cell (cell i). Both the mesitylene internal standard (6.79 ppm, 20 mM) and the formate signals (8.54 ppm) were fitted to correct for partial signal overlap. The mesitylene and formate signals were fitted to account for partial signal overlap. Integration relative to the internal standard gives a formate concentration of 10.8 mM, corresponding to 44% Faradaic efficiency. Conditions: **Ir**$_{\mathbf{Cat}}$ (2 mM) in MeCN, 0.1 M [nBu$_4$N][PF$_6$] at -2.0 V vs. Fc$^{+/0}$ under CO_2 atmosphere.

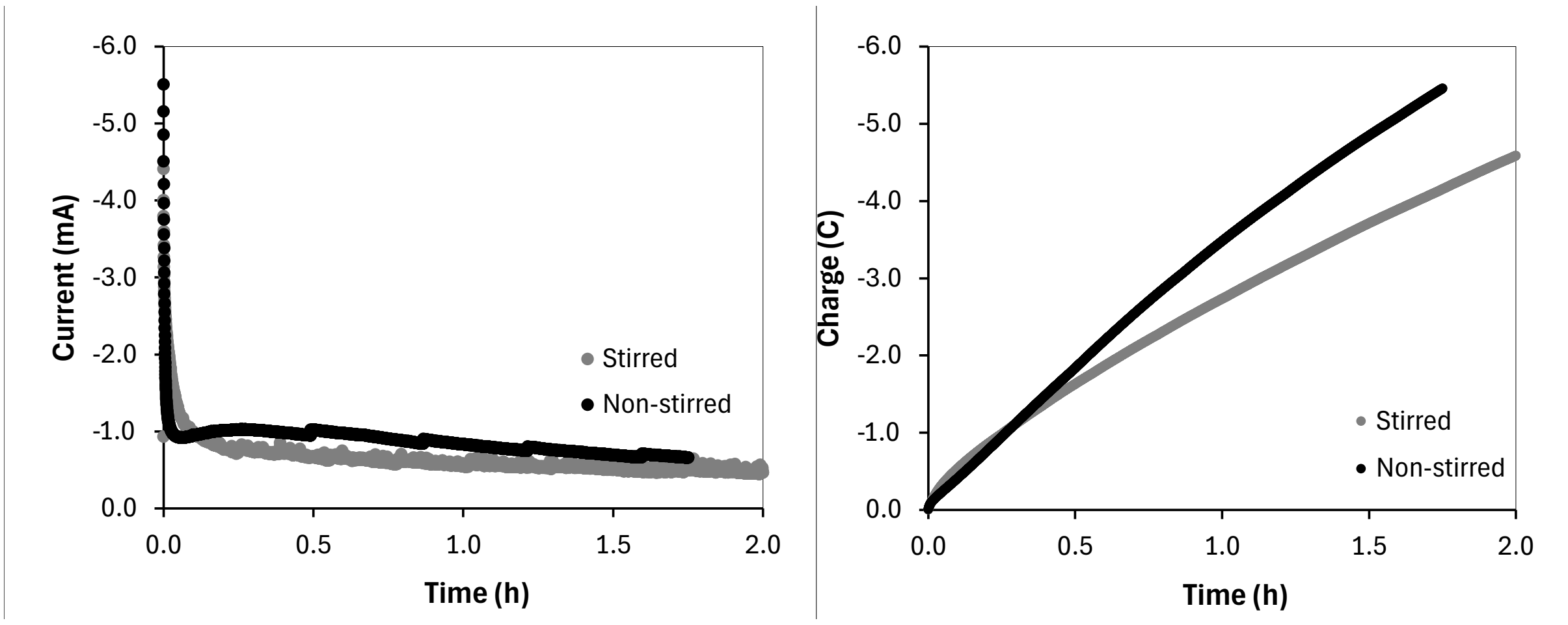

Figure S20. Current (top left) and charge (top right) over time from controlled potential electrolysis experiments with **Ir$_{Cat}$** (2 mM) in MeCN, 0.1 M [nBu$_4$N][PF$_6$] at -2.0 V vs. Fc$^{+/0}$ under CO_2 atmosphere in a PEM cell (cell ii) under stirring (gray) and non-stirring (black).

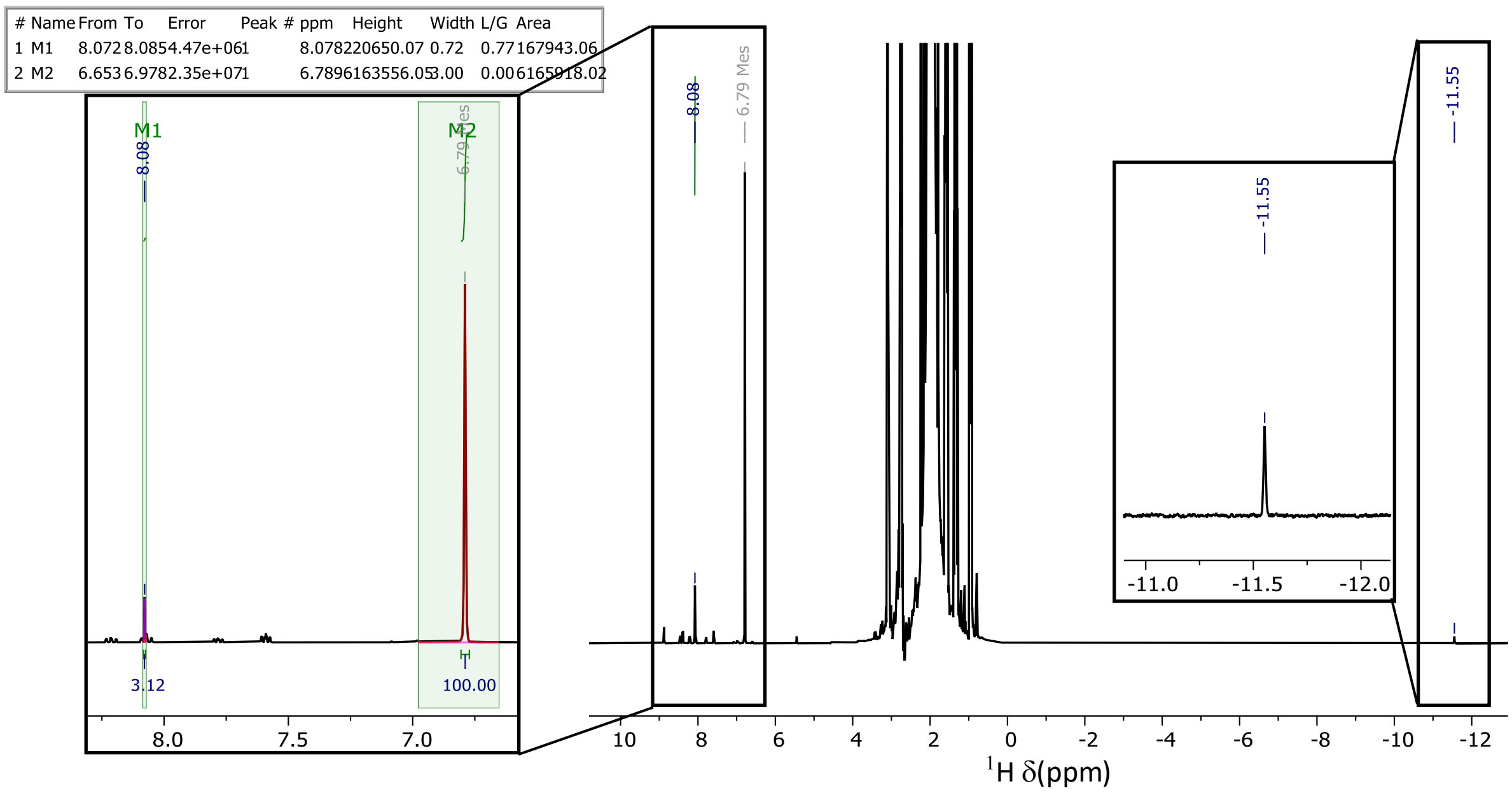

Figure S21. ^{1}H NMR spectra of post-electrolysis samples under CO_2 in a PEM cell without stirring (cell ii). The singlet at 6.79 ppm corresponds to the mesitylene internal standard (20 mM). Close-ups of the formyl and hydride spectral regions are shown. Under non-stirring, the formyl peak appears at 8.08 ppm which corresponds to χ_{HCO2H} = 0.96. Both the mesitylene internal standard and the formic acid signals were fitted to correct for partial signal overlap. The mesitylene and formate signals were fitted to account for partial signal overlap. Conditions: **Ir$_{Cat}$** (2 mM) in MeCN, 0.1 M [nBu$_4$N][PF$_6$] at -2.0 V vs. Fc$^{+/0}$ under CO_2 atmosphere.

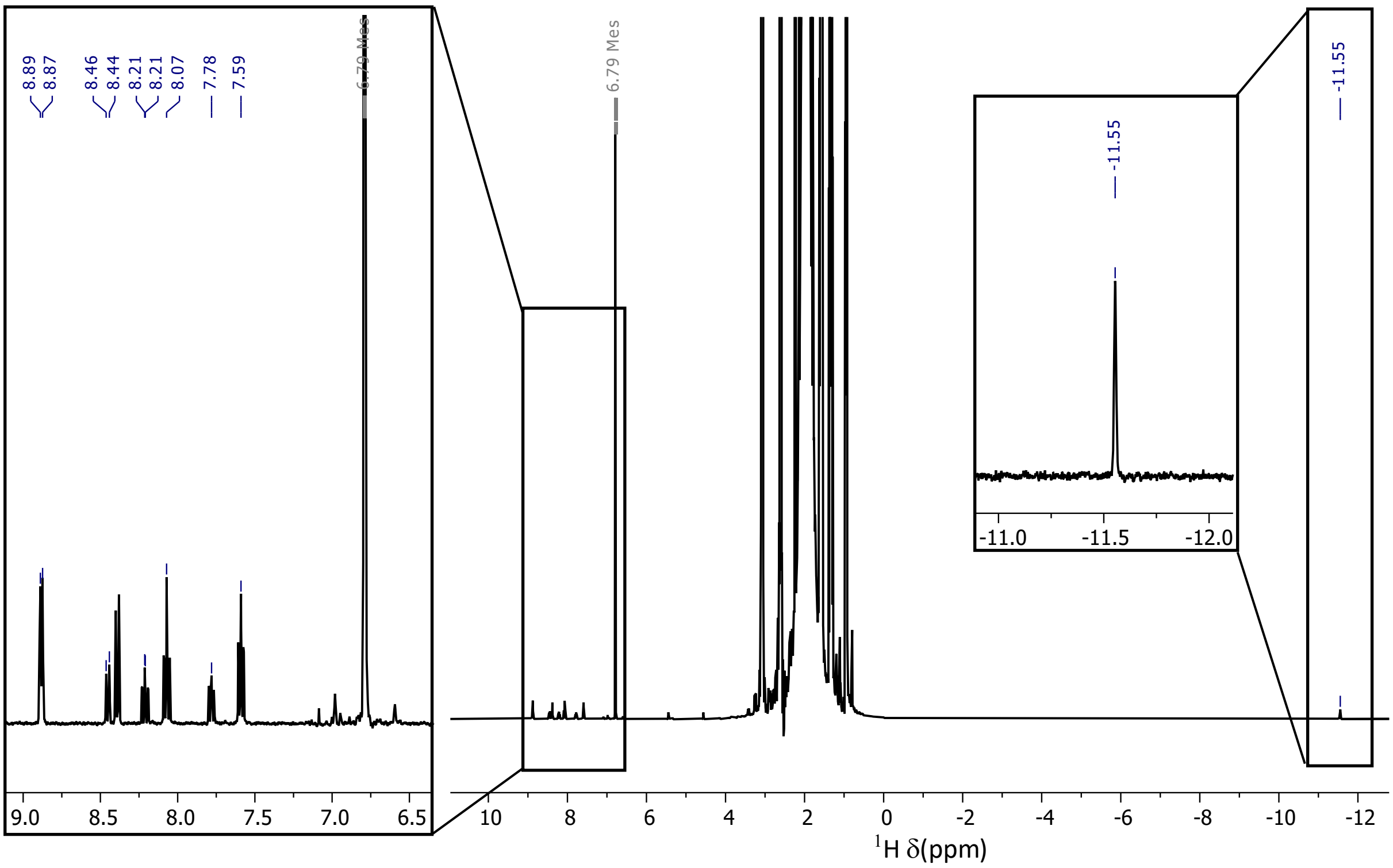

Figure S22. ^{1}H NMR spectra of post-electrolysis samples under CO_2 in a PEM cell with stirring (cell ii). The singlet at 6.79 ppm corresponds to the mesitylene internal standard (20 mM). Close-ups of the formyl and hydride spectral regions are shown. Under stirring, the formic acid peak is not observed unless a large excess of base (^{t}BuTMG) is added (Figure S23). Conditions: **Ir$_{Cat}$** (2 mM) in MeCN, 0.1 M $[nBu_4N][PF_6]$ at -2.0 V vs. $Fc^{+/0}$ under CO_2 atmosphere.

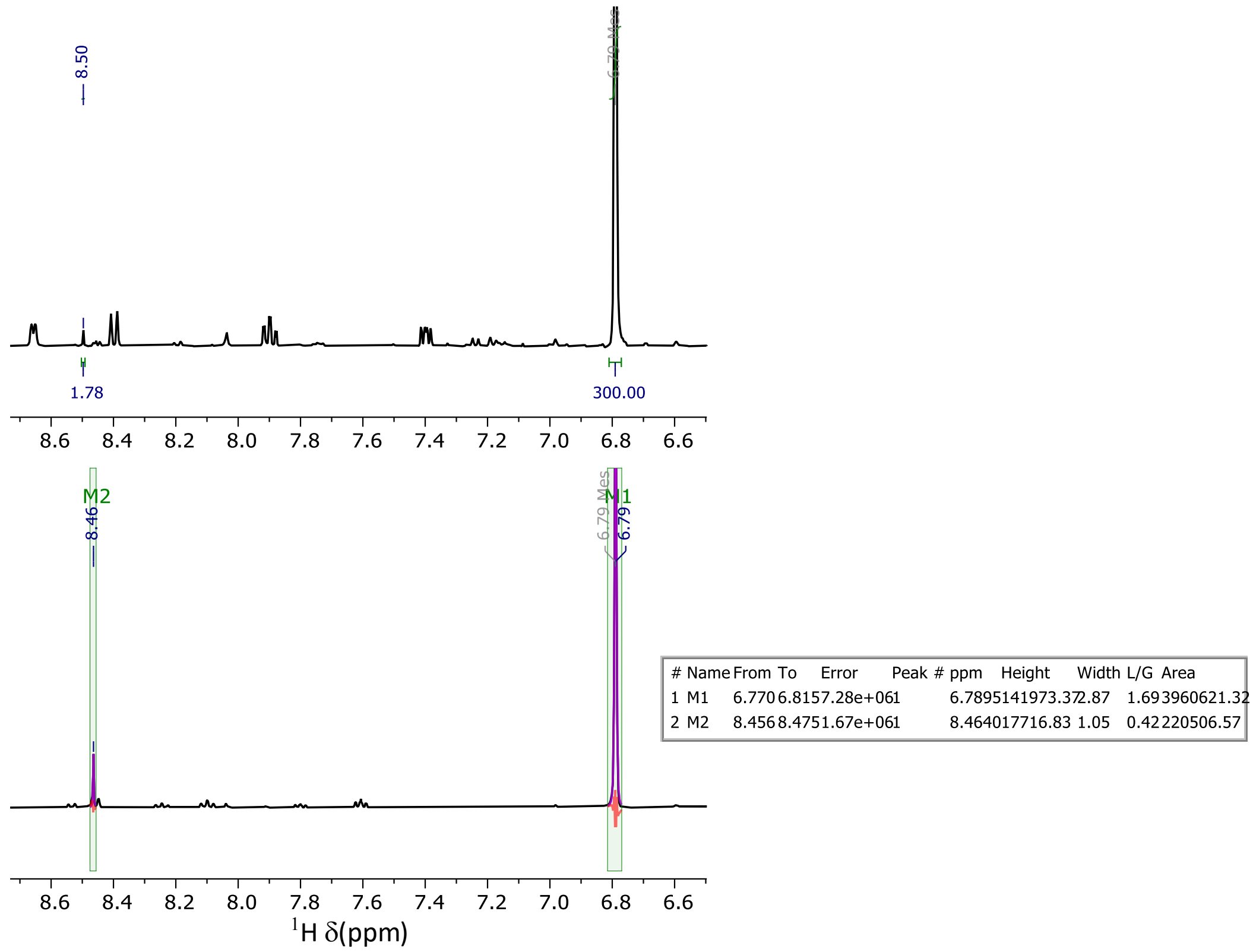

Figure S23. ^{1}H NMR spectra of base-treated post-electrolysis samples. The bottom spectrum corresponds to the sample obtained under non-stirring (Figure S21) and the top spectrum corresponds to the one obtained under stirring (Figure S22) both after addition of ^{t}BuTMG. The singlet at 6.79 ppm corresponds to the mesitylene internal standard (20 mM). In the bottom spectrum, the mesitylene and formate signals were fitted to account for partial signal overlap. Integration relative to the internal standard gave formate concentrations of 0.4 mM and 3.3 mM for the experiments performed under stirring and non-stirring conditions, corresponding to Faradaic efficiencies of 4.6% and 47%, respectively. Conditions: **Ir$_{\mathbf{Cat}}$** (2 mM) in MeCN, 0.1 M [nBu$_4$N][PF$_6$] at -2.0 V vs. Fc$^{+/0}$ under CO_2 atmosphere.

Table S5. CPE results with $\mathbf{Ir_{Cat}}$ and $\mathbf{Co_{Cat}}$ (2 mM) in MeCN with 1% H_2O. CPE with $\mathbf{Mn_{Cat}}$ (2 mM) were collected in MeCN with 1.5% TFE (instead of H_2O). Conditions: 0.1 M $[nBu_4N][PF_6]$ at -2.0 V vs. $Fc^{+/0}$ under CO_2 atmosphere.

Catalyst	Conditions	Q (C)	$[HCO_2(H)]$ (mM)	$FE_{HCO2(H)}$ (%)	δ_{obs} (ppm)	χ_{HCO2H}
$\mathbf{Ir_{Cat}}$	Glass frit	18.3	9.7	41	8.54	0.0
$\mathbf{Ir_{Cat}}$	Glass frit	19.1	10.8	44	8.54	0.0
$\mathbf{Ir_{Cat}}$	PEM, stirred	4.6	0.4	6	n.d.	n.d.
$\mathbf{Ir_{Cat}}$	PEM, non-stirred	5.5	3.3	47	8.08	0.96
$\mathbf{Ir_{Cat}}$	PEM, non-stirred	2.8	2.1	58	8.07	0.97
$\mathbf{Co_{Cat}}$	Glass frit	17.6	9.2	30	8.57	0.0
$\mathbf{Co_{Cat}}$	Glass frit	6.1	4.0	38	8.55	0.0
$\mathbf{Co_{Cat}}$	PEM, non-stirred	6.7	1.5	13	8.07	0.97
$\mathbf{Co_{Cat}}$	PEM, non-stirred	3.0	0.4	10	8.07	0.97
$\mathbf{Co_{Cat}}$	PEM, non-stirred[a]	1.0	0.4	29	8.06	0.97
$\mathbf{Co_{Cat}}$	PEM, non-stirred[a]	1.9	0.9	37	8.08	0.96
$\mathbf{Mn_{Cat}}$	Glass frit	6.0	0.4	4.6	8.57	n.d.
$\mathbf{Mn_{Cat}}$	PEM, non-stirred	1.9	0.6	24	8.08	0.96[b]
$\mathbf{Mn_{Cat}}$	PEM, non-stirred	5.0	1.9	29	8.07	0.97[b]

[a] Nafion 117 PEM was activated with Milli-Q water instead of 1 M $HCl_{(aq)}$.
[b] Molar fractions for $\mathbf{Mn_{Cat}}$ were approximated using Eq. S10, derived from data in MeCN with 1% H_2O (Figure S3B)."

Table S6. Summary of the Faradaic efficiencies after CPE with $\mathbf{Ir_{Cat}}$, $\mathbf{Co_{Cat}}$, and $\mathbf{Mn_{Cat}}$.

Catalyst	Conditions	FE (%)		
		$HCO_2(H)$	H_2	CO
$\mathbf{Ir_{Cat}}$	Glass frit	43±2	5.0±0.5	n.d.
$\mathbf{Ir_{Cat}}$	PEM, non-stirred	53±8	33±3	3.0±0.3
$\mathbf{Co_{Cat}}$	Glass frit	34±6	21±2	1.0±0.1
$\mathbf{Co_{Cat}}$	PEM, non-stirred	12±2 (33±6)[a]	65±7	n.d.
$\mathbf{Mn_{Cat}}$	Glass frit	4.6	n.d.	93±9
$\mathbf{Mn_{Cat}}$	PEM, non-stirred	27±4	1.0±0.2	58±6

[a] Nafion 117 PEM was activated with Milli-Q water instead of 1 M $HCl_{(aq)}$. [b] The values and associated errors for liquid products correspond to the average and standard deviation of two independent replicates. [c] The values for gaseous products correspond to single measurements, and an uncertainty of 10% was assigned to account for gas handling and sampling.

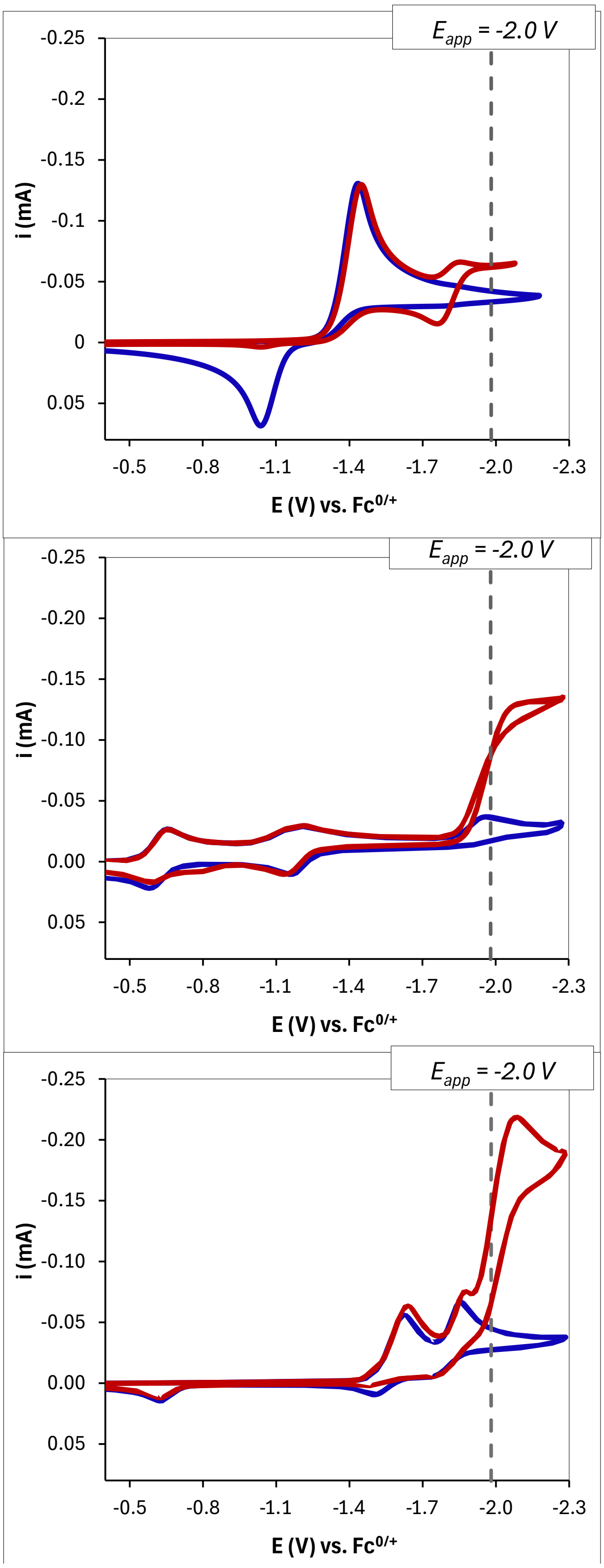

Figure S24. Cyclic voltammograms of catalysts (2 mM) **Ir$_{Cat}$** (top), **Co$_{Cat}$** (middle) and **Mn$_{Cat}$** (bottom) in MeCN 0.1 M [nBu$_4$N][PF$_6$] under N_2 (blue) and CO_2 (red) atmosphere in the presence of 1% H_2O (**Ir$_{Cat}$** and **Co$_{Cat}$**) or 1.5% TFE (**Mn$_{Cat}$**) over a glassy carbon disk working electrode (Ø = 0.3 cm).

6. Experimental study of the acidity gradients

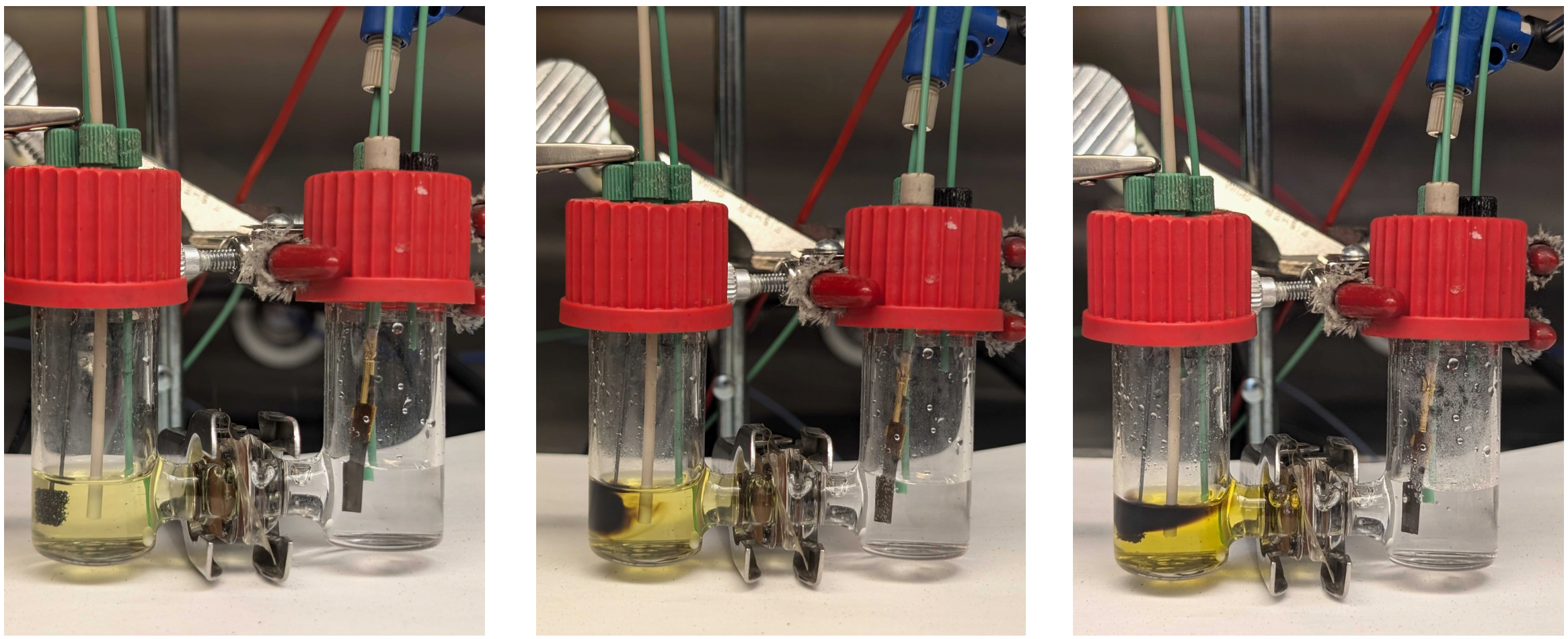

Figure S25. Non-stirred CO_2R PEM cell (cell ii) during CPE at -2.0 V vs. $Fc^{+/0}$ under N_2 atmosphere: 0.0, 0.28 and 1.25 C (from left to right). Electrolysis conditions: **[Ir–Cl]**$^+$ (2 mM) in MeCN 1% H_2O 0.1 M $[nBu_4N][PF_6]$ over RVC (cathode) separated from the anodic compartment (5 mM H_2SO_4 in H_2O over Ti foil) by a Nafion 117 membrane.

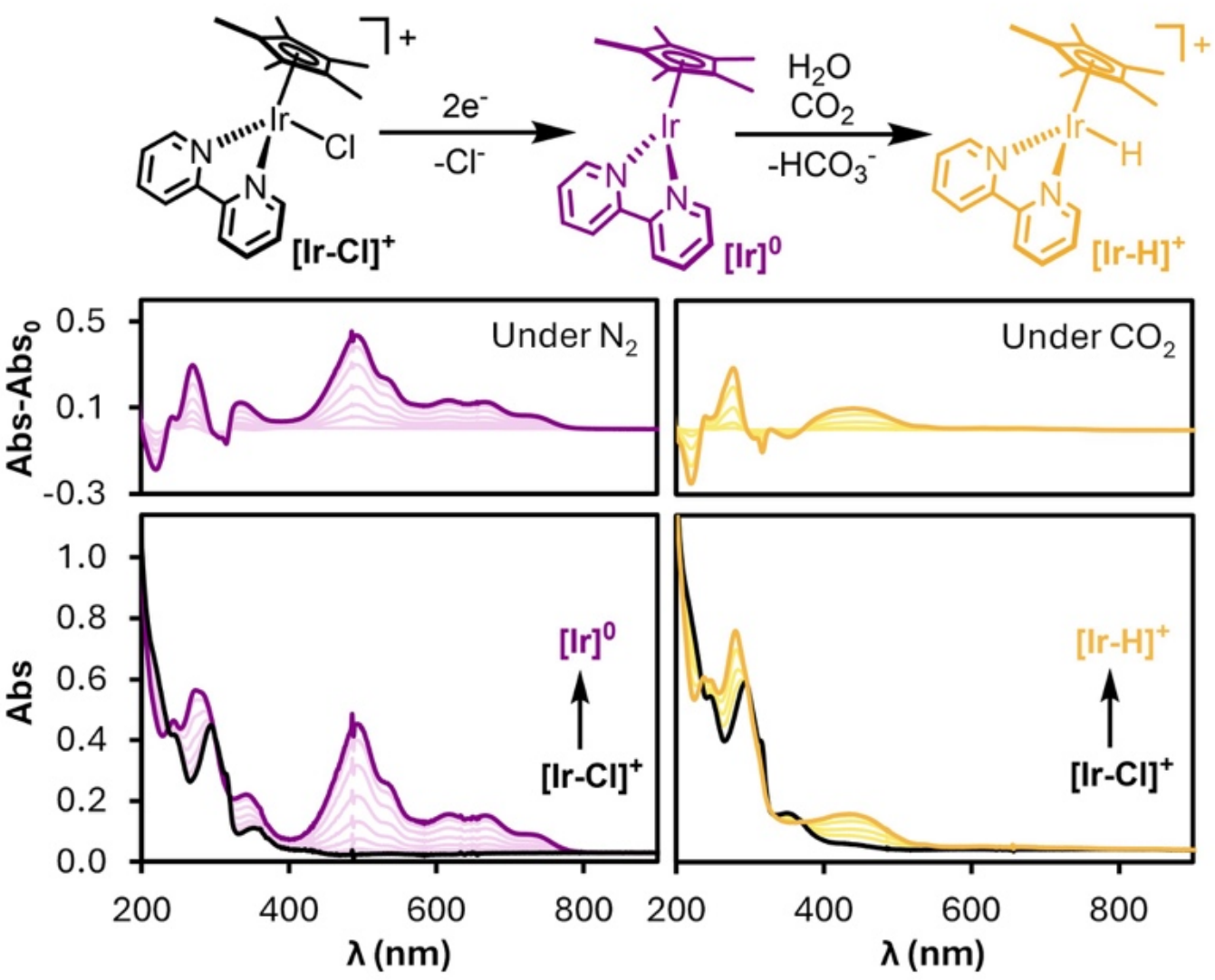

Figure S26. UV-vis SEC under N_2 (left) and CO_2 (right) with absolute (bottom) and differential spectra (top). In both cases, the starting spectrum corresponds to the black trace, intermediate spectra are shown in pink (N_2) and yellow (CO_2), final spectra are shown in purple (N_2) and orange (CO_2). SEC conditions: [**Ir**$_{Cat}$] = 2 mM in wet MeCN (1% H_2O) with 0.1 M $[nBu_4N][PF_6]$, linear scan voltammetry from 0 V to -1.3 V vs. Ag wire pseudo-reference at 2 mV/s.

Taking advantage of the well-defined thermochemistry of **Ir**$_{Cat}$ in MeCN solution, we can estimate pH differences across key regions in the system: the cathode-catholyte interface, the bulk solution, and the catholyte-PEM interface. Before electrolysis under N_2 atmosphere, H_2O ($pK_a \sim 41$ in MeCN) is the strongest acid available in solution.[8] Therefore, in the absence of CO_2, $[Ir]^0$ builds up at the working electrode surface. Since H_2O itself is not acidic enough to protonate $[Ir]^0$ a $\Delta pK_a > 18$ must develop between the cathode surface and the PEM in order to match the pK_a value of $[Ir\text{-}H]^+$ ($pK_a = 23.3$ in MeCN).[9] Furthermore, considering the reported effective hydricity value ($\Delta G_{H^-}(Cl^-) = 51.4$ kcal·mol^{-1}) of $[Ir–H]^+$,[10] the generation of an

effective pK_a < 18 at the PEM-catholyte interface is required protonation of the hydride to evolve H_2. This is supported by the thermochemical cycle in Figure S27:

$$[Ir{-}H]^+ + Cl^- \longrightarrow [Ir{-}Cl]^+ + H^- \qquad \Delta G_{H-}(Cl^-) = 51.4$$

$$H^+ + H^- \longrightarrow H_2 \qquad -\Delta G_{H2} = -76.0$$

$$[Base{-}H]^+ \longrightarrow [Base] + H^+ \qquad 1.364 \cdot pK_a$$

$$[Ir{-}H]^+ + [Base{-}H]^+ \longrightarrow [Ir{-}Cl]^+ + H_2 + [Base] \qquad \Delta G_r = 0.0\ kcal \cdot mol^{-1}$$

Figure S27. Thermochemical cycle for the determination of pH boundary for HER from $[Ir{-}H]^+$. At the thermodynamic limit for HER (ΔG_r = 0.0 kcal/mol), the corresponding acidity threshold is pK_a = 18.

Under CO_2, the apparent pK_a ($CO_2 + H_2O$) in MeCN was previously estimated as 23.4,[11] enabling equilibrium between $[Ir]^0$ and $[Ir{-}H]^+$ to be established near the electrode surface even in the absence of a proton gradient. Therefore, without a PEM, formate would be generated at 0.51 V overpotential (η_{HCO2-}). However, product analysis after CPE reveals a 96:4 molar ratio of HCO_2H to HCO_2^-, indicating that the pH decreases significantly during electrolysis. Applying the Henderson-Hasselbach equation:

$$pH = pK_a + \log\left(\frac{[A^-]}{[HA]}\right) = 20.9 + \log\left(\frac{0.04}{0.96}\right) = 19.5 \qquad (S14)$$

This analysis suggests a pH drop of approximately 4 units during electrolysis, consistent with the buildup of $[HCO_2H]$ in the bulk solution. At such low bulk pH, electrochemical HER is expected to dominate over CO_2R. However, the presence of a proton gradient across the catholyte maintains a more basic local pH at the cathode-catholyte interface. Because the bulk product is HCO_2H, the system is operating under condition where the local effective pK_a lies within the *formate regime* in the thermodynamic potential-pK_a diagram (main text Figure 5B).

Table S7. Summary of pK_a for selected catalyst and their corresponding metal hydride intermediates.

Catalyst	Intermediate	pK_a	Source
**Ir_{Cat}**	$[Ir{-}H]^+$	23.3	SI ref. 9, 10
**Co_{Cat}**	$[Co{-}H]^+$	23.4	SI ref. 3
**Mn_{Cat}**	$[Mn{-}H]^0$	28.8	Main text ref. 52

7. COMSOL Multi-scale Modeling

7.1. Methodology

A time-dependent 1D model was developed to simulate the dynamics of a plane connecting the cathode and anode through the membrane across a typical electrochemical H-cell as represented schematically in Figure S28. During the reaction, the catholyte is unstirred so that a pH gradient can form across the cathode compartment of the cell. Thus, the dominant mechanisms of mass transport are diffusion and migration. Migration was not modeled because the high electrolyte concentration will minimize the impact. Convection was not modeled in this unstirred cell. As discussed above, crossover experiments indicated very little diffusion of neutral species (formic acid and acetonitrile) from the cathode to the anode, in opposition to the direction of the ionic current. The catalyst molecules can diffuse and migrate in the catholyte, but they are largely excluded from the Nafion membrane by the flux of protons from the anolyte and by migration of positively charged $[Ir–Cl]^+$ and $[Ir–H]^+$ towards the cathode. Migrating from the anode to the cathode, H^+ is considered the dominant charge carrier.

The modeling approach is summarized below:

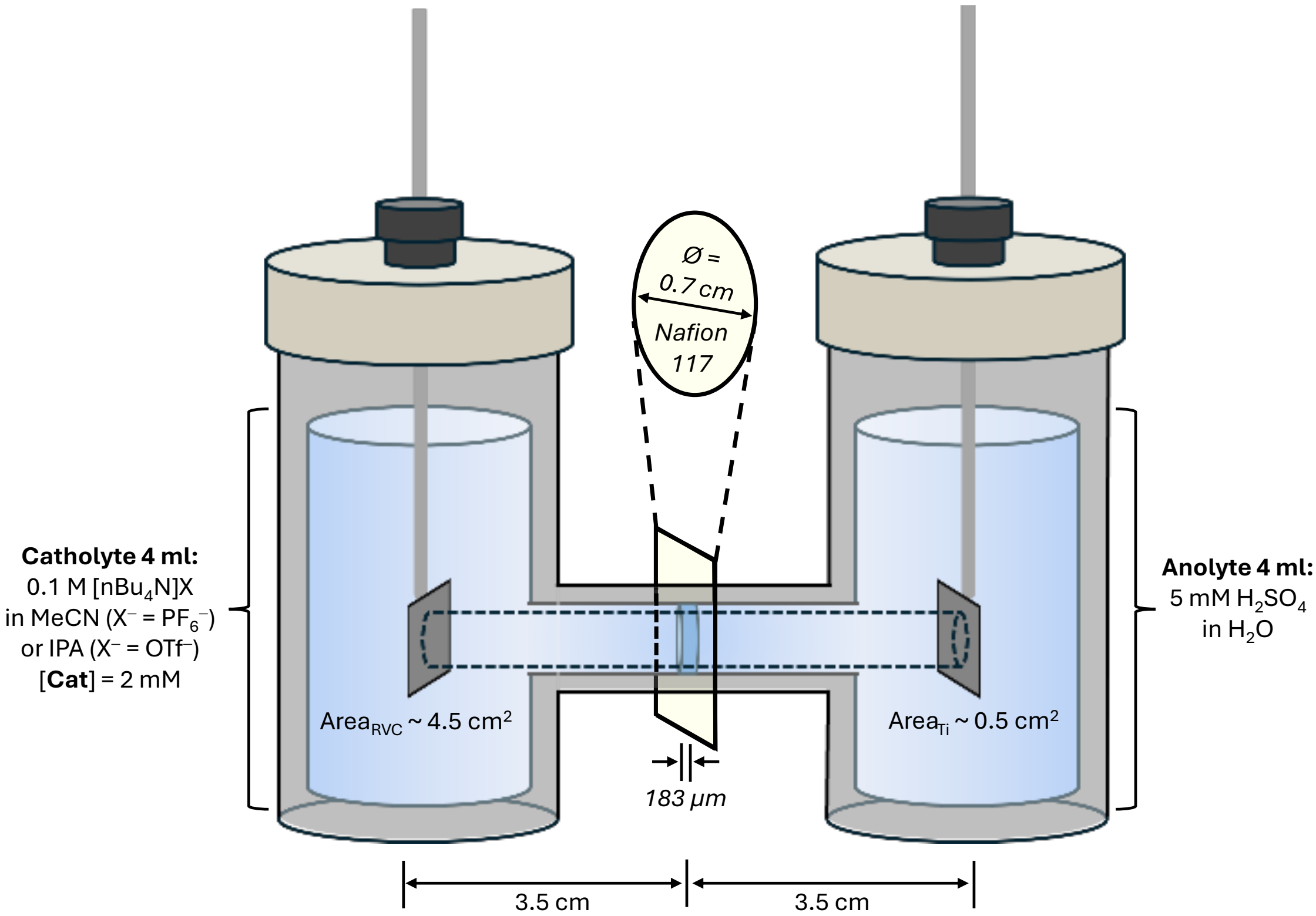

Figure S28. Simplified diagram of experimental H-cell configuration, with the 1D simulated domain expressed as a dotted cylinder.

Geometric Domains

The model effectively considers a 3D domain where the governing equations and physics are only non-uniform in one axis. Thus, the simulated domain can be considered a cylinder with a defined cross-sectional area equal to the membrane and spans the length of the H-cell from

cathode to anode (Figure S28). The geometric dimensions of the catholyte, membrane, and anolyte domains are taken from experimental measurements (Table S8).

Governing Equations

Electrolyte Domain

Species flux (N_i) is calculated using the Nernst-Planck equation (eq S15) and only considers transport through diffusion and migration to reflect the lack of stirring in the experiments.

$$N_i = \underbrace{-D_i \nabla c_i}_{Diffusion} - \underbrace{z_i u_{m,i} F c_i \nabla \phi_{el}}_{Migration} \tag{S15}$$

For each term in eq S15, each individual species is denoted by subscript i that applies to all aqueous species (H^+, OH^-, CO_2, HCO_2^-, HCO_2H, HSO_4^-, SO_4^{2-}, TBA^+, OTf^-, Cl^-, **Ir–Cl**$^+$, **Ir**^{0}, **Ir–H**$^+$, X^-, HCl). The first term of eq S15 describes diffusive flux, where D_i is the diffusion constant of species i, and ∇c_i is the gradient of concentration of species i ($\frac{dc_i}{dx}$ in 1D). The second term describes electric migration, where z_i represents charge, $u_{m,i}$ is mobility, F is Faraday's constant, and ϕ_{el} is electrolyte potential.

The mobility of species i, $u_{m,i}$, is defined by the Nernst-Einstein relation (eq S16):

$$u_{m,i} = \frac{D_i}{RT} \tag{S16}$$

Charge is conserved across the H-cell, where D_i is the diffusion coefficient, R is the gas constant, and T is temperature, with electroneutrality imposed by the following condition:

$$\sum_i z_i c_i = 0 \tag{S17}$$

The electrolyte current density is defined with the following equation:

$$i_{l,e} = F \sum_i z_i \left(-D_i \nabla c_i - z_i u_{m,i} F c_i \nabla \phi_{el}\right) \tag{S18}$$

Catholyte Compartment

The following reactions are defined in the catholyte:

$$[IrH]^+ \underset{k_{-1}}{\overset{k_1}{\longleftrightarrow}} Ir^0 + H^+ \; (K_1 = 10^{-23.3}) \tag{S19}$$

$$HCOOH \underset{k_{-2}}{\overset{k_2}{\longleftrightarrow}} HCOO^- + H^+ \; (K_2 = 10^{-20.9}) \tag{S20}$$

$$HCl \longleftrightarrow Cl^- + H^+ \; (K_3 = 10^{-10.3}) \tag{S21}$$

$$HX \longleftrightarrow X^- + H^+ \; (K_4 = 10^{-pKa}) \tag{S22}$$

where the pK_a of S21 varies depending on the operating conditions of the experimental setup. Under CO_2 conditions, the pK_a is set to 23.4. Under N_2 conditions, the pK_a is set to 30 as a first approximation for the known higher pK_a without CO_2, and to ensure reliable convergence.

The reactions in eq S21-22 are assumed to occur very fast and are thus treated as equilibrium reactions such that the equilibrium conditions are met at all times and positions. However, the

protonation reactions S19-20 have species set at zero concentration initially and are thus modeled as quasi-equilibrium reactions (forward and reverse rate constant).

Since HCl in MeCN has a pK_a of 10.3, the modeled proton carrier H^+ is taken to be HCl in the catholyte.[12]

For each homogeneous quasi-equilibrium reaction, the rates R_i (units of M/s) of participating species in the catholyte can be expressed with the following kinetic expressions:

$$R_{Ir^0} = k_1[IrH]^+ - k_{-1}[Ir^0][H^+] \quad \text{(S23)}$$

$$R_{[IrH]^+} = k_{-1}[Ir^0][H^+] - k_1[IrH]^+ \quad \text{(S24)}$$

$$R_H = k_1[IrH]^+ - k_{-1}[Ir^0][H^+] + k_2[HCOOH] - k_{-2}[H^+][HCOO^-] \quad \text{(S25)}$$

$$R_{HCOO-} = k_2[HCOOH] - k_{-2}[H^+][HCOO^-] \quad \text{(S26)}$$

$$R_{HCOOH} = -k_2[HCOOH] + k_{-2}[H^+][HCOO^-] \quad \text{(S27)}$$

Anolyte Compartment

The following equilibrium reactions are defined to consider sulfuric acid dissociation in water:

$$HSO_4{}^- \longleftrightarrow H^+ + SO_4^{-2} \; (K_3 = 0.012) \quad \text{(S28)}$$

$$H_2O \longleftrightarrow H^+ + OH^- \; (K_w = 10^{-14}) \quad \text{(S29)}$$

Membrane Domain

To simulate the behavior of the PEM, "free" protons are shuttled through the Nafion 117 membrane from the anolyte chamber to the catholyte chamber. The membrane is simulated as a region of fixed space-charge to represent the negative sulfonic ions in the polymer matrix; thus, only protons are present in this domain at a fixed concentration $[H^+]_m$. The membrane current density can then be described by Ohm's law:

$$i_{l,m} = -\sigma_{l,m} \nabla \phi_m \quad \text{(S30)}$$

where $\sigma_{l,m}$ is the proton conductivity of the membrane and $\nabla \phi_m$ is the gradient of the membrane potential.

Boundary Conditions:

Membrane-Electrolyte Interface

To calculate the potential shift associated with the difference in concentration of protons in the membrane and in the electrolyte, Donnan potentials are implemented such that:

$$\Delta\phi = \phi_{el} - \phi_m = -\frac{RT}{F}\ln\left(\frac{[H^+]_{el}}{[H^+]_m}\right) \quad \text{(S31)}$$

The normal flux of protons from the membrane boundary is finally summarized with the following expression: $n \cdot N_{H^+} = n \cdot \frac{i_m}{F}$

Cathode/Anode Surfaces

Cathode Kinetics

At the cathode surface, the following reactions are defined:

$$[IrCl]^{+} + 2e^{-} \rightarrow Ir^{0} + Cl^{-} \tag{S32}$$

$$H^{+} + [IrH]^{+} + 2e^{-} \rightarrow Ir^{0} + H_2 \tag{S33}$$

The electron-transfer kinetics are initially described using the modified Butler-Volmer relation to account for the effects of mass-transport:

$$j_i = j_{0,i}\left[C_R\, e^{\frac{\alpha_a nF}{RT}\eta} - C_O\, e^{\frac{-\alpha_c nF}{RT}\eta} \right] \tag{S34}$$

where $j_{0,i}$ is the exchange-current density, α_x is the cathodic/anodic transfer coefficient, n is the number of electrons, and C_x is the unitless concentration-dependence of the reduced/oxidized species.

Since cathodic potentials away from equilibrium are used experimentally, the expression can be further reduced to concentration-dependent Tafel kinetics:

$$j_i = -j_{0,i}\left(C_O\, e^{\frac{-\alpha_c nF}{RT}\eta} \right) \tag{S35}$$

Under a fixed potential, the exponential term can be treated as a constant and then combined with the exchange current-density pre-factor to give the final general kinetic expression:

$$j_i = \ k_i C_O \tag{S6}$$

By considering assuming a first order dependence in [IrCl] for reaction (S20), we obtain the following expression.

$$j_{IrCl} = k_{IrCl}\left(\frac{[IrCl]}{[IrCl]_{tot}} \right) \tag{S37}$$

Anode Kinetics

The anode is coupled to the cathode and has an equivalent current density. This is achieved through the imposed electroneutrality, which dictates the electrolyte current density and adjusts the overpoential term accordingly to satisfy the condition. At the anode, the oxygen evolution reaction is defined to balance the electrochemical reactions at the cathode:

$$2H_2O \rightarrow 4H^{+} + 4e^{-} + O_2 \tag{S38}$$

Water is modeled implicitly in the model by considering the known equilibrium reaction with H^+ and OH^-; thus, the concentration dependence is negligible when formulating the kinetic expression. Since the experiment is operating at cathodic potentials away from equilibrium, and the cathode is limiting the current passed through the cell, the OER is modeled using a linearized Butler-Volmer relation:

$$j_{OER} = j_{0,OER}\left(\frac{F}{RT}\eta_{OER} \right) \tag{S39}$$

$$\eta_{OER} = \phi_s - \phi_{el} - \left(U_{0,OER} - \frac{2.303RT}{F}pH\right) \quad \text{(S40)}$$

where ϕ_s is the applied potential at the electrode, ϕ_{el} is the electrolyte potential, $U_{0,OER}$ is the equilibrium potential, and pH is defined as $-\log_{10}[H^+]$ in the anolyte.

Model of Electrocatalytic CO_2 Reduction Conditions

For CO_2 reduction, the model assumes that the concentration of supporting electrolyte is sufficient to screen charges and ignores electric field effects. Furthermore, we assume the flux of protons from the membrane matches the electrochemical current density. The species considered in this model are CO_2, HX, H^+, X^-, $HCOO^-$, and $HCOOH$. Parameters for initial concentrations, diffusion, and homogeneous rate constants remain the same as in Table S11.

The simplified model exactly translates the time-dependent experimental current density of Figure S14 to the flux of protons passing through the membrane. The total experimental current was partitioned to each reaction in accordance to their Faradaic efficiency. To sustain the experimental current, the species HX was set to an initial 500 mM and the species distribution of the catalyst species is not considered. Instead, the reactions defined at the cathode are replaced with the following expressions:

$$CO_2 + HX + 2e^- \leftrightarrow HCOO^- + X^- \quad \text{(S41)}$$

$$2HX + 2e^- \leftrightarrow H_2 + 2X^- \quad \text{(S42)}$$

The following homogeneous reactions are defined for the simplified model and set up the same way as in the previous model (see equations S20 and S22).

The concentrations of $(HCOO^- + HCOOH)$ at $t = 120$ min was integrated and normalized to the 4 ml volume of the experimental catholyte to obtain a total concentration of 3.9 mM. This value is of the same order of magnitude as the experimentally measured concentration.

A sensitivity analysis was performed for the simplified model, and concentration profiles for product species are shown in Figure S30. To perform the sensitivity analysis at longer timescales, an additional condition was needed to sustain the extrapolated experimental current from Figure S18. An additional reservoir of HX was implicitly included to represent the additional source of HX species available outside the modeled domain environment (Figure S30).

$$HX_{env} \overset{k_{mt}}{\longleftrightarrow} HX \quad \text{(S43)}$$

where the k_{mt} is set to $10^8 s^{-1}$ to maintain the current density.

7.2. Parameters for COMSOL model

Table S8. Geometric parameters of the electrochemical cell.

Parameter	Value	Units	Source	Description
Catholyte_length	3.5	cm	Experimental	*Length of catholyte compartment*
Anolyte_length	3.5	cm	Experimental	*Length of anolyte compartment*
NF117_length	183	um	Experimental	*Thickness of Nafion 117 membrane*
cross_area	0.38485	cm²	Experimental	$\pi\left(\frac{0.7cm}{2}\right)^2$ *The 1D simulation assumes a uniform cross-sectional surface-area throughout the model, taken to be the width of the membrane.*

Table S9. Diffusion Coefficients for Solutes at 298 K.

Parameter	Value	Units	Source	Notes
D_{CO2} in MeCN	3.1×10^{-9}	m² s^{-1}	13	
D_{HCOOH} in MeCN	3.77×10^{-9}	m² s^{-1}	14	*Assumed to be equal to the value in acetone*
D_{HCOO^-} in MeCN	3.77×10^{-9}	m² s^{-1}	14	*Assumed equal to HCOOH*
D_{Ir-H} in MeCN	5.5×10^{-11}	m² s^{-1}	15	
D_{Ir^0} in MeCN	5.5×10^{-11}	m² s^{-1}	15	
D_{Ir-Cl} in MeCN	5.5×10^{-11}	m² s^{-1}	15	
D_{TBA^+} in MeCN	1.64×10^{-9}	m² s^{-1}	14	
D_{OTf^-} in MeCN	1.64×10^{-9}	m² s^{-1}	Assumed	*Assumed equal to TBA$^+$*
D_{HX} in MeCN	1×10^{-9}	m² s^{-1}	Assumed	*Order of magnitude approximation*
D_{X^-} in MeCN	1×10^{-9}	m²/s	Assumed	*Order of magnitude approximation*
D_{HCl} in MeCN	1×10^{-9}	m² s^{-1}	Assumed	*Order of magnitude approximation*
D_{Cl^-} in MeCN	1×10^{-9}	m² s^{-1}	Assumed	*Order of magnitude approximation*
D_{H^+} in MeCN	2×10^{-9}	m² s^{-1}	16	
D_{H^+} in H_2O	9.311×10^{-9}	m² s^{-1}	17	
D_{OH^-} in H_2O	5.273×10^{-9}	m² s^{-1}	17	
$D_{HSO_4^-}$ in H_2O	1.385×10^{-9}	m² s^{-1}	17	
$D_{SO_4^{2-}}$ in H_2O	1.065×10^{-9}	m² s^{-1}	17	
$[CO_2]_{bulk}$	280	mM	18	*Concentration of CO_2 in MeCN under 1 atm gas*
$[IrCl]_{tot}$	2	mM	Experimental	*Maximum amount of [IrCl] based on the total amount of Ir added to the cell.*
$[Ir^0]$	2	mM	Experimental	*Maximum amount of [Ir^0] based on the total amount of Ir added to the cell.*

$[IrH^+]_{tot}$	2	mM	Experimental	*Maximum amount of [IrH$^+$] based on the total amount of Ir added to the cell.*
$[H^+]_{bulk}$	Init_H	mM	Assumed	

Table S10. Membrane Parameters

Parameter	Value	Units	Source	Description
$[H^+]_m$	1000	mM	[19]	*Fixed charge concentration*
$\sigma_{l,m}$	12	S m^{-1}	[19]	*Proton conductivity of PEM*

Table S11. Initial Concentration Values (at $t = 0$) in catholyte

Parameter	Value (CO_2)	Value (N_2)	Units	Source	Description
Init_CO_2	280	0	mM	[20]	*1% H_2O in acetonitrile at 25ºC at 1 atm P_{CO2}*
Init_HX	500	555	mM	Assumed	*Assumed concentration of acid in MeCN*
Init_X	6.31×10^{-10}	7.449×10^{-13}	mM	Calculated	*Calculated from HX and HCl equilibrium*
Init_H	6.31×10^{-10}	7.449×10^{-13}	mM	Calculated	*Calculated from HX and HCl equilibrium*
Init_HCl	0.0252	2.973×10^{-5}	mM	Calculated	*Calculated from HX and HCl equilibrium*
Init_IrCl	2		mM	Experimental	
Init_$HCOO^-$	0		mM	Experimental	
Init_HCOOH	0		mM	Experimental	
Init_IrH	0		mM	Experimental	
Init_Ir^0	0		mM	Experimental	
Init_TBA	100		mM	Experimental	
Init_OTf	100		mM	Experimental	
Init_Cl	2		mM	Experimental	

Table S12. Initial Concentration Values (at $t = 0$) in anolyte

Parameter	Value (CO_2)	Units	Reference	Description
Init_HSO4	2	mM	Calculated	*Calculated equilibrium value for 5mM H_2SO_4 in H_2O (K_{eq}=0.012)*
Init_SO4	3	mM	Calculated	*Calculated equilibrium value for 5mM H_2SO_4 in H_2O (K_{eq}=0.012)*
Init_OH	1.25×10^{-11}	mM	Calculated	*Calculated equilibrium value for 5mM H_2SO_4 in H_2O (K_{eq}=0.012)*

Table S13. Homo/hetergeneous Kinetic Rate Parameters

Parameter	Value	Units	Reference	Description
E_{app}	-1.63<br>-1.433	V vs. Ag/AgCl<br>V vs. SHE	Experimental	*Applied voltage at the cathode*
j_tot	5.412×10^{-2}	mA/cm²	Experimental	$\left(\frac{14C/16hr}{4.5\ cm^2}\right)$<br>*Steady current density over 16hr*
k_{IrCl}	2.16×10^{-2}	mA cm^{-2}	Calculated	$j_{tot} \times 40\%$ *FE (experimental)*

k_{CO2}	2.16×10^{-2}	mA cm^{-2}	Calculated	*Set to k_{IrCl} to reflect product formation from CO2R*
k_H	3.24×10^{-2}	mA cm^{-2}	Calculated	*$j_{tot} \times 60\%$ FE (experimental)*
K_1	$10^{-23.3}$	M	Experimental	*Equilibrium rate constant for eq S22, calculated from measured pK_a*
k_1	$K_1 \times k_{-1}$	s^{-1}	Calculated	*Forward rate constant for eq S22*
k_{-1}	2×10^{10}	M^{-1} s^{-1}	Assumed	*Reverse rate constant for eq S22, assumed to be diffusion limited*
K_2	$10^{-20.9}$	M	[21]	*Equilibrium rate constant for eq S20, calculated from reported pK_a measured in acetonitrile*
k_2	$K_2 \times k_{-2}$	s^{-1}	Calculated	*Forward rate constant for eq S20*
k_{-2}	10^6	M^{-1} s^{-1}	[22]	*Reverse rate constant for eq S20, approximated based on benzoate data in acetonitrile*
$j_{0,OER}$	10	A m^{-2}	[19]	*Exchange current density pre-factor for OER described by eq S22 from an aqueous flow-cell performing CO_2R to CO*
$U_{0,OER}$	1.23	V vs. RHE	[19]	-

7.3. COMSOL simulation results

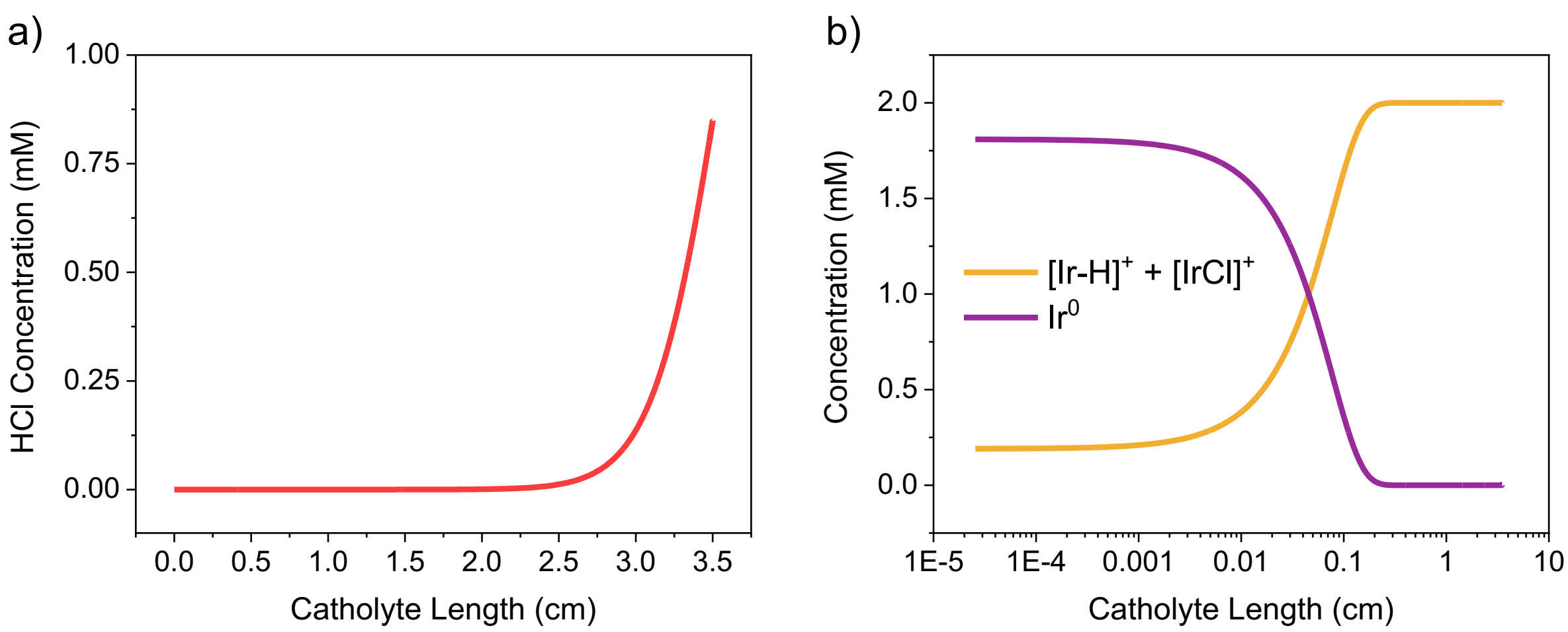

Figure S29. Concentration profiles of species in the catholyte obtained from the COMSOL model in the absence of CO_2 at t = 120 min for a) HCl^- and b) Ir catalyst species, where x = 0 cm designates the cathode and x = 3.5 cm corresponds to the membrane boundary.

Sensitivity analysis

Under the standard experimental conditions, HCO_2^- and H^+ develop opposing concentration gradients from the cathode and membrane, respectively, and HCO_2H concentration maximizes at the region where these gradients intersect (Figure S30). Sensitivity analysis reveals that the region where HCO_2H forms is strongly influenced by mass transport, cell length, and electrolysis time. Increasing the diffusion coefficients of all species, to simulate enhanced mass transport phenomena (e.g. from convection or stirring) broadens the concentration profiles and

promotes their overlap, resulting in a wider HCO_2H distribution. Similarly, decreasing the cathode-membrane distance increases width of the HCO_2H area, bringing a higher concentration of HCO_2H closer to the cathode. In contrast, increasing the catholyte length spatially separates the regions dominated by HCO_2^- and H^+, respectively, suppressing HCO_2H, as the HCO_2^- and H^+ gradients do not overlap. These results indicate that both diffusion and cathode-membrane distance control the location and extent of the acidity gradient. A time-dependence using the standard cell length and diffusion coefficients shows that formic acid gradually builds up near the cathode and membrane during the electrolysis. For these longer studies, the model took into account the continued acidification of the solution by CO_2, with dissolved HX being resupplied from a reservoir of HX(g) (eq. S43).

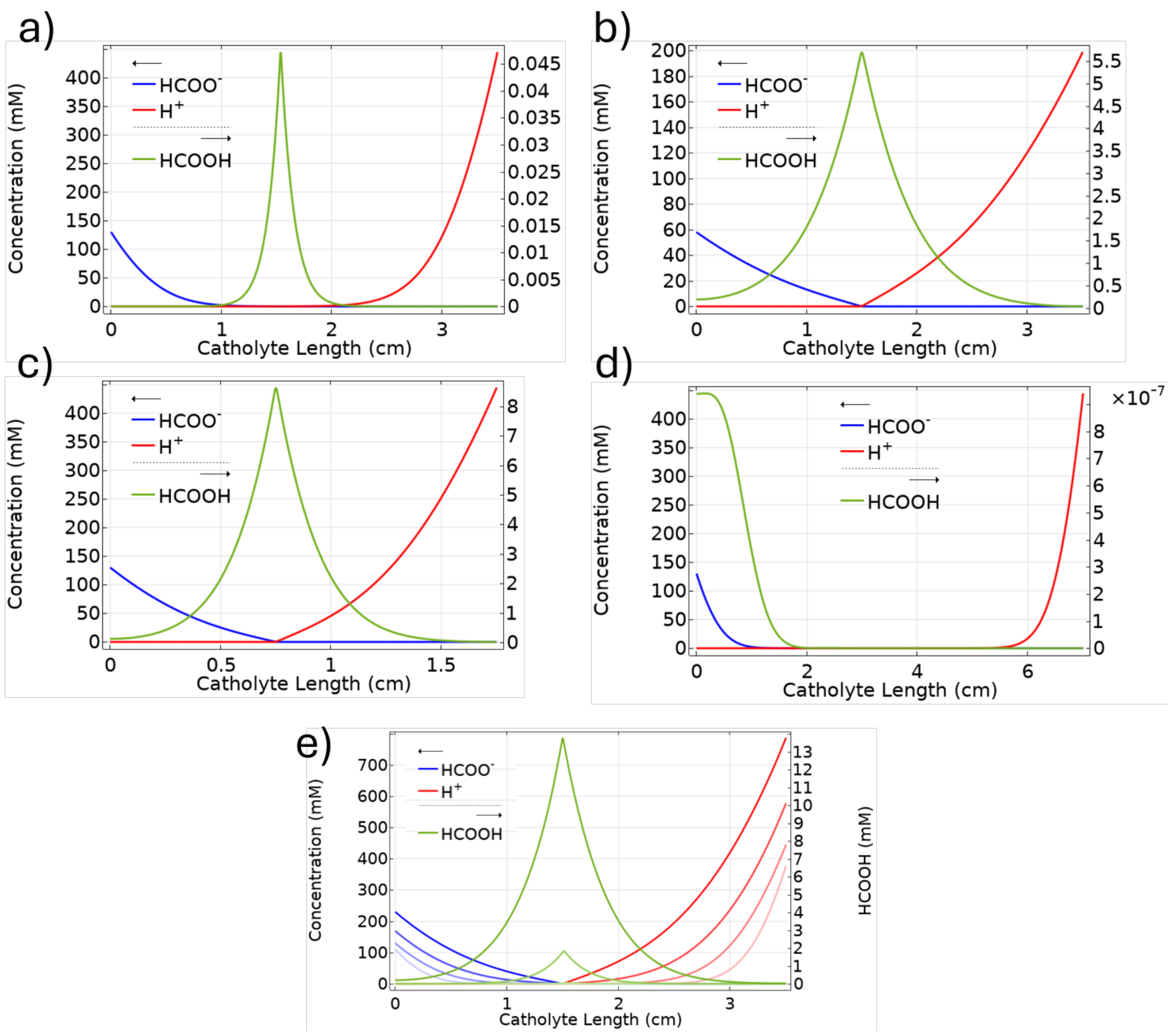

Figure S30. Sensitivity analysis for the electrocatalytic CO_2 reduction model. Concentration profiles of species in the catholyte at t = 120 min for $HCOO^-$, H^+, and HCOOH, where x = 0 cm designates the cathode surface for a) standard conditions, b) 5x diffusion coefficient for all species, c) 1.75 cm catholyte length, d) 7 cm catholyte length, and e) time evolution from 1, 2, 4, and 8 hours (from lighter to darker, respectively). The left y-axis represents the concentration of $HCOO^-$ and H^+, while the right y-axis represents the concentration of HCOOH (all reported in mM). Note: in a-d, integration of the $HCOO^-$ and HCOOH concentration profiles and normalization to the total catholyte volume (4 mL) yields a total $HCOO^-$ + HCOOH concentration of 3.9 mM.

8. References

(1) Dadci, L.; Elias, H.; Frey, U.; Hoernig, A.; Koelle, U.; Merbach, A. E.; Paulus, H.; Schneider, J. S. .pi.-Arene Aqua Complexes of Cobalt, Rhodium, Iridium, and Ruthenium: Preparation, Structure, and Kinetics of Water Exchange and Water Substitution. *Inorganic Chemistry* **1995**, *34* (1), 306-315. DOI: 10.1021/ic00105a048.

(2) Bourrez, M.; Molton, F.; Chardon-Noblat, S.; Deronzier, A. [Mn(bipyridyl)(CO)3Br]: An Abundant Metal Carbonyl Complex as Efficient Electrocatalyst for CO2 Reduction. *Angewandte Chemie International Edition* **2011**, *50* (42), 9903-9906. DOI: https://doi.org/10.1002/anie.201103616 (acccessed 2026/03/22).

(3) Montgomery, C. L.; Ertem, M. Z.; Chevalier, L.; Dempsey, J. L. Circumventing Kinetic Barriers to Metal Hydride Formation with Metal–Ligand Cooperativity. *Journal of the American Chemical Society* **2024**, *146* (44), 30020-30032. DOI: 10.1021/jacs.4c01716.

(4) Murata, T.; Hiyoshi, M.; Ratanasak, M.; Hasegawa, J.-y.; Ema, T. Synthesis of silyl formates, formamides, and aldehydes via solvent-free organocatalytic hydrosilylation of CO2. *Chemical Communications* **2020**, *56* (43), 5783-5786, 10.1039/D0CC01371D. DOI: 10.1039/D0CC01371D.

(5) Krejčik, M.; Daněk, M.; Hartl, F. Simple construction of an infrared optically transparent thin-layer electrochemical cell: Applications to the redox reactions of ferrocene, Mn2(CO)10 and Mn(CO)3(3,5-di-t-butyl-catecholate)−. *Journal of Electroanalytical Chemistry and Interfacial Electrochemistry* **1991**, *317* (1), 179-187. DOI: https://doi.org/10.1016/0022-0728(91)85012-E.

(6) Oliveira, D. R.; da Costa, E. T.; Schenberg, L. A.; Ducati, L. C.; do Lago, C. L. 13C NMR as an analytical tool for the detection of carbonic acid and pKa determination. *Magnetic Resonance in Chemistry* **2024**, *62* (2), 114-120. DOI: https://doi.org/10.1002/mrc.5430 (acccessed 2026/03/22).

(7) Madsen, M. R.; Rønne, M. H.; Heuschen, M.; Golo, D.; Ahlquist, M. S. G.; Skrydstrup, T.; Pedersen, S. U.; Daasbjerg, K. Promoting Selective Generation of Formic Acid from CO2 Using Mn(bpy)(CO)3Br as Electrocatalyst and Triethylamine/Isopropanol as Additives. *Journal of the American Chemical Society* **2021**, *143* (48), 20491-20500. DOI: 10.1021/jacs.1c10805.

(8) Cox, B. G. *Acids and Bases: Solvent Effects on Acid-Base Strength*; Oxford University Press, 2013. DOI: 10.1093/acprof:oso/9780199670512.001.0001.

(9) Barrett, S. M.; Pitman, C. L.; Walden, A. G.; Miller, A. J. M. Photoswitchable Hydride Transfer from Iridium to 1-Methylnicotinamide Rationalized by Thermochemical Cycles. *Journal of the American Chemical Society* **2014**, *136* (42), 14718-14721. DOI: 10.1021/ja508762g.

(10) Brereton, K. R.; Jadrich, C. N.; Stratakes, B. M.; Miller, A. J. M. Thermodynamic Hydricity across Solvents: Subtle Electronic Effects and Striking Ligation Effects in Iridium Hydrides. *Organometallics* **2019**, *38* (16), 3104-3110. DOI: 10.1021/acs.organomet.9b00278.

(11) Matsubara, Y.; Grills, D. C.; Kuwahara, Y. Thermodynamic Aspects of Electrocatalytic CO2 Reduction in Acetonitrile and with an Ionic Liquid as Solvent or Electrolyte. *ACS Catalysis* **2015**, *5* (11), 6440-6452. DOI: 10.1021/acscatal.5b00656.

(12) Raamat, E.; Kaupmees, K.; Ovsjannikov, G.; Trummal, A.; Kütt, A.; Saame, J.; Koppel, I.; Kaljurand, I.; Lipping, L.; Rodima, T.; et al. Acidities of strong neutral Brønsted acids in different media. *Journal of Physical Organic Chemistry* **2013**, *26* (2), 162-170. DOI: https://doi.org/10.1002/poc.2946 (acccessed 2026/03/22).

(13) Eggins, B. R.; McNeill, J. Voltammetry of carbon dioxide: Part I. A general survey of voltammetry at different electrode materials in different solvents. *Journal of Electroanalytical*

Chemistry and Interfacial Electrochemistry **1983**, *148* (1), 17-24. DOI: https://doi.org/10.1016/S0022-0728(83)80127-2.
(14) Abraham, M. H.; Acree, W. E. Limiting Diffusion Coefficients for Ions and Nonelectrolytes in Solvents Water, Methanol, Ethanol, Propan-1-ol, Butan-1-ol, Octan-1-ol, Propanone and Acetonitrile at 298 K, Analyzed Using Abraham Descriptors. *Journal of Solution Chemistry* **2019**, *48* (5), 748-757. DOI: 10.1007/s10953-019-00884-0.
(15) Fernández, S.; Assaf, E. A.; Ahmad, S.; Travis, B. D.; Curley, J. B.; Hazari, N.; Ertem, M. Z.; Miller, A. J. M. Room-Temperature Formate Ester Transfer Hydrogenation Enables an Electrochemical/Thermal Organometallic Cascade for Methanol Synthesis from CO2. **2025**, *64* (4), e202416061. DOI: https://doi.org/10.1002/anie.202416061.
(16) Lanning, J. A.; Chambers, J. Q. Voltammetric study of the hydrogen ion/hydrogen couple in acetonitrile/water mixtures. *Analytical Chemistry* **1973**, *45* (7), 1010-1016. DOI: 10.1021/ac60329a002.
(17) Castro, M. T.; Chuang, P.-Y. A.; Ocon, J. D. - Multiphysics Modeling of a Low Voltage Acid-Alkaline Electrolyzer. **2022**.
(18) Gennaro, A.; Isse, A. A.; Vianello, E. Solubility and electrochemical determination of CO2 in some dipolar aprotic solvents. *Journal of Electroanalytical Chemistry and Interfacial Electrochemistry* **1990**, *289* (1), 203-215. DOI: https://doi.org/10.1016/0022-0728(90)87217-8.
(19) Matteo Agliuzza, C. F. P. a. A. S. A comprehensive modeling for the CO2 electroreduction to CO. *J. Phys. Energy* **2023**, *6*, 015004. DOI: 10.1088/2515-7655/ad0a39.
(20) Sampaio, R. N.; DiMarco, B. N.; Concepcion, J. J. Proton-Coupled Group Transfer Enables Concerted Protonation Pathways Relevant to Small-Molecule Activation. *Inorganic Chemistry* **2021**, *60* (22), 16953-16965. DOI: 10.1021/acs.inorgchem.1c01609.
(21) Stirling, M. J.; Sweeney, G.; MacRory, K.; Blacker, A. J.; Page, M. I. The kinetics and mechanism of the organo-iridium-catalysed enantioselective reduction of imines. *Organic & Biomolecular Chemistry* **2016**, *14* (14), 3614-3622, 10.1039/C6OB00245E. DOI: 10.1039/C6OB00245E.
(22) Gandler, J. R.; Bernasconi, C. F. Kinetics of deprotonation of arylnitromethanes by benzoate ions in acetonitrile solution. Effect of equilibrium and nonequilibrium transition state solvation on intrinsic rate constants of proton transfers. *Journal of the American Chemical Society* **1992**, *114* (2), 631-637. DOI: 10.1021/ja00028a032.
